# A manganese(i)tricarbonyl-catalyst for near room temperature alkene and alkyne hydroarylation†

**DOI:** 10.1039/d2sc04295a

**Published:** 2022-10-26

**Authors:** Shweta Choudhary, Diego M. Cannas, Matthew Wheatley, Igor Larrosa

**Affiliations:** Department of Chemistry, University of Manchester Oxford Road Manchester M13 9PL UK igor.larrosa@manchester.ac.uk

## Abstract

Developing more efficient catalytic processes using abundant and low toxicity transition metals is key to enable their mainstream use in synthetic chemistry. We have rationally designed a new Mn(i)-catalyst for hydroarylation reactions that displays much improved catalytic activity over the commonly used MnBr(CO)_5_. Our catalyst, MnBr(CO)_3_(MeCN)_2_, avoids the formation of the off-cycle manganacycle-(CO)_4_ species responsible for low catalyst activity, allowing near room temperature hydroarylation of alkenes and alkynes with broad functional group tolerance including late stage functionalisation and diversification of bioactive molecules.

## Abstract 2

A Mn(i)-catalyst for hydroarylation reactions, MnBr(CO)_3_(MeCN)_2_, avoids the formation of the off-cycle manganacycle-(CO)_4_ species responsible for low catalyst activity typical of MnBr(CO)_5_, leading to mild and broad scope hydroarylation.

## Graphical abstract

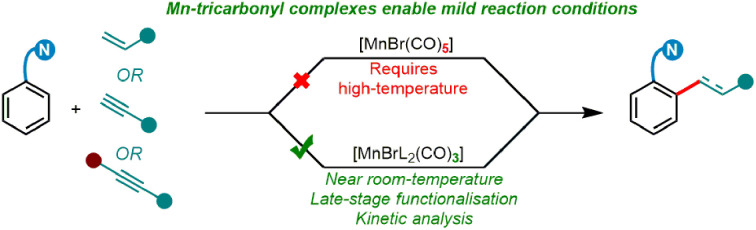

## Introduction

**Scheme 1:**
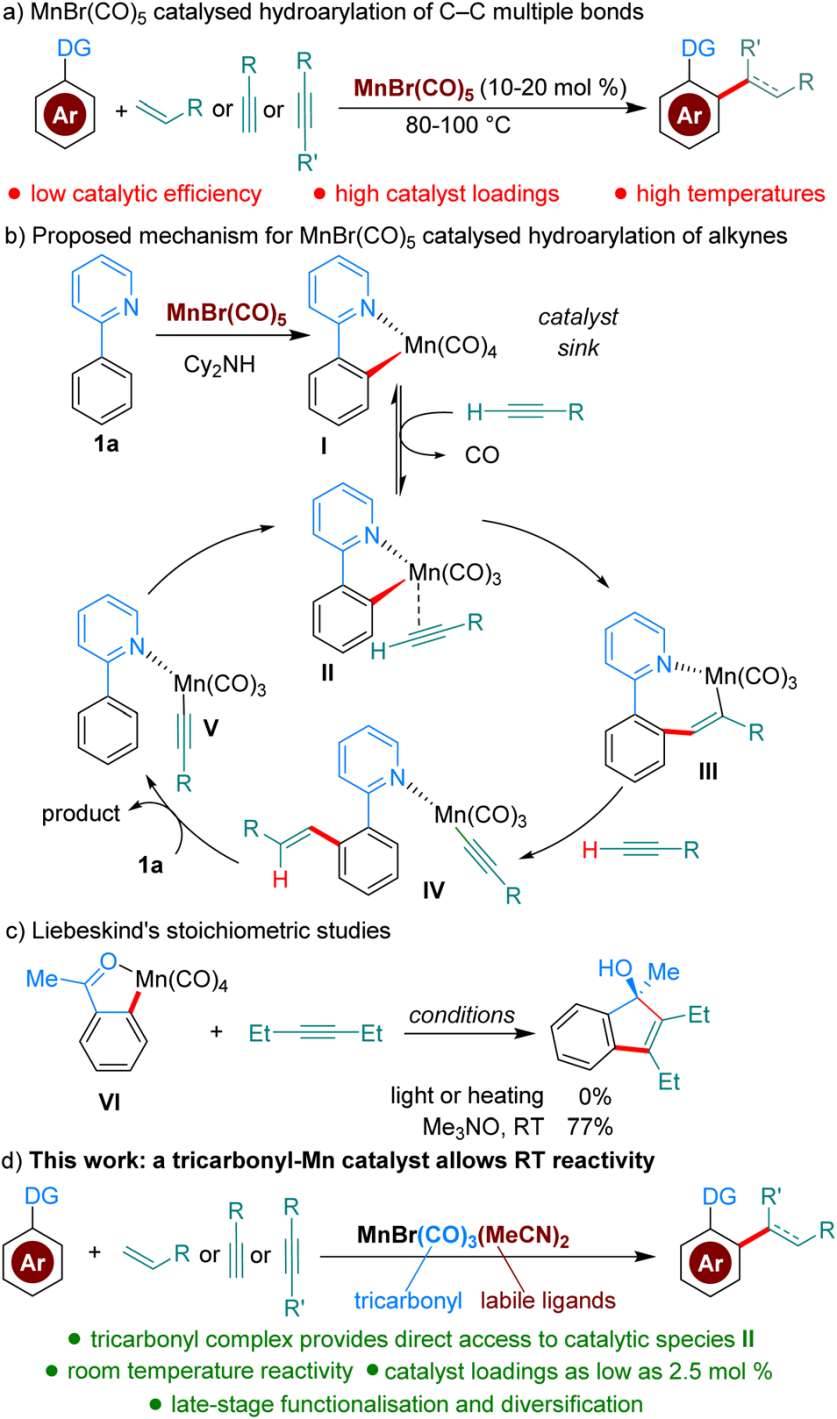
Mn-catalysed hydroarylations of C–C multiple bonds.

C–H functionalisation bears the promise of streamlining synthetic organic procedures, resulting in more sustainable processes.^1^ The field of C–H functionalisation has traditionally been dominated by precious metals; however, in recent years many base-metal catalysed processes have been developed.^2^ Mn in particular, the third most abundant transition metal and of low toxicity, has received much attention as a catalyst.^2*a*,3^ Mn-catalysed hydroarylation reactions of C–C multiple bonds are among the most studied methods (Scheme 1a). However, these reactions, which are almost invariably catalysed by either MnBr(CO)_5_ or Mn_2_(CO)_10_, suffer from low catalytic activity, resulting in the requirement of elevated temperatures (80–100 °C) and high catalyst loadings (commonly 10–20 mol%).^4^ Based on computational and experimental studies, Wang and co-workers have proposed a mechanism where MnBr(CO)_5_ is a precatalyst forming off-cycle species I (Scheme 1b), which must dissociate CO and coordinate the alkyne coupling partner to enter the catalytic cycle as II. The authors calculated that the formation of II from I is disfavored by 22.5 kcal mol^−1^.^5^ More recently, Fairlamb, Lynam and co-workers were able to further experimentally characterize, with time-resolved infrared spectroscopy, the dissociation and association processes going from I to III, by facilitating the dissociation of CO through photochemical activation.^6^ Interestingly, Liebeskind showed in 1989 that Mn(CO)_4_-complex VI is unreactive towards alkynes.^7^ However, when a stoichiometric amount of a CO scavenger is added, the desired product is formed at room temperature, demonstrating the higher reactivity of Mn(CO)_3_-species. The group of Woodgate further expanded on these studies, using stoichiometric Mn-complexes and CO scavengers in the modification of steroid derivatives.^8^

Based on the studies described above, we reasoned that in the reaction conditions for hydroarylation, Mn(CO)_4_-species I are acting as an off-cycle catalyst ‘sink’ responsible for the low catalyst efficiency. We hypothesised that a stable MnXL_2_(CO)_3_-species with appropriate L ligands, could result in significantly enhanced catalytic activity, by avoiding altogether the formation of off-cycle species I. To the best of our knowledge Mn(CO)_3_-complexes have never been reported as catalysts for hydroarylation reactions. Herein, we describe a novel catalytic hydroarylation that takes advantage of a Mn-complex capable of mediating these reactions at room temperature or near room temperature, overcoming one of the current bottlenecks in efficient Mn-catalysis (Scheme 1d).

## Results and discussion

To test our hypothesis, we chose as a benchmark the reaction between 2-phenylpyridine, 1a, and butyl acrylate, 2a, previously requiring 100 °C when catalysed by MnBr(CO)_5_. We began our studies by synthesising several Mn(i)-tricarbonyl complexes to test in our reaction manifold. Gratifyingly, upon testing these complexes at 35 °C, we found that all of them displayed superior reactivity to MnBr(CO)_5_ (see ESI,†Table 1). In particular, the air-stable tricarbonyl Mn-complex MnBr(CO)_3_(MeCN)_2_ ^9^ performed exceptionally well, with 3aa being formed in 98% yield (Table 1, entry 1). Upon testing the current state-of-the-art MnBr(CO)_5_ complex (entry 2) we found that only trace product formation occurred, supporting our hypothesis that Mn-tricarbonyl complexes would exhibit superior catalytic activity. On the other hand, [Mn(CO)_3_(MeCN)_3_]PF_6_, where the Br-ligand is replaced with a non-coordinating PF_6_^−^, led to lower reactivity (entry 3). When the reaction temperature was lowered to 25 °C the product was delivered in a respectable 50% yield (entry 4). Lowering the catalyst loading had a small appreciable impact (entry 5), with 3aa being formed in 70% yield. Remarkably, the reaction can be run even with only 2.5 mol% catalyst by raising the temperature to 100 °C (entry 6), affording a 70% yield of 3aa. To the best of our knowledge, this is the lowest catalyst loading reported for a Mn-catalysed acrylate hydroarylation. A brief survey of ethereal solvents (entries 7–9) revealed that both *i*Pr_2_O, THF and 1,4-dioxane performed poorly when compared with Et_2_O. Following this, an additive screen (entries 10–11) revealed Cy_2_NH to perform significantly better than Et_3_N and KOAc. The use of Cy_2_NH has been studied extensively in these catalytic manifolds by the groups of Fairlamb and Wang and has been shown to have a positive effect on Mn-catalysed hydroarylation processes.^4*d*,5,10^

**Table 1:** Optimisation of reaction. Yields determined by GC-FID using hexadecane as an internal standard

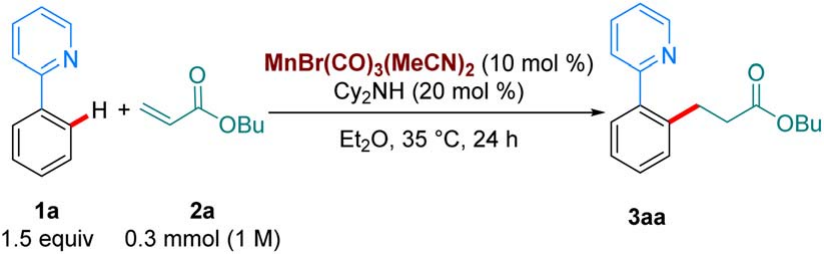		
Entry	Variation from standard conditions	3aa (%)
1	—	98
2	MnBr(CO)_5_ (10 mol%) as cat	Trace
3	[Mn(CO)_3_(MeCN)_3_]PF_6_ (10 mol%) as cat	51
4	25 °C instead of 35 °C	50
5	5 mol% MnBr(CO)_3_(MeCN)_2_	70
6	2.5 mol% MnBr(CO)_3_(MeCN)_2_ at 100 °C	70
7	*i*Pr_2_O as solvent	62
8	THF as solvent	57
9	1,4-Dioxane as solvent	72
10	Et_3_N (20 mol%) as additive	44
11	KOAc (20 mol%) as additive	Trace

**Scheme 2:**
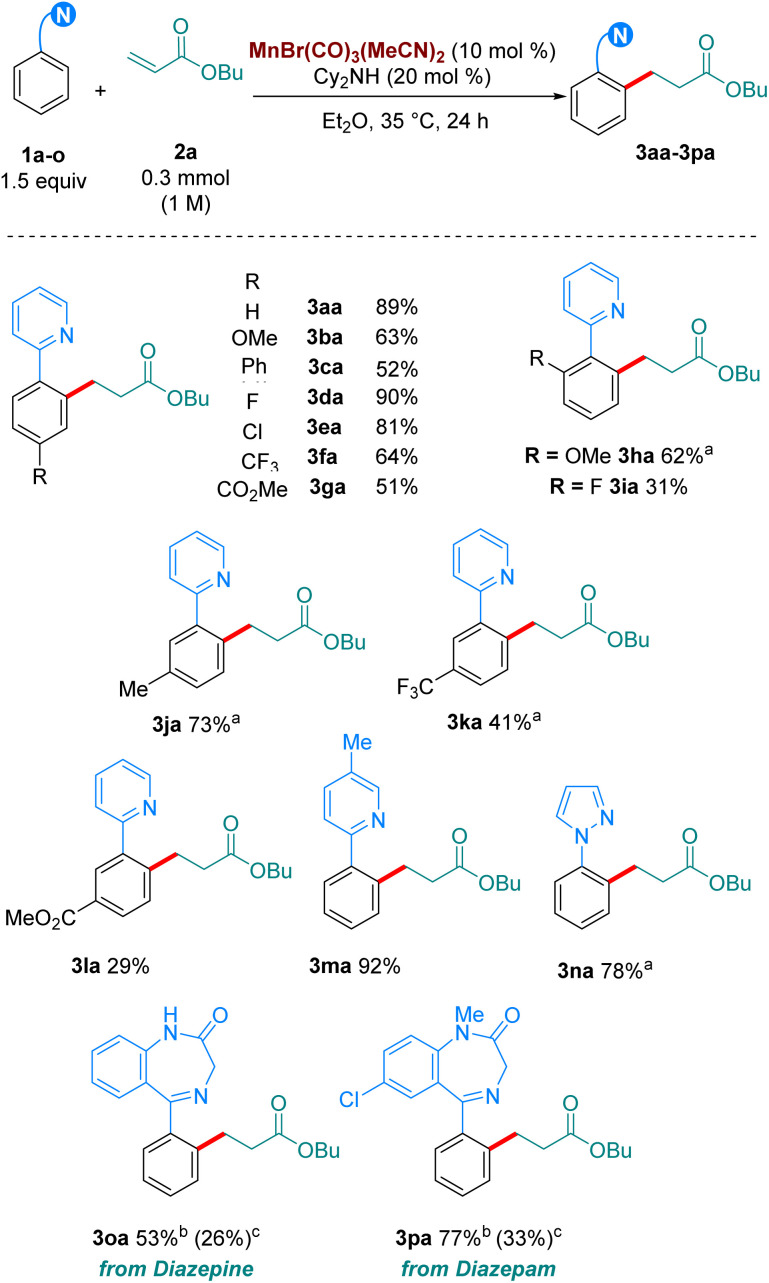
Scope of the hydroarylation of 2a with arenes. All yields are of isolated pure compound. ^a^ The reaction was carried out for 72 h instead of 24 h. ^b^ With 1o (0.2 mmol), 2a (2 equiv.), Mn cat (20 mol%), Cy_2_NH (20 mol%) in Et_2_O. The reaction was stirred at 35 °C for 72 h. ^c^ Using MnBr(CO)_5_ as catalyst (10 mol%) with Cy_2_NH (20 mol%) at 100 °C in Bu_2_O (0.5 M).

With our optimised reaction conditions in hand, we turned our attention to examining the scope of the reaction (Scheme 2). Pleasingly, the reaction performed well with electron-donating (3ba), neutral (3ca) and electron withdrawing substituents (3da-3ga) at the *para* position of the aromatic ring. Electron-donating and withdrawing substituents were also tolerated at the *ortho* position (3ha-3ia). Lower reactivity was observed when these substituents were at the *meta* position (3ja-3la). Synthetically relevant pyrazoles and diazepines (imines) were shown to be competent directing groups, with 3na and 3oa being formed in good yields. Diazepam, an anxiolytic drug, was successfully late-stage alkylated, with 3pa being formed in excellent yield and selectivity.

**Scheme 3:**
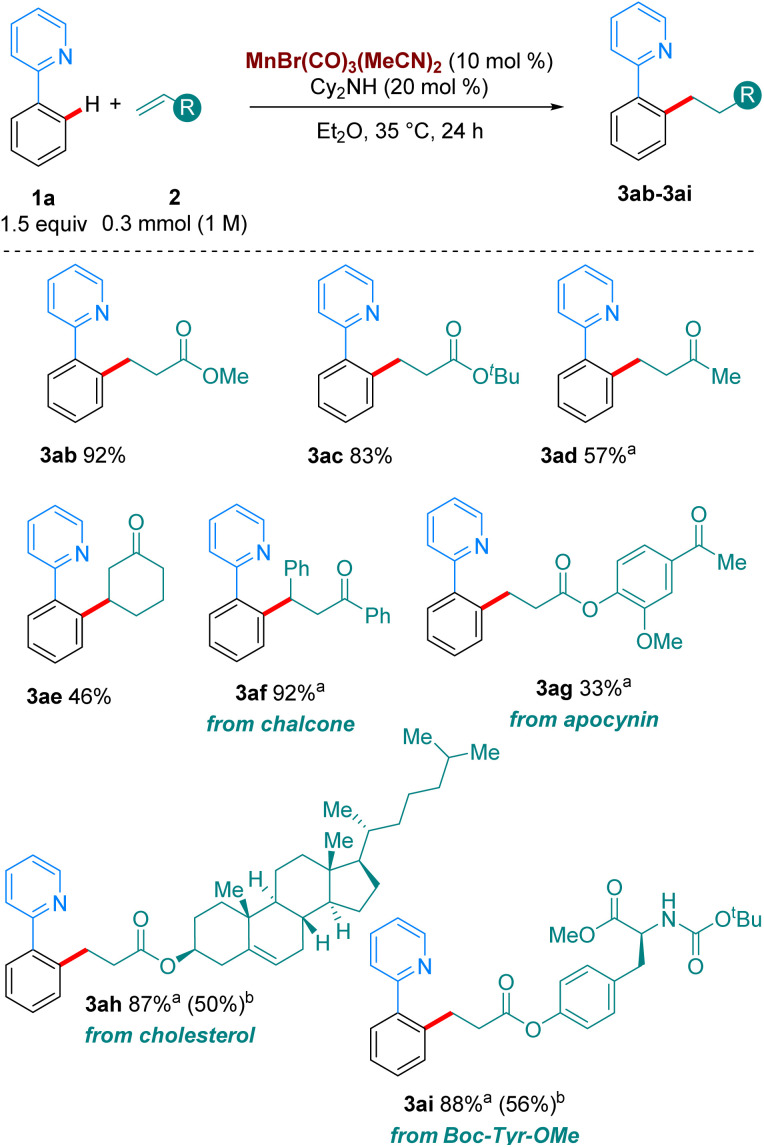
Scope of the hydroarylation of olefins with 1a. All yields are of isolated pure compound. ^a^ The reaction was carried out for 72 h instead of 24 h. ^b^Using MnBr(CO)_5_ as catalyst (10 mol%) with Cy_2_NH (20 mol%) at 100 °C in Bu_2_O.

Next, we turned our attention to the scope in alkene coupling partner (Scheme 3). We found that in our optimised reaction conditions, several olefins could react to form the corresponding alkylated products. Acyclic α,β-unsaturated ketone 2d gave 3ad in respectable yields. Interestingly, internal olefins, both cyclic and acyclic, also reacted well, with 3ae and 3af delivered in 46 and 92% yield, respectively. However, in line with most of the high temperature versions of this method,^4^ only electron-deficient olefins are suitable partners. When the alkene coupling partners were electron-rich, only trace amounts of product could be detected. Complex olefins 2g, 2h and 2i, derived from apocynin, cholesterol and Boc-Tyr-OMe, respectively, reacted in our system in moderate to excellent yields, with 3ag, 3ah and 3ai formed in 33%, 87% and 88%, respectively. Importantly, when we carried out the reactions of 1o, and 1p with 2a, as well as those of 1a with 2h and 2i using MnBr(CO)_5_ at 100 °C much lower yields were obtained of the corresponding products, 3oa, 3pa, 3ah and 3ai. These results emphasize the synthetic advantage provided by the use of the MnBr(CO)_3_(MeCN)_2_ at moderate temperatures.

**Scheme 4:**
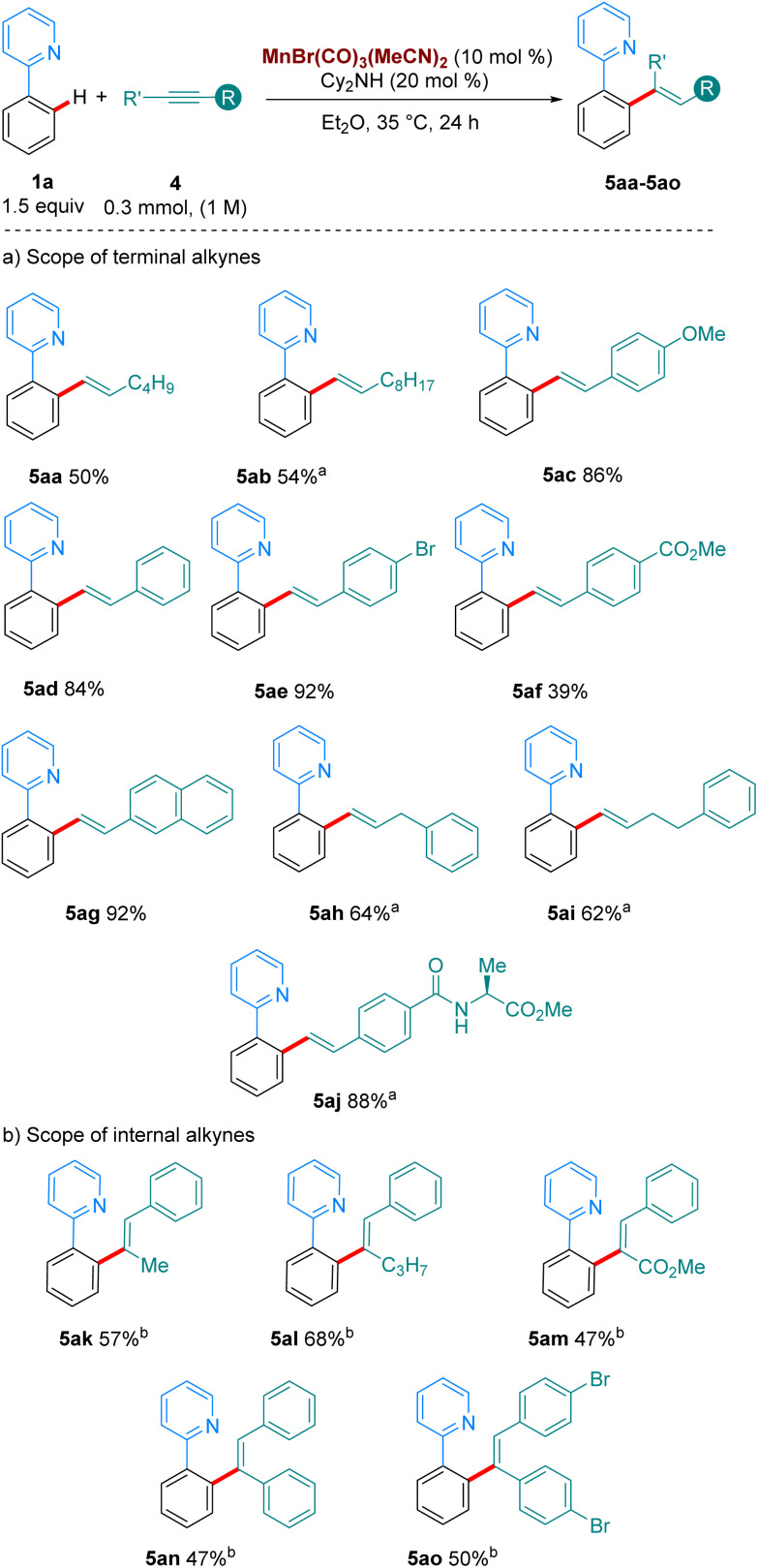
Scope of the hydroarylation of alkynes with 1a. All yields are of isolated pure compound. ^a^ The reaction was carried out for 72 h instead of 24 h. ^b^ With 1a (0.3 mmol), 4k-o (1.5 equiv.), Mn cat (10 mol%), Cy_2_NH (30 mol%) and 4-CF_3_-benzoic acid (20 mol%) in Et_2_O. The reaction was stirred at 35 °C for 72 h.

We then turned our attention to the hydroarylation of alkynes with our Mn(i)-tricarbonyl catalyst (Scheme 4). Gratifyingly, aliphatic and aromatic terminal alkynes also reacted well at 35 °C (5aa–5af, Scheme 4a), showcasing the superior catalytic activity of manganese tricarbonyl complexes over the classic MnBr(CO)_5_ complex with functional groups such as OMe, Br, esters, amides and amino acids tolerated in the reaction. Finally, internal alkynes were examined as coupling partners in the reaction (Scheme 4b). Previous work by the group of Fairlamb notes that these substrates have proven to be unproductive in the absence of an acidic additive, due to the favoured protonation of the organometallic intermediate rather than reductive elimination to yield the alkenylated products.^6*a*,9^ We were pleased to find that with the addition of 20 mol% of 4-CF_3_-benzoic acid we could access the products of these reactions in good yields also at 35 °C. Unsymmetrical alkynes bearing aryl/alkyl or aryl/carboxymethyl led to completely regioselective addition, forming 5ak–5am.

**Scheme 5:**
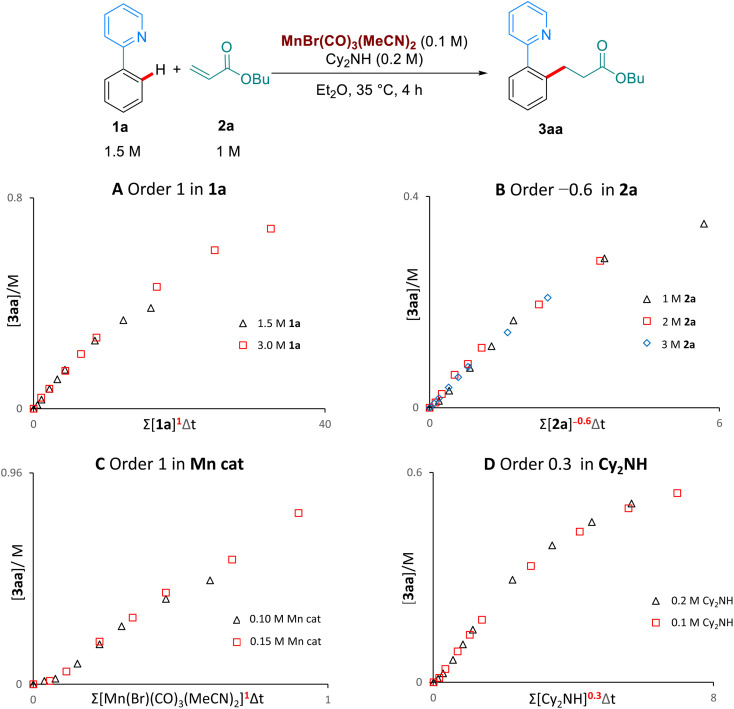
Kinetic analysis by VTNA.

We then examined the kinetic orders of the reaction components, in the reaction of 1a with 2a, using the VTNA method developed by Burés^11^ (Scheme 5). These studies revealed first order kinetics on the Mn-catalyst and on 1a, as well as a partial order of 0.3 on Cy_2_NH. Interestingly, we observed a significantly slower reaction when increasing the concentration of the butyl acrylate substrate 2a, corresponding to an inverse order of −0.6. To the best of our knowledge this inverse dependence on the concentration of alkene has never been observed in the Mn-catalysed hydroarylation of olefins, and suggests that multiple coordinations of the alkene are possible to form an off-cycle species of the type [Mn(2a)_2_]. Lynam and Fairlamb have previously shown that 2a can coordinate to Mn through both oxygen and the olefin.^6*b*^

**Scheme 6:**
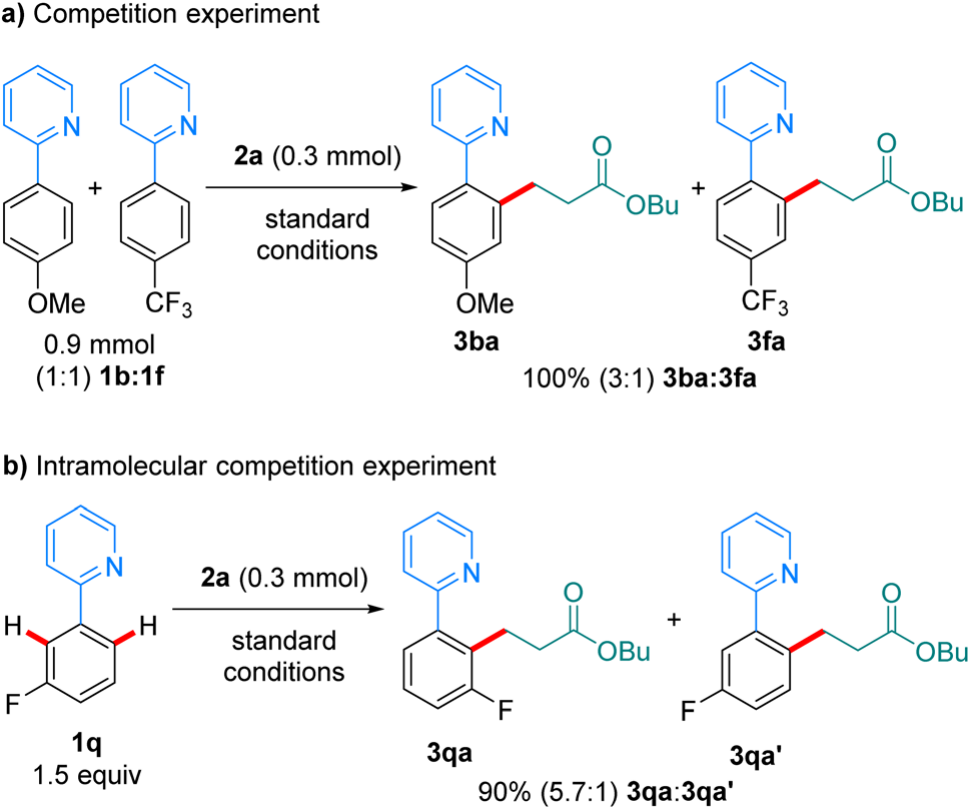
Competition experiments.

In a competition experiment between 1b and 1f in their reaction with 2a (scheme 6a), the more electron-rich arene reacted preferentially, with 3ba being formed over 3fa in a 3 : 1 ratio. This could be explained by preferential coordination to the Mn-catalyst of the more electron-rich 1b. An intramolecular competition experiment using substrate 1q showed preferential reaction on the most acidic C–H bond (Scheme 6b). The observed regioselectivity (5.7 : 1) closely resembles that recently reported by Lynam and Fairlamb on the hydroarylation of 1q with phenylacetylene,^12,13^ and suggests that the C–H activation step is irreversible under the reaction conditions.

## Conclusions

We have demonstrated that MnBr(CO)_3_(MeCN)_2_ is a highly active catalyst for the hydroarylation of electron-deficient olefins, and of terminal and internal alkynes. This procedure has a broad functional group tolerance, owing to the mild conditions enabled by the catalyst. This method has been applied to the late-stage diversification and functionalisation of natural products and pharmaceutical molecules, displaying its synthetic utility potential. Preliminary mechanistic studies suggest that the C–H activation step is irreversible and that high concentrations of the alkene coupling partner have a deleterious effect on reactivity. The high reactivity of Mn-tricarbonyl complexes compared to MnBr(CO)_5_, is attributed to the prevention of formation of Mn(CO)_4_-species, previously proposed as an off-cycle catalyst sink. The exploration of further applications of this new Mn-catalyst are under way.

## Data availability

All experimental procedures and compound characterization can be found in the ESI.†

## Author contributions

D. M. C. conceived the project and carried out initial discovery work and optimisation. S. C. isolated and characterised all compounds and contributed to the optimisation of the reaction. M. W. assisted S. C. with the running of the project and compound isolation. I. L. secured the funding used in this study and directed the work. M. W. and I. L. prepared the manuscript with input from S. C.

## Conflicts of interest

There are no conflicts to declare.

## Supplementary Material

SC-013-D2SC04295A-s001
