# Supplementary material for: A manganese(i)tricarbonyl-catalyst for near room temperature alkene and alkyne hydroarylation
Source: Chem Sci. 2022 Oct 26;13(44):13225–30. doi: 10.1039/d2sc04295a (PMC9667916; doi:10.1039/d2sc04295a)
Supplement: SC-013-D2SC04295A-s001 [file SC-013-D2SC04295A-s001.pdf]

Electronic Supplementary Material (ESI) for Chemical Science.
This journal is © The Royal Society of Chemistry 2022

A Manganese(I)tricarbonyl-Catalyst for Near-Room-Temperature Alkene and Alkyne Hydroarylation

Shweta Choudhary, Diego M. Cannas, Matthew Wheatley, and Igor Larrosa*

Department of Chemistry, University of Manchester, Oxford Road, Manchester M13 9PL, U.K.

Supporting Information

Table of Contents

1. General Experimental Details 2
2. Preparation of [$MnBr(CO)_3(MeCN)_2$] 2
3. General Procedures 4
A. Ester formation from acrylic acid and alcohol 4
B. Hydroarylation of alkenes and terminal alkynes 4
C. Hydroarylation of internal alkynes 4
4. Substrate Scope 5
A. Scope of *N*-directing heteroarene 5
B. Scope of alkene 6
C. Scope of terminal alkynes 7
D. Scope of internal alkynes 7
5. Optimisation Results 8
A. Catalyst screening 8
B. Additive screening 9
C. Temperature Screening and catalyst loading 10
D. Solvent Screening 11
E. Reaction optimisation for internal alkyne substrates 12
F. Failed arenes 13
G. Failed alkene coupling partners 12
6. Characterisation Data 14
7. Mechanistic Studies 36
7.1 Competition experiments between electron-rich and poor aromatics 36
7.2 Kinetic Concentration Sensitivity Experiments 36
7.3 Kinetic Experiments for determination of orders 37
8. Copies of ^1H and ^{13}C NMR for isolated Compounds 43
9. References 95

1. General Experimental Details

All reagents and starting materials were purchased from commercial sources and were used without further purification. $MnBr(CO)_5$ was purchased from alfa aesar. Mn(I) complexes were prepared as described in literature.[1-4] $MnBr(CO)_3(MeCN)_2$ was prepared in absence of light under inert conditions, and stored in an argon-filled glovebox. All hydroarylation reactions were set up inside an argon-filled glovebox. All liquid reagents and solvents were dried over 4 Å molecular sieves and degassed with 3 freeze-pump-thaw cycles. Purification of crude was carried out using silica gel based flash chromatography. ^{1}H NMR, ^{19}F NMR and ^{13}C NMR spectra were recorded at 400 or 500 MHz on Bruker instruments. ^{1}H NMR are referenced to the residual solvent peak at 7.26 ppm ($CDCl_3$) or 2.50 ppm DMSO-d_6 ppm values are quoted to 2 decimal places, with coupling constants (J) to the nearest 0.1 Hz. ^{13}C NMR spectra were recorded at 126 or 101 MHz and quoted in ppm to 1 decimal place with coupling constants (J) to the nearest 0.1 Hz. The spectra were referenced to the residual solvent peak at 77.16 ppm (CDCl3) or 39.52 ppm DMSO-d_6. ^{19}F NMR spectra recorded at 376 MHz in $CDCl_3$ and quoted in ppm to 1 decimal place with coupling constants (J) to the nearest 0.1 Hz. Mass spectra were performed by the School of Chemistry Mass Spectrometry Service (University of Manchester) employing a Thermo Finnigan MAT95XP spectrometer. IR spectra were recorded using a Bruker alpha platinum ATR machine; relevant bands are quoted in cm^{-1}.

2. Preparation of $MnBr(CO)_3(MeCN)_2$

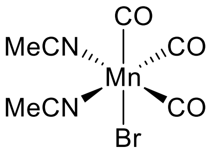

$MnBr(CO)_5$ (1.0 g, 3.6 mmol) was dissolved in hexane (50 mL). To this, dry acetonitrile (0.64 mL) was added and refluxed in the absence of light under nitrogen for 1.5 h. After completion, the reaction mixture was concentrated *in vacuo*. The precipitate was filtered under an inert atmosphere and the resulting yellow solid was washed with hexane to obtain $MnBr(CO)_3(MeCN)_2$ as a yellow solid (917 mg, 85%).[1] Anal. Calcd. for $MnBr(CO)_3(MeCN)_2$: C 27.93; H 2.01; N 9.31; Mn 18.25. Found C 27.08; H 1.73; N 8.20; Mn 18.36. **^{1}H NMR** (500 MHz, DMSO-d_6) δ 2.05 (s, 6H). **^{13}C NMR** (126 MHz, DMSO-d_6) δ 224.2, 220.4, 117.4, 0.6. **IRν_{max}** (neat/cm^{-1}): 2979, 2324, 2033, 1920, 1368, 1035, 674, 625; **IRν_{max}** (KBr/cm^{-1}): 2304, 2270, 2044, 1941, 1926, 676, 624; **HRMS** calculated for $[C_7H_6O_3N_2BrMnNa]^+$: 322.8835, found 322.8833. Note: the chemical shifts for the MeCN ligands appear at a similar value to those for free MeCN. This could be due to displacement by DMSO. The complex was insoluble in other common NMR solvents.

Yellow needle-shaped crystals were obtained by recrystallisation from MeCN/Hexane in a glovebox at ambient temperature. Data was obtained using XRD core facility, Rigaku FR-X Left, Rigaku FR-X Right, SuperNova, Oxford X'Calibur, Bruker D8 Advance, Phillips X'Pert, X ray Single Crystal Structure Determination Service, X-ray Power Diffraction data collection service, Diamond Collection Required, X-ray Air-Sensitive Single Crystal Structure Determination. X-ray data matches those reported.[5] Previously reported data can be obtained from X-ray data base using Identifier: BTMICM, CCDC 1115637 via www.ccdc.cam.ac.uk/conts/retrieving.html (or from the

Cambridge Crystallographic Data Centre, 12 Union Road, Cambridge CB21EZ, UK; fax: (+44)1223-336-033; or deposit@ccdc.cam.ac.uk).

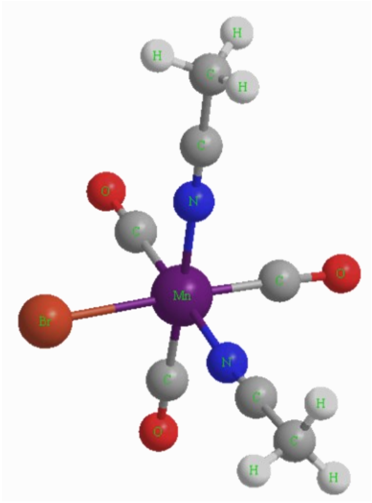

Formula	$C_7H_6BrMnN_2O_3$
Mr	300.99
Temperature	100 K
Cell	a=6.1549(4)
	b=8.7215(7)
	c=10.7979(7)
	α=81.729(6)
	β=81.123(6)
	γ=70.688(7)
Volume	537.78(7)
Z	2
Dx,g cm-3	1.859
F000	292.0
Nref	2567
R(reflections)	0.0207(2376)
wR2(reflections)	0.0496(2567)
Wavelength/Å	0.71073
Theta(max)	30.672
Tmin	0.719
Tmax	1.000

3. General Procedures

A. Ester formation from acrylic acid and alcohol

In a round bottom flask, the corresponding alcohol (1.0 equiv), EDC (1.5 equiv) and DMAP (10 mol%) were dissolved in CH_2Cl_2 (0.5 M). To the reaction mixture, a solution of acrylic acid (1.1 equiv) in CH_2Cl_2 (2 M) was added slowly and stirred overnight at room temperature. After completion the reaction was diluted with water and extracted with CH_2Cl_2 (3 × 5.0 mL/mmol). The organic extracts were combined and washed with brine (5.0 mL/mmol), dried over Na_2SO_4 and concentrated *in vacuo*. This residue was purified using silica gel chromatography.

B. Hydroarylation of alkenes and terminal alkynes

In an argon-filled glove box, $MnBr(CO)_3(MeCN)_2$ (10 mol %, 9.0 mg) catalyst was added to an oven dried microwave vial containing a magnetic stirrer bar, followed by addition of *N*-directing group arene **1** (1.5 equiv), electrophile **2a-h**, or **4a-j** (0.3 mmol), and Cy_2NH (20 mol %) dissolved in Et_2O (1 M). The vial was sealed, taken out of the glove box and the reaction stirred at 35 °C for 24 h. After completion the reaction mixture was filtered through a cotton plug, washed with Et_2O and concentrated *in vacuo*. This material was purified using silica gel chromatography to obtain the desired product.

C. Hydroarylation of internal alkynes

In an argon-filled glove box, $MnBr(CO)_3(MeCN)_2$ (10 mol %, 9.0 mg) catalyst was added to an oven dried microwave vial containing a magnetic stirrer bar, followed by addition of 4-CF_3-benzoic acid (20 mol %), **1a** (0.3 mmol), internal alkyne **4k-o** (1.5 equiv), and Cy_2NH (30 mol %) dissolved in Et_2O (1 M). The vial was sealed, taken out of the glove box and stirred at 35 °C for 72 h. After completion the reaction was filtered through a cotton plug, washed with Et_2O and concentrated *in vacuo*. This material was purified using silica gel chromatography to obtain the desired product.

4. Substrate Scope

A. Scope of *N*-directing heteroarene

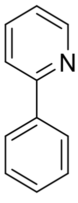

1a

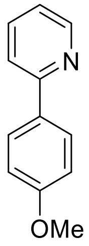

1b

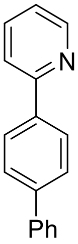

1c

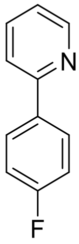

1d

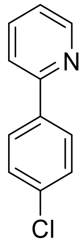

1e

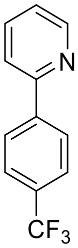

1f

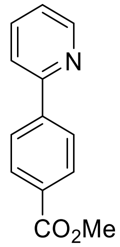

1g

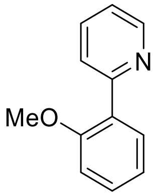

1h

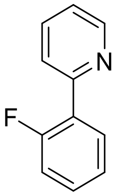

1i

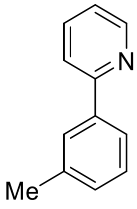

1j

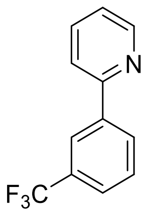

1k

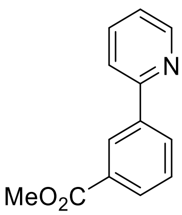

1l

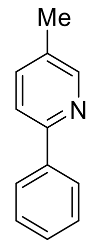

1m

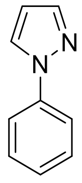

1n

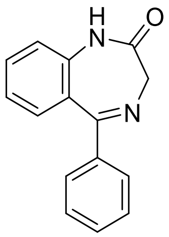

1o

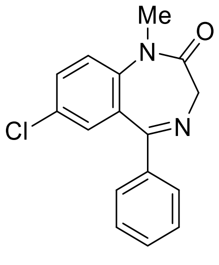

1p

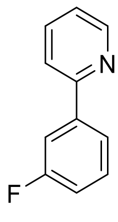

1q

Compounds **1a, 1n, 1o, 1p** are commercially available and were used without further purification. The following substrates were prepared according to procedures described in literature: **1b**,[6] **1c**,[6] **1d**,[6] **1e**,[6] **1f**,[6] **1g**,[6] **1h**,[7] **1i**,[8] **1j**,[6] **1k**,[6] **1l**,[6] **1m**.[9]

B. Scope of alkene

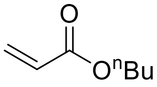

2a

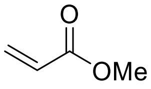

2b

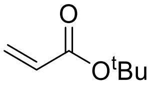

2c

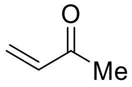

2d

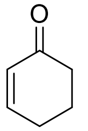

2e

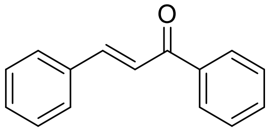

2f

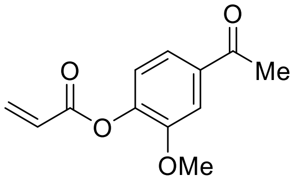

2g

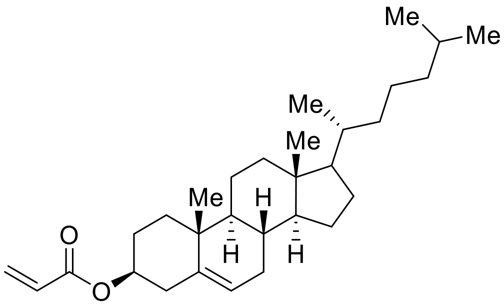

2h

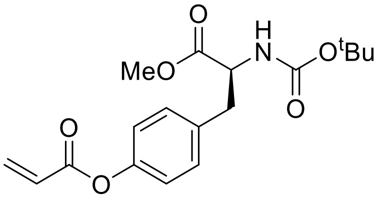

2i

Compounds **2a, 2b, 2c, 2d, 2e, 2f** are commercially available and were used without further purification.

The following substrates were prepared according to general procedure A: **2g, 2h, 2i**.

C. Scope of terminal alkynes

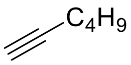

4a

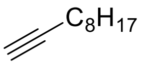

4b

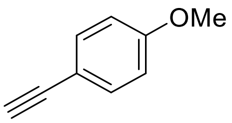

4c

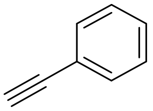

4d

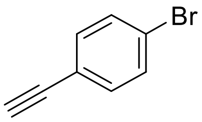

4e

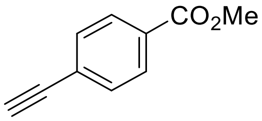

4f

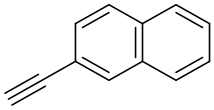

4g

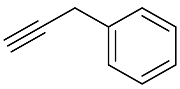

4h

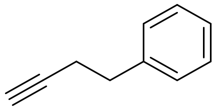

4i

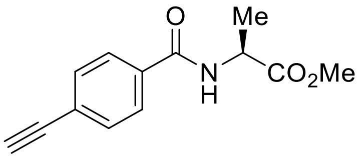

4j

Compounds **4a, 4b, 4c, 4d, 4e, 4f, 4g, 4h, 4i** are commercially available and used without further purification.
4j[11] was prepared according to procedure described in literature.

D. Scope of internal alkynes

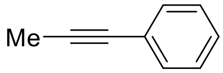

4k

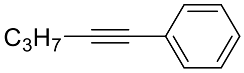

4l

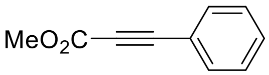

4m

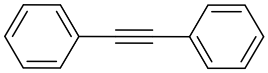

4n

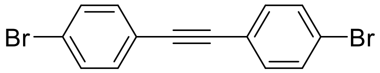

4o

Compounds **4k, 4l, 4m, 4n, 4o,** are commercially available and used without further purification.

5. Optimisation Results

A. Catalyst screening

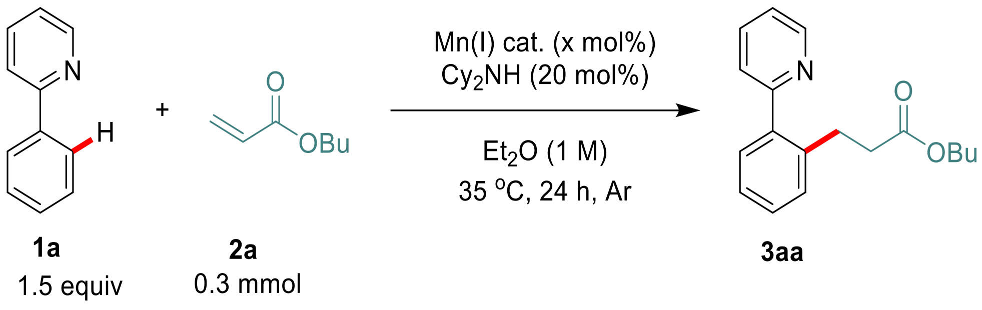

Entry	Mn(I) Cat	Catalyst Loading (x mol%)	1a(%)	3aa(%)
**1**	$[MnBr(CO)_5]$	10	99	<1
**2**	$[Mn(CO)_3(MeCN)_3]PF_6$	10	86	51
**3**	**$MnBr(CO)_3(MeCN)_2$**	**10**	**40**	**98**
**4**	$[Mn(CO)_3(naphthalene)]BF_4$	10	95	48
**5**	$[Mn_2(\mu{-}Br)_3(CO)_6]NEt_4$	5	96	14
**6**	$[Mn(CO)_5(OTf)]$	10	85	11

Table 1. Results of the preliminary catalyst screening. Yields determined by GC-FID using hexadecane as an internal standard.

B. Additive screening

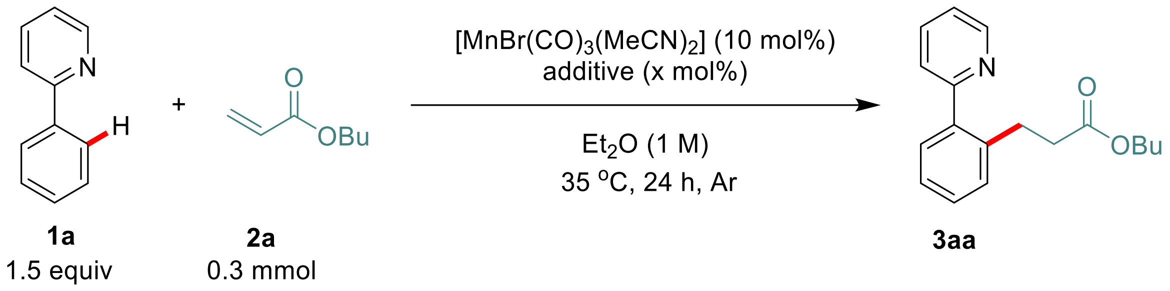

Reaction	Additive (mol%)	1a(%)	3aa(%)
**1**	Et_3N (20%)	84	44
**2**[a]	Et_3N (20%)	80	45
**3**	Et_3N (30%)	58	74
**4**	**Cy_2NH (20%)**	**40**	**98**
**5**	Cy_2NMe (20%)	81	32
**6**	KOAc (20%)	125	1

Table 2. Results of the additive screen. Yields determined by GC-FID using hexadecane as an internal standard. [a]Reaction performed in absence of light.

C. Temperature Screening and catalyst loading

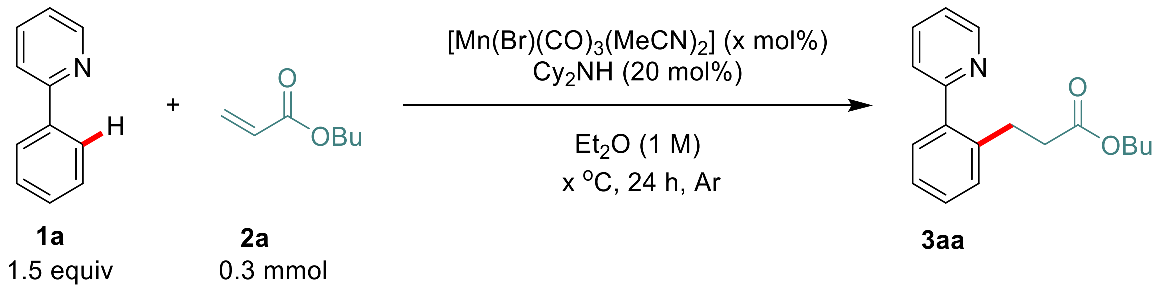

Entry	Cat loading (x mol%)	Temperature (x °C)	1a(%)	3aa(%)
**1**	**10**	**35**	**40**	**98**
**2**	10	25	71	50
**3**	5	35	62	70
**4**	5	50	53	84
**5**	5	80	41	98
**6**	2.5	50	113	21
**7**	2.5	80	70	56
**8**	2.5	100	68	70

Table 3. Results of the temperature testing reactions with the $[MnBr(CO)_3(MeCN)_2]$ catalyst. Yields determined by GC-FID using hexadecane as an internal standard.

D. Solvent Screening

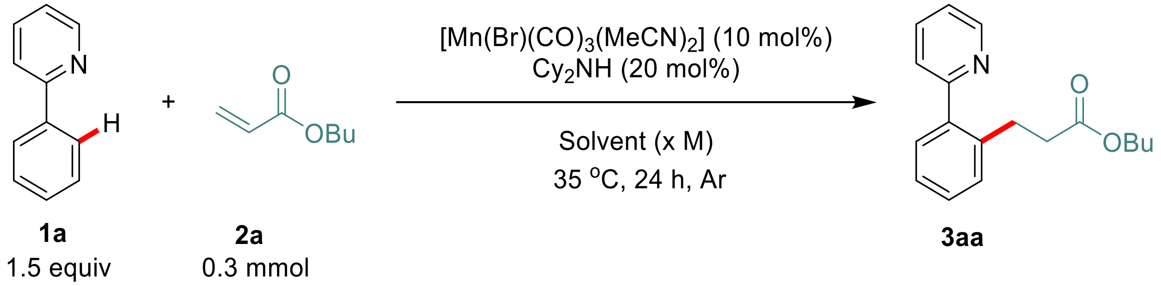

Reaction	Solvent	Concentration/ (M)	1a (%)	3aa (%)
**1**	Et_2O	(0.5)	60	65
**2**	**Et_2O**	**(1.0)**	**40**	**98**
**3**	$^iPr_2O$	(1.0)	62	62
**4**	PhMe	(1.0)	49	79
**5**	THF	(1.0)	66	57
**6**	1,4 Dioxane	(1.0)	59	72

Table 4. Results of varying reaction concentration experiment and reaction solvent. ^{1}H NMR yields obtained using 1,3,5-trimethoxybenzene as an internal standard.

E. Reaction optimisation for internal alkyne substrates

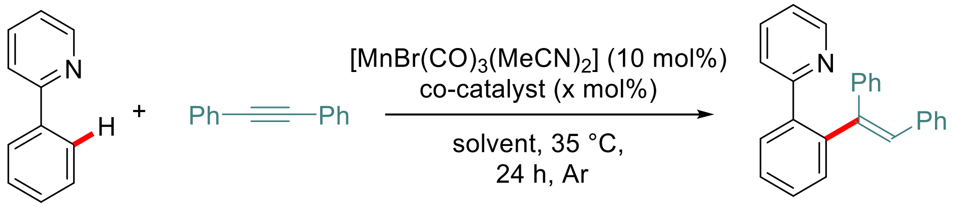

Entry	1a (x mmol)	4n (x mmol)	Solvent	Co-catalyst (x mol%)	1a (%)	5an (%)
**1**	0.15	0.1	Et_2O (0.5 M)	K-m-NO_2benzoate 20%	127	28
**2**	0.1	0.15	Et_2O (0.5 M)	K-m-NO_2benzoate 20%	81	22
**3**	0.1	0.15	Et_2O (1 M)	K-m-NO_2benzoate 20%	80	23
**5**	0.1	0.15	Et_2O (0.5 M)	3-(NO_2)benzoic acid 20% + Cy_2NH 20%	68	41
**6**	0.1	0.15	Et_2O (0.5 M)	3-CF_3benzoic acid 20% + Cy_2NH 30%	69	31
**7**	0.1	0.15	Et_2O (0.5 M)	Benzoic acid 20% + Cy_2NH 20%	73	36
**8**	0.1	0.15	Et_2O (0.5 M)	2,6-difluorobenzoic acid 20% + Cy_2NH 20%	83	19
**9**	0.1	0.15	Et_2O (0.5 M)	3,5-$(NO_2)_2$benzoic acid 20% + Cy_2NH 20%	76	30
**10**	0.1	0.15	Et_2O (0.5 M)	4-(NMe_2)benzoic acid 20% + Cy2NH 20%	92	8
**11**	**0.1**	**0.15**	**Et_2O (1 M)**	**4-(CF_3)benzoic acid 20% + Cy_2NH 30%**	**52**	**48**
**12**	0.1	0.15	Et_2O (0.5 M)	$PhP(O)(OH)_2$ 10% + Cy_2NH 20%	97	0

Table 5. Results of the solvent, additive screening reactions. Yields determined by GC-FID using hexadecane as an internal standard.

F. Failed arenes

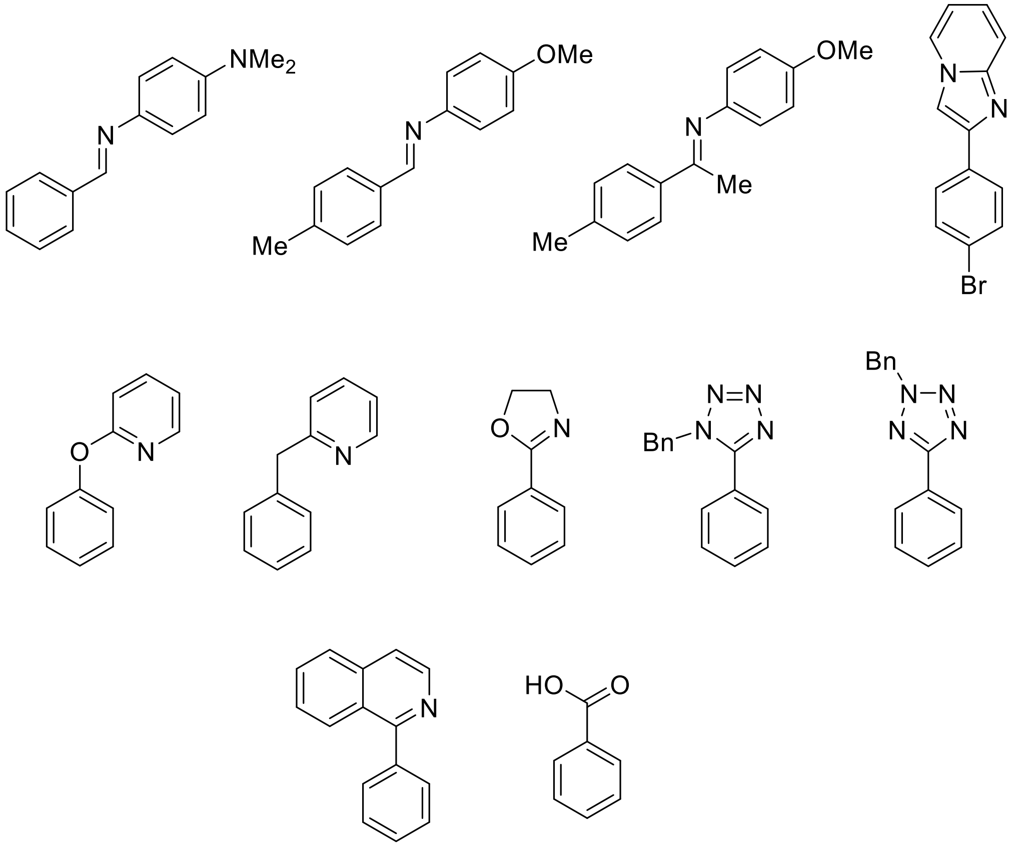

G. Failed alkene coupling partners

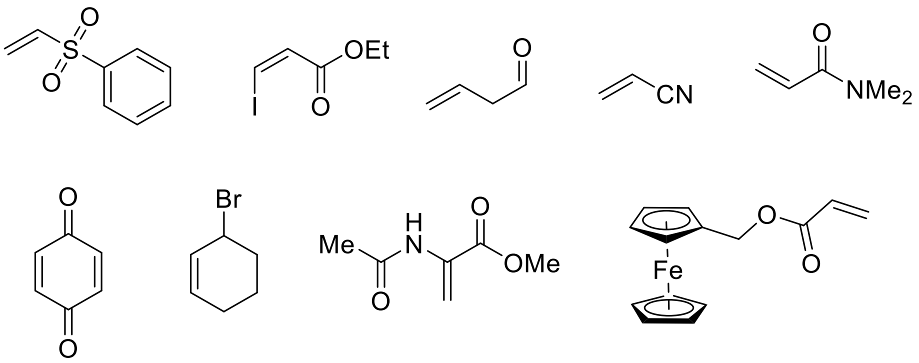

6. Characterisation Data

4-acetyl-2-methoxyphenyl acrylate (2g)

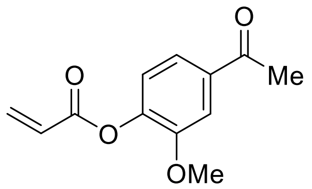

Compound **2g** was prepared according to general procedure **A**, with the use of acrylic acid (75.5 μL, 1.1 mmol) and **apocynin** (166.2 mg, 1 mmol). The crude mixture was purified using flash chromatography (95:5 Hexane/EtOAc) to yield the title compound **2g** (176 mg, 80%) as a colourless oil. **^{1}H NMR** (400 MHz, $CDCl_3$) δ 7.61 (d, *J* = 1.8 Hz, 1H), 7.57 (dd, *J* = 8.2, 2.0 Hz, 1H), 7.17 (d, *J* = 8.1 Hz, 1H), 6.63 (d, *J* = 17.2 Hz, 1H), 6.35 (dd, *J* = 17.4, 10.5 Hz, 1H), 6.05 (d, *J* = 10.5 Hz, 1H), 3.89 (s, 3H), 2.60 (s, 3H). **^{13}C NMR** (101 MHz, $CDCl_3$) δ 196.0, 162.6, 150.5, 142.7, 135.1, 132.3, 126.3, 121.9, 121.0, 110.6, 55.1, 25.6. **Mass** calcd for $C_{12}H_{13}O_4$ [M+H]: 221.0747. Mass Found: 221.0741

(3*S*,8*S*,9*S*,10*R*,13*R*,14*S*,17*R*)-10,13-dimethyl-17-((*R*)-6-methylheptan-2-yl)-2,3,4,7,8,9,10,11,12,13,14,15,16,17-tetradecahydro-1H-cyclopenta[a]phenanthren-3-yl acrylate (2h)

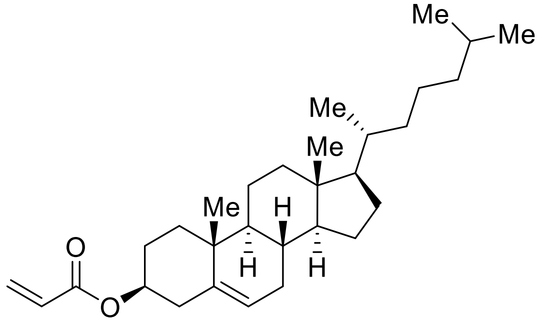

Compound **2h** was prepared according to general procedure **A**, with the use of acrylic acid (75.5 μL, 1.1 mmol) and **cholesterol** (386.7 mg, 1 mmol). The crude mixture was purified using flash chromatography (95:5 Hexane/EtOAc) to yield the title compound **2h** (361 mg, 82%) as a white solid. **^{1}H NMR** (400 MHz, $CDCl_3$) δ 6.36 (dd, *J* = 17.4, 1.6 Hz, 1H), 6.07 (dd, *J* = 17.4, 10.4 Hz, 1H), 5.77 (dd, *J* = 10.5, 1.7 Hz, 1H), 5.36 (d, *J* = 6.2 Hz, 1H), 4.71 – 4.61 (m, 1H), 2.33 (d, *J* = 8.2 Hz, 2H), 2.00 – 1.76 (m, 5H), 1.65 – 1.27 (m, 11H), 1.17 – 0.92 (m, 13H), 0.90 – 0.81 (m, 9H), 0.65 (s, 3H). **^{13}C NMR** (101 MHz, $CDCl_3$) δ 165.8, 139.8, 130.4, 129.2, 122.9, 74.3, 56.8, 56.3, 50.2, 42.5, 39.9, 39.7, 38.2, 37.1, 36.8, 36.3, 35.9, 32.1, 32.0, 28.4, 28.2, 27.9, 24.4, 24.0, 23.0, 22.7, 21.2, 19.5, 18.9, 12.0. Spectral data matches those reported.[12]

(*S*)-4-(2-((tert-butoxycarbonyl)amino)-3-methoxy-3-oxopropyl)phenyl acrylate (2i)

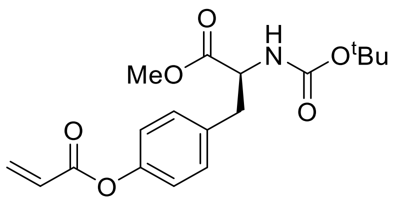

Compound **2i** was prepared according to general procedure **A**, with the use of acrylic acid (75.5 µL, 1.1 mmol) and **Boc-Tyr-OMe** (295.3 mg, 1 mmol). The crude mixture was purified using flash chromatography (80:20 Hexane/EtOAc) to yield the title compound **2h** (314 mg, 90%) as a white solid. ^{1}H NMR (400 MHz, $CDCl_3$) δ 7.14 (d, *J* = 8.4 Hz, 2H), 7.05 (d, *J* = 8.6 Hz, 2H), 6.58 (dd, *J* = 17.3, 1.3 Hz, 1H), 6.29 (dd, *J* = 17.2, 10.4 Hz, 1H), 5.99 (dd, *J* = 10.5, 1.3 Hz, 1H), 5.01 (d, *J* = 8.3 Hz, 1H), 4.59 – 4.54 (m, 1H), 3.69 (s, 3H), 3.13 – 3.01 (m, 2H), 1.41 (s, 9H). **^{13}C NMR** (101 MHz, $CDCl_3$) **^{13}C NMR** (101 MHz, $CDCl_3$) δ 172.3, 164.5, 155.2, 149.7, 133.8, 132.6, 130.4, 128.0, 121.6, 80.1, 54.5, 52.3, 37.8, 28.4. Spectral data matches those reported.[13]

***n*-Butyl 3-(2-(pyridin-2-yl)phenyl)propanoate (3aa)**

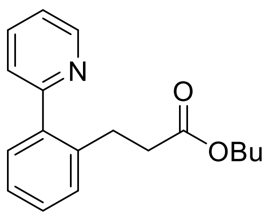

Compound **3aa** was prepared according to general procedure **B**, with the use of 2-phenylpyridine **1a** (64.3 µL, 0.45 mmol) and *n*-butylacrylate **2a** (43.0 µL, 0.3 mmol). The crude mixture was purified using flash chromatography (85:15 Hexane/EtOAc) to yield the title compound **3aa** (76 mg, 89%) as a yellow oil. **^{1}H NMR** (500 MHz, $CDCl_3$) δ 8.61 (d, *J* = 4.4 Hz, 1H), 7.72 – 7.66 (m, 1H), 7.34 (d, *J* = 7.8 Hz, 1H), 7.30 – 7.17 (m, 5H), 3.94 (t, *J* = 6.7 Hz, 2H), 3.01 – 2.95 (m, 2H), 2.49 – 2.43 (m, 2H), 1.51 – 1.45 (m, 2H), 1.29 – 1.19 (m, 2H), 0.84 (t, *J* = 7.4 Hz, 3H). **^{13}C NMR** (126 MHz, $CDCl_3$) δ 173.3, 160.0, 149.3, 140.5, 138.7, 136.5, 130.0, 129.8, 128.6, 126.5, 124.1, 121.9, 64.3, 35.9, 30.7, 28.6, 19.2, 13.8. Spectral data matches those reported.[14]

***n*-Butyl 3-(5-methoxy-2-(pyridin-2-yl)phenyl)propanoate (3ba)**

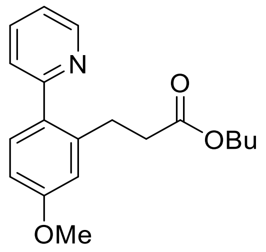

Compound **3ba** was prepared according to general procedure **B**, with the use of 2-(4-methoxyphenyl)pyridine **1b** (83.4 mg, 0.45 mmol) and *n*-butylacrylate **2a** (43.0 µL, 0.3 mmol). The crude mixture was purified using flash chromatography (85:15 Hexane/EtOAc) to yield the title compound **3ba** (59 mg, 63%) as a yellow oil. **^{1}H NMR** (400 MHz, $CDCl_3$) δ 8.58 (d, *J* = 5.6 Hz, 1H), 7.66 (app. t, *J* = 7.7 Hz, 1H), 7.34 – 7.20 (m, 2H), 7.20 – 7.13 (m, 1H), 6.83 – 6.73 (m, 2H), 3.94 (t, *J* = 6.7 Hz, 2H), 3.76 (s, 3H), 3.04 – 2.93 (m, 2H), 2.53 – 2.43 (m, 2H), 1.55 – 1.43 (m, 2H), 1.19 – 1.29 (m, 2H), 0.82 (t, *J* = 7.4 Hz, 3H) . **^{13}C NMR** (126 MHz, $CDCl_3$) δ 173.3, 159.8, 159.7, 149.2, 140.4, 136.4, 133.2, 131.4, 124.0, 121.5, 115.3, 111.8, 64.3, 55.4, 35.8, 30.7, 28.9, 19.2, 13.8. **Mass** calcd for $C_{19}H_{24}O_3N$ [M+H]: 314.1876. Mass Found: 314.1741.

***n*-Butyl 3-(4-(pyridin-2-yl)-[1,1'-biphenyl]-3-yl)propanoate (3ca)**

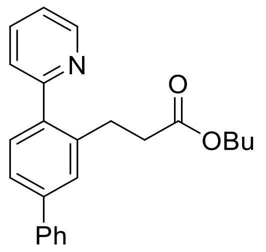

Compound **3ca** was prepared according to general procedure **B**, with the use of 2-([1,1'-biphenyl]-4-yl)pyridine **1c** (104.1 mg, 0.45 mmol) and *n*-butylacrylate **2a** (43.0 µL, 0.3 mmol). The crude mixture was purified using flash chromatography (85:15 Hexane/EtOAc) to yield the title compound **3ca** (56 mg, 52%) as a colorless oil. ^{1}H NMR (400 MHz, $CDCl_3$) δ 8.71 (d, *J* = 4.9 Hz, 1H), 7.80 (app. t, *J* = 7.7 Hz, 1H), 7.63 (d, *J* = 7.1 Hz, 2H), 7.57 – 7.52 (m, 2H), 7.49 – 7.43 (m, 4H), 7.37 (t, *J* = 7.3 Hz, 1H), 7.29 (t, *J* = 7.6 Hz, 1H), 4.02 (t, *J* = 6.7 Hz, 2H), 3.17 – 3.11 (m, 2H), 2.63 – 2.57 (m, 2H), 1.58 – 1.50 (m, 2H), 1.35– 1.26 (m, 2H), 0.88 (t, *J* = 7.4 Hz, 3H). ^{13}C NMR (101 MHz, $CDCl_3$) δ 173.3, 159.5, 149.1, 141.6, 140.8, 139.3, 139.1, 136.8, 130.6, 128.9, 128.8, 127.6, 127.3, 125.3, 124.2, 122.0, 64.4, 35.9, 30.7, 28.8, 19.2, 13.8. **Mass** calcd for $C_{24}H_{25}O_2NNa$ [M+Na]: 382.1778. Mass Found: 382.1775.

***n*-Butyl 3-(5-fluoro-2-(pyridin-2-yl)phenyl)propanoate (3da)**

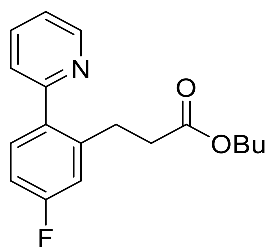

Compound **3da** was prepared according to general procedure **B**, with the use of 2-(4-fluorophenyl)pyridine **1d** (77.9 mg, 0.45 mmol) and *n*-butylacrylate **2a** (43.0 µL, 0.3 mmol). The crude mixture was purified using flash chromatography (85:15 Hexane/EtOAc) to yield the title compound **3da** (81 mg, 90%) as a dark yellow oil. **^{1}H NMR** (500 MHz, $CDCl_3$) δ 8.59 (d, *J* = 4.5 Hz, 1H), 7.69 – 7.66 (m, 1H), 7.29 (d, *J* = 7.8 Hz, 1H), 7.27 – 7.22 (m, 1H), 7.19 (s, 1H), 6.97 – 6.94 (m, 1H), 6.92 – 6.88 (m, 1H), 3.94 (t, *J* = 6.7 Hz, 2H), 2.99 – 2.93 (m, 2H), 2.48 – 2.42 (m, 2H), 1.50 – 1.44 (m, 2H), 1.28 – 1.20 (m, 2H), 0.82 (t, *J* = 7.4 Hz, 3H). **^{13}C NMR** (126 MHz, $CDCl_3$) δ 172.9, 162.7 (d, J = 242.9 Hz), 159.1, 149.3, 141.4 (d, *J* = 7.5 Hz), 136.6(3), 136.6(0), 131.7 (d, *J* = 8.5 Hz), 124.1, 122.0, 116.4 (d, *J* = 21.3 Hz), 113.3 (d, *J* = 21.1 Hz), 64.4, 35.4, 30.7, 28.6, 19.2, 13.8. **^{19}F NMR** (376 MHz, $CDCl_3$) δ -113.8. **Mass** calcd for $C_{18}H_{21}O_2NF$ [M+H]: 302.1551. Mass Found: 302.1540

***n*-Butyl 3-(5-chloro-2-(pyridin-2-yl)phenyl)propanoate (3ea)**

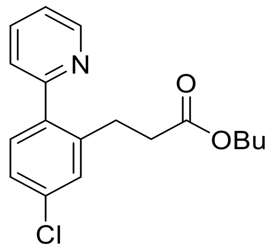

Compound **3ea** was prepared according to general procedure **B**, with the use of 2-(4-chlorophenyl)pyridine **1e** (85.4 mg, 0.45 mmol) and *n*-butylacrylate **2a** (43.0 µL, 0.3 mmol). The crude mixture was purified using flash chromatography (85:15 Hexane/EtOAc) to yield the title compound **3ea** (77 mg, 81%) as a dark yellow oil. **^{1}H NMR** (500 MHz, $CDCl_3$) δ 8.63 (d, *J* = 4.7 Hz, 1H), 7.74 – 7.70 (m, 1H), 7.34 (d, *J* = 7.8 Hz, 1H), 7.28 (d, *J* = 1.7 Hz, 1H), 7.24 (s, 1H), 7.24 – 7.22 (m, 2H), 3.98 (t, *J* = 6.7 Hz, 2H), 3.02 – 2.96 (m, 2H), 2.52 – 2.46 (m, 2H), 1.54 – 1.48 (m, 2H), 1.32 – 1.24 (m, 2H), 0.87 (t, *J* = 7.4 Hz, 3H). **^{13}C NMR** (126 MHz, $CDCl_3$) δ 172.9, 158.9, 149.4, 140.8, 138.9, 136.7, 134.3, 131.4, 129.8, 126.6, 124.0, 122.2, 64.4, 35.5, 30.7, 28.5, 19.2, 13.8. **Mass** calcd for $C_{18}H_{21}O_2NCl$ [M+H]: 318.1255. Mass Found: 318.1248

n-Butyl 3-(2-(pyridin-2-yl)-5-(trifluoromethyl)phenyl)propanoate (3fa)

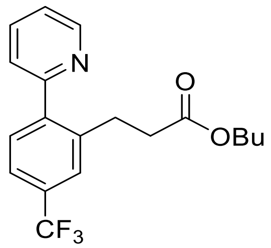

Compound **3fa** was prepared according to general **B**, with the use of 2-(4-(trifluoromethyl)phenyl)pyridine **1f** (67.0 mg, 0.45 mmol) and *n*-butyl acrylate **2a** (43.0 µL, 0.3 mmol). The crude mixture was purified using flash chromatography (85:15 Hexane/EtOAc) to yield the title compound **3fa** (68 mg, 64%) as a yellow oil. **^{1}H NMR** (400 MHz, $CDCl_3$) δ 8.69 (d, J = 5.0 Hz, 1H), 7.83 – 7.77 (m, 1H), 7.59 – 7.53 (m, 2H), 7.46 (d, J = 7.9 Hz, 1H), 7.41 (d, J = 7.8 Hz, 1H), 7.33 – 7.29 (m, 1H), 4.01 (t, J = 6.7 Hz, 2H), 3.12 – 3.05 (m, 2H), 2.58 – 2.51 (m, 2H), 1.58 – 1.49 (m, 2H), 1.35 – 1.26 (m, 2H), 0.89 (t, J = 7.4 Hz, 3H). **^{13}C NMR** (101 MHz, $CDCl_3$) δ 172.8, 158.6, 149.4, 143.8, 139.9, 136.9,130.8, 130.6 (q, J = 33.6 Hz), 126.74 (q, J = 264.0 Hz), 125.5, 123.3 (q, J = 3.7 Hz), 122.8 (q, J = 3.1 Hz), 122.6, 64.5, 35.5, 30.7, 28.6, 19.2, 13.8. ^{19}F NMR (376 MHz, $CDCl_3$) δ -62.4. **Mass** calcd for $C_{19}H_{20}O_2NF_3Na$ [M+Na]: 374.1338. Mass Found: 374.1326

Methyl 3-(3-butoxy-3-oxopropyl)-4-(pyridin-2-yl)benzoate (3ga)

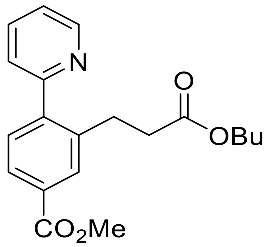

Compound **3ga** was prepared according to general procedure **B**, with the use of methyl 4-(pyridin-2-yl)benzoate **1g** (95.9 mg, 0.45 mmol) and *n*-butyl acrylate **2a** (43.0 µL, 0.3 mmol). The crude mixture was purified using flash chromatography (85:15 Hexane/EtOAc) to yield the title compound **3ga** (52 mg, 51%) as a dark yellow oil. **^{1}H NMR** (500 MHz, $CDCl_3$) δ 8.68 (d, J = 5.9 Hz, 1H), 8.03 – 7.90 (m, 2H), 7.79 – 7.76 (m, 1H), 7.42 (d, J = 7.8 Hz, 2H), 7.28 (s, 1H), 4.00 (t, J = 6.7 Hz, 2H), 3.92 (s, 3H), 3.09 – 3.06 (m, 2H), 2.56 – 2.53 (m, 2H), 1.60 – 1.47 (m, 2H), 1.32 –1.28 (m, 2H), 0.88 (t, J = 7.5 Hz, 3H). **^{13}C NMR** (126 MHz, $CDCl_3$) δ 173.0, 166.9, 158.9, 149.4, 144.7, 139.2, 136.8, 131.0, 130.2, 130.1, 127.7, 124.1, 122.5, 64.4, 52.3, 35.6, 30.7, 28.5, 19.2, 13.8. **Mass** calcd for $C_{20}H_{24}O_4N$ [M+H]: 342.1700. Mass Found: 342.1690

***n*-Butyl 3-(3-methoxy-2-(pyridin-2-yl)phenyl)propanoate (3ha)**

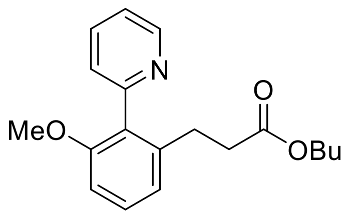

Compound **3ha** was prepared according to general procedure **B**, with the use of 2-(2-methoxyphenyl)pyridine **1h** (77.9 μL, 0.45 mmol) and *n*-butyl acrylate **2a** (43.0 μL, 0.3 mmol). The reaction was stirred at 35 °C for 72 h. The crude mixture was purified using flash chromatography (85:15 Hexane/EtOAc) to yield the title compound **3ha** (58 mg, 62%) as a yellow oil. **^{1}H NMR** (400 MHz, $CDCl_3$) δ 8.73 (d, *J* = 4.4 Hz, 1H), 7.76 (app. t, *J* = 7.6 Hz, 1H), 7.36 – 7.26 (m, 3H), 6.95 (d, *J* = 7.7 Hz, 1H), 6.88 (d, *J* = 8.3 Hz, 1H), 4.01 (t, *J* = 6.6 Hz, 2H), 3.73 (s, 3H), 2.75 (t, *J* = 7.9 Hz, 2H), 2.47 (t, *J* = 7.9 Hz, 2H), 1.59 – 1.52 (m, 2H), 1.37 – 1.29 (m, 2H), 0.92 (t, *J* = 7.4 Hz, 3H). **^{13}C NMR** (126 MHz, $CDCl_3$) δ 173.2, 157.1, 156.5, 149.2, 140.6, 136.2, 129.5, 129.4, 126.0, 122.1, 121.6, 109.1, 64.3, 55.9, 35.5, 30.7, 28.6, 19.2, 13.8. **Mass** calcd for $C_{19}H_{24}O_3N$ [M+H]: 314.1751. Mass Found: 314.1743

***n*-Butyl 3-(3-fluoro-2-(pyridin-2-yl)phenyl)propanoate (3ia)**

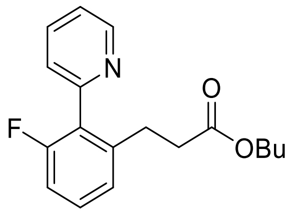

Compound **3ia** was prepared according to general procedure **B**, with the use of 2-(2-fluorophenyl)pyridine **1i** (68.5 μL, 0.45 mmol) and *n*-butyl acrylate **2a** (43.0 μL, 0.3 mmol). The crude mixture was purified using flash chromatography (80:20 Hexane/EtOAc) to yield the title compound **3ia** (28 mg, 31%) as a colorless oil. **^{1}H NMR** (400 MHz, $CDCl_3$) δ 8.72 (d, *J* = 6.1 Hz, 1H), 7.83 – 7.76 (m, 1H), 7.40 (d, *J* = 7.8 Hz, 1H), 7.34 – 7.27 (m, 2H), 7.11 (d, *J* = 7.5 Hz, 1H), 7.02 (t, *J* = 8.9 Hz, 1H), 3.99 (t, *J* = 6.7 Hz, 2H), 2.87 (t, *J* = 7.9 Hz, 2H), 2.47 (t, *J* = 7.9 Hz, 2H), 1.57 – 1.49 (m, 2H), 1.35 – 1.34 (m, 2H), 0.89 (t, *J* = 7.4 Hz, 3H). **^{13}C NMR** (101 MHz, $CDCl_3$) δ 172.9, 158.9 (d, *J* = 247.9 Hz), 153.7, 149.5, 141.7 (d, *J* = 2.6 Hz), 136.5, 129.8 (d, *J* = 9.0 Hz), 128.2, 125.9, 125.1 (d, *J* = 3.4 Hz), 122.6, 113.6 (d, *J* = 22.9 Hz), 64.3, 35.4, 30.7, 28.3, 19.2, 13.8. **^{19}F NMR** (376 MHz, $CDCl_3$) δ -116.2. **Mass** calcd for $C_{18}H_{21}O_2NF$ [M+H]: 302.1551. Mass Found: 302.1548

***n*-Butyl 3-(4-methyl-2-(pyridin-2-yl)phenyl)propanoate (3ja)**

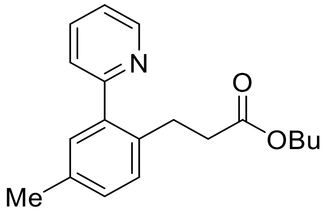

Compound **3ja** was prepared according to general procedure **B**, with the use of 2-(m-tolyl)pyridine **1j** (73.9 µL, 0.45 mmol) and *n*-butyl acrylate **2a** (43.0 µL, 0.3 mmol). The reaction was stirred at 35 °C for 72 h. The crude mixture was purified using flash chromatography (85:15 Hexane/EtOAc) to yield the title compound **3ja** (65 mg, 73%) as a yellow oil. **^{1}H NMR** (400 MHz, $CDCl_3$) δ 8.68 – 8.66 (m, 1H), 7.76 – 7.72 (m, 1H), 7.39 (d, *J* = 7.7 Hz, 1H), 7.25 – 7.12 (m, 4H), 4.00 (t, *J* = 6.7 Hz, 2H), 3.00 (t, *J* = 8.1 Hz, 2H), 2.51 – 2.47 (m, 2H), 2.35 (s, 3H), 1.57 – 1.50 (m, 2H), 1.34 – 1.28 (m, 2H), 0.90 (t, *J* = 7.4 Hz, 3H). **^{13}C NMR** (101 MHz, $CDCl_3$) δ 173.4, 160.1, 149.3, 140.4, 136.4, 136.0, 135.6, 130.7, 129.7, 129.3, 124.1, 121.8, 64.3, 36.0, 30.7, 28.2, 21.1, 19.2, 13.8. **Mass** calcd for $C_{19}H_{24}O_2N$ [M+H]: 298.1802. Mass Found: 298.1801

***n*-Butyl 3-(2-(pyridin-2-yl)-4-(trifluoromethyl)phenyl)propanoate (3ka)**

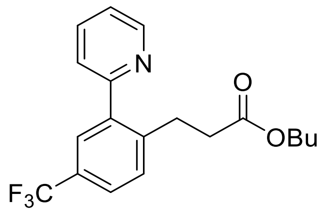

Compound **3ka** was prepared according to general procedure **B**, with the use of 2-(3-(trifluoromethyl)phenyl)pyridine **1k** (81.6 µL, 0.45 mmol) and *n*-butyl acrylate **2a** (43.0 µL, 0.3 mmol). The reaction was stirred at 35 °C for 72 h. The crude mixture was purified using flash chromatography (85:15 Hexane/EtOAc) to yield the title compound **3ka** (43 mg, 41%) as a yellow oil. **^{1}H NMR** (400 MHz, $CDCl_3$) δ 8.70 (d, *J* = 4.0 Hz, 1H), 7.83 – 7.78 (m, 1H), 7.61 – 7.57 (m, 2H), 7.44 (t, *J* = 7.6 Hz, 2H), 7.33 – 7.30 (m, 1H), 4.01 (t, *J* = 6.7 Hz, 2H), 3.09 (t, *J* = 7.8 Hz, 2H), 2.54 (t, *J* = 7.9 Hz, 2H), 1.58 – 1.50 (m, 2H), 1.33 – 1.25 (m, 2H), 0.89 (t, *J* = 7.4 Hz, 3H). **^{13}C NMR** (126 MHz, $CDCl_3$) δ 172.9, 158.6, 149.5, 142.0 (q, J = 243.3 Hz), 136.9, 130.4, 128.9 (q, J = 32.6 Hz), 127.5, 126.9 (q, J = 3.6 Hz), 125.2 (q, J = 3.3 Hz), 124.1, 123.1, 122.6, 64.5, 35.4, 30.7, 28.5, 19.2, 13.8. **^{19}F NMR** (376 MHz, $CDCl_3$) δ -62.4. **Mass** calcd for $C_{19}H_{21}O_2NF_3$ [M+H]: 352.1519. Mass Found: 352.1512

Methyl 4-(3-butoxy-3-oxopropyl)-3-(pyridin-2-yl)benzoate (3la)

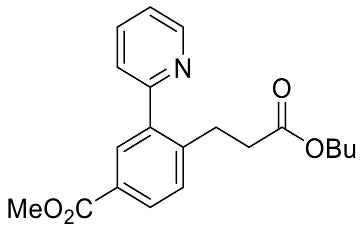

Compound **3la** was prepared according to general procedure **B**, with the use of methyl 3-(pyridin-2-yl)benzoate **1l** (95.9 mg, 0.45 mmol) and *n*-butyl acrylate **2a** (43.0 µL, 0.3 mmol). The crude mixture was purified using flash chromatography (85:15 Hexane/EtOAc) to yield the title compound **3la** (30 mg, 29%) as a yellow oil. **^{1}H NMR** (400 MHz, $CDCl_3$) δ 8.70 (d, *J* = 4.8 Hz, 1H), 8.04 – 7.99 (m, 2H), 7.83 (t, *J* = 7.7 Hz, 1H), 7.47 (d, *J* = 7.8 Hz, 1H), 7.40 (d, *J* = 7.9 Hz, 1H), 7.36 – 7.29 (m, 1H), 4.00 (t, *J* = 6.7 Hz, 2H), 3.90 (s, 3H), 3.10 (t, *J* = 7.8 Hz, 2H), 2.55 (t, *J* = 7.8 Hz, 2H), 1.57 – 1.49 (m, 2H), 1.33 – 1.26 (m, 2H), 0.89 (t, *J* = 7.3 Hz, 3H). **^{13}C NMR** (126 MHz, $CDCl_3$) δ 173.0, 166.9, 159.0, 149.2, 144.3, 140.5, 136.9, 131.3, 130.1, 129.7, 128.5, 124.2, 122.4, 64.4, 52.2, 35.4, 30.7, 28.7, 19.2, 13.8. **Mass** calcd for $C_{20}H_{24}O_4N$ [M+H]: 342.1700. Mass Found: 342.1693

***n*-Butyl 3-(2-(5-methylpyridin-2-yl)phenyl)propanoate (3ma)**

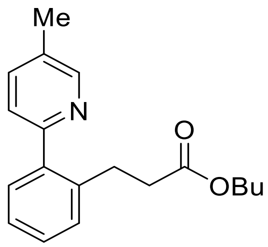

Compound **3ma** was prepared according to general procedure **B**, with the use of 5-methyl-2-phenylpyridine **1m** (76.2 mg, 0.45 mmol) and *n*-butyl acrylate **2a** (43.0 µL, 0.3 mmol). The crude mixture was purified using flash chromatography (85:15 Hexane/EtOAc) to yield the title compound **3ma** (82 mg, 92%) as a yellow oil. **^{1}H NMR** (500 MHz, $CDCl_3$) δ 8.46 (s, 1H), 7.51 (d, *J* = 7.4 Hz, 1H), 7.32 – 7.20 (m, 5H), 3.97 (t, *J* = 6.5 Hz, 2H), 3.01 – 2.98 (m, 2H), 2.49 – 2.46 (m, 2H), 2.34 (s, 3H), 1.53 – 1.47 (m, 2H), 1.31 – 1.24 (m, 2H), 0.86 (t, *J* = 7.3 Hz, 3H). **^{13}C NMR** (126 MHz, $CDCl_3$) δ 173.4, 157.1, 149.7, 140.5, 138.8, 137.1, 131.3, 130.0, 129.8, 128.4, 126.5, 123.5, 64.3, 35.9, 30.7, 28.7, 19.2, 18.3, 13.8. **Mass** calcd for $C_{19}H_{24}O_2N$ [M+H]: 298.1802. Mass Found: 298.1797

***n*-Butyl 3-(2-(1H-pyrazol-1-yl)phenyl)propanoate (3na)**

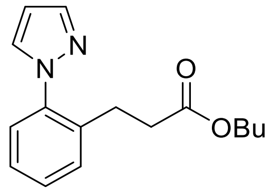

Compound **3na** was prepared according to general procedure **B**, with the use of 1-phenyl-1H-pyrazole **1n** (60.0 µL, 0.45 mmol) and *n*-butyl acrylate **2a** (43.0 µL, 0.3 mmol). The reaction was stirred at 35 °C for 72 h. The crude mixture was purified using flash chromatography (85:15 Hexane/EtOAc) to yield the title compound **3na** (64 mg, 78%) as a dark yellow oil. ^{1}**H NMR** (500 MHz, $CDCl_3$) δ 7.72 (s, 1H), 7.62 (s, 1H), 7.36 – 7.35 (m, 2H), 7.31 – 7.30 (m, 2H), 6.45 (s, 1H), 4.01 (t, *J* = 6.7 Hz, 2H), 2.90 (t, *J* = 7.7 Hz, 2H), 2.45 (t, *J* = 7.6 Hz, 2H), 1.57 – 1.51 (m, 2H), 1.35 – 1.27 (m, 2H), 0.90 (t, *J* = 7.4 Hz, 3H). ^{13}**C NMR** (126 MHz, $CDCl_3$) δ 173.0, 140.6, 139.8, 136.8, 130.8, 130.5, 128.9, 127.3, 126.7, 106.6, 64.4, 34.9, 30.7, 27.0, 19.2, 13.8. **Mass** calcd for $C_{16}H_{21}N_2O_2$ [M+H]: 273.1598. Mass Found: 273.1590

***n*-Butyl 3-(2-(2-oxo-2,3-dihydro-1H-benzo[e][1,4]diazepin-5-yl)phenyl)propanoate (3oa)**

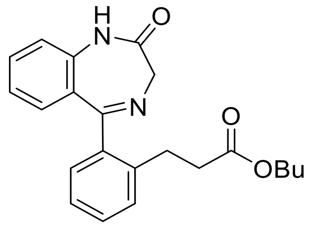

Compound **3oa** was prepared according to general procedure **B**, with the use of $MnBr(CO)_3(MeCN)_2$ (20 mol%, 12.0 mg), 5-phenyl-1,3-dihydro-2H-benzo[e][1,4]diazepin-2-one **1o** (47.2 mg, 0.2 mmol) and *n*-butyl acrylate **2a** (57.3 µL, 0.4 mmol) in Et_2O (0.2 ml). The reaction was stirred at 35 °C for 72 h. The crude mixture was purified using flash chromatography (75:25 Hexane/EtOAc) to yield the title compound **3oa** (39 mg, 53%) as a white solid. ^{1}**H NMR** (400 MHz, $CDCl_3$) δ 7.92 (d, *J* = 4.8 Hz, 1H), 7.30 (t, *J* = 7.8 Hz, 2H), 7.20 (t, *J* = 7.1 Hz, 2H), 7.08 (d, *J* = 4.4 Hz, 1H), 6.98 – 6.87 (m, 3H), 4.10 – 3.97 (m, 2H), 3.74 (t, *J* = 8.1 Hz, 1H), 3.63 (d, *J* = 15.2 Hz, 1H), 3.48 – 3.31 (m, 2H), 3.10 – 3.04 (m, 1H), 1.65 – 1.62 (m, 1H), 1.52 – 1.44 (m, 2H), 1.23 – 1.16 (m, 2H), 0.82 (t, *J* = 7.4 Hz, 3H). ^{13}**C NMR** (126 MHz, $CDCl_3$) δ 172.4, 171.5, 140.9, 136.9, 131.2, 130.1, 128.7, 128.6, 127.1, 126.3, 125.4, 125.2, 124.8, 124.1, 121.9, 74.5, 64.9, 64.8, 33.1, 30.6, 19.1, 13.8; **mp** 186-188 ºC. **Mass** calcd for $C_{22}H_{25}O_3N_2$ [M+H]: 365.1860. Mass Found: 365.1859.

***n*-Butyl 3-(2-(7-chloro-1-methyl-2-oxo-2,3-dihydro-1H-benzo[e][1,4]diazepin-5-yl)phenyl)propanoate (3pa)**

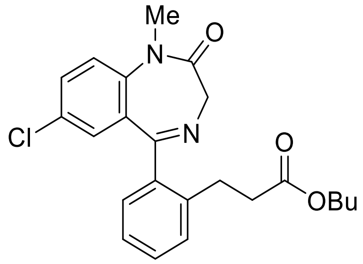

Compound **3pa** was prepared according to general procedure **B**, with the use of $MnBr(CO)_3(MeCN)_2$ (20 mol%, 12.0 mg), 7-chloro-1-methyl-5-phenyl-1,3-dihydro-2H-benzo[e][1,4]diazepin-2-one **1p** (56.9 mg, 0.2 mmol) and *n*-butyl acrylate **2a** (57.3 µL, 0.4 mmol) in Et_2O (0.2 ml). The reaction was stirred at 35 °C for 72 h. The crude mixture was purified using flash chromatography (75:25 Hexane/EtOAc) to yield the title compound **3pa** (64 mg, 77%) as a colourless oil. **^{1}H NMR** (400 MHz, $CDCl_3$) δ 7.43 – 7.36 (m, 1H), 7.34 – 7.22 (m, 3H), 7.22 – 7.17 (m, 1H), 7.14 – 7.11 (m, 1H), 6.41 (d, *J* = 2.0 Hz, 1H), 4.12 – 3.95 (m, 2H), 3.46 (d, *J* = 13.8 Hz, 1H), 3.39 – 3.36 (m, 3H), 3.34 (d, *J* = 4.3 Hz, 1H), 3.29 (d, *J* = 7.9 Hz, 2H), 3.01 – 2.96 (m, 1H), 2.82 – 2.73 (m, 1H), 1.64 – 1.53 (m, 2H), 1.33 – 1.22 (m, 2H), 0.89 – 0.83 (m, 3H). **^{13}C NMR** (126 MHz, $CDCl_3$) δ 173.7, 145.0, 141.8, 141.2, 137.4, 131.5, 130.1, 129.3, 129.2, 129.1, 127.5, 126.5, 126.4, 125.0, 124.4, 74.4, 64.9, 49.1, 35.4, 34.6, 30.6, 19.2, 13.8. **Mass** calcd for $C_{23}H_{26}O_3N_2Cl$ [M+H]: 413.1313. Mass Found: 413.1307

***n*-Butyl 3-(2-fluoro-6-(pyridin-2-yl)phenyl)propanoate (3qa) and *n*-Butyl 3-(4-fluoro-2-(pyridin-2-yl)phenyl)propanoate (3qa')**

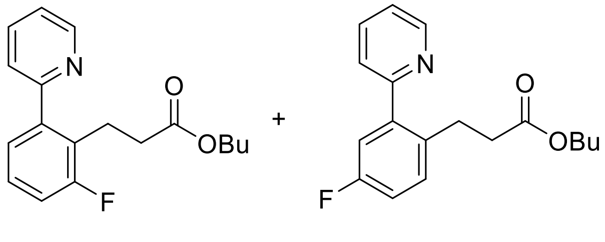

3qa **3qa'**

Compound **3qa and 3qa'** were prepared according to general procedure **B**, with the use of 2-(3-(trifluoromethyl)phenyl)pyridine **1q** (68.5 µL, 0.45 mmol) and *n*-butyl acrylate **2a** (43.0 µL, 0.3 mmol). The reaction was stirred at 35 °C for 24 h. The crude mixture was purified using flash chromatography (85:15 Hexane/Et_2O) to yield a mixture of two regioisomers **3qa/3qa'** in ratio 5.7/1 (81.4 mg, 90%) as a yellow oil. The major isomer was determined to be **3qa**, based on ^{19}F-^{13}C coupling at the -CH_2CH_2CO- motif (no such coupling is observed for **3qa'**). Peaks corresponding to the major compound **3qa**, are as follows: **^{1}H NMR** (400 MHz, $CDCl_3$) δ 8.73 – 8.62 (m, 1H), 7.76 (app. t, *J* = 7.7 Hz, 1H), 7.39 (d, *J* = 7.8 Hz, 1H), 7.30 – 7.26 (m, 1H), 7.23 (d, *J* = 7.7 Hz, 1H), 7.15 (d, *J* = 7.6 Hz, 1 H), 7.08 (t, *J* = 9.0 Hz, 1H),, 4.01 (t, *J* = 6.7 Hz, 2H), 3.07 – 2.97 (m, 2H), 2.65 – 2.55 (m, 2 H), 1.58 – 1.51 (m, 2H), 1.37 – 1.27 (m, 2H), 0.90 (t, *J* = 7.4 Hz, 3H). ^{13}C NMR (101 MHz, $CDCl_3$) δ 173.0, 161.8 (d, *J* = 245.1 Hz), 158.8, 149.3, 142.7, 136.6,

127.6 (d, J = 9.2 Hz), 126.4 (d, J = 16.1 Hz), 125.6 (d, J = 3.0 Hz), 124.0, 122.3, 115.3 (d, J = 23.0 Hz), 64.3, 34.6 (d, J = 1.7 Hz), 30.7, 21.8 (d, J = 3.8 Hz), 19.2, 13.8. **^{19}F NMR** (376 MHz, $CDCl_3$) δ -117.05. **Mass** calcd for $C_{18}H_{20}O_2NFNa$ [M+Na]: 412.1313. Mass Found: 413.1307.

Methyl 3-(2-(pyridin-2-yl)phenyl)propanoate (3ab)

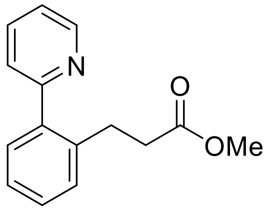

Compound **3ab** was prepared according to general procedure **B**, with the use of 2-phenylpyridine **1a** (64.3 µL, 0.45 mmol) and methylacrylate **2b** (27.2 µL, 0.3 mmol). The crude mixture was purified using flash chromatography (80:20 Hexane/EtOAc) to yield the title compound **3ab** (67 mg, 92%) as a yellow oil. **^{1}H NMR** (500 MHz, $CDCl_3$) δ 8.68 (d, J = 4.6 Hz, 1H), 7.78 – 7.74 (m, 1H), 7.41 (d, J = 7.7 Hz, 1H), 7.36 – 7.28 (m, 5H), 3.60 (s, 3H), 3.04 (t, J = 7.9 Hz, 2H), 2.55 (t, J = 7.9 Hz, 2H). **^{13}C NMR** (126 MHz, $CDCl_3$) δ 173.9, 160.1, 149.4, 140.7, 138.9, 136.9, 130.3, 130.1, 128.9, 126.8, 124.4, 122.3, 51.9, 35.9, 28.8. Spectral data matches those reported.[15]

***t*-Butyl 3-(2-(pyridin-2-yl)phenyl)propanoate (3ac)**

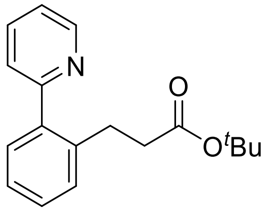

Compound **3ac** was prepared according to general procedure **B**, with the use of 2-phenylpyridine **1a** (64.3 µL, 0.45 mmol) and *t*-butyl acrylate **2c** (43.9 µL, 0.3 mmol). The crude mixture was purified using flash chromatography (85:15 Hexane/EtOAc) to yield the title compound **3ac** (71 mg, 83%) as a yellow oil. **^{1}H NMR** (500 MHz, $CDCl_3$) δ 8.59 (d, J = 5.5 Hz, 1H), 7.65 (app. t, J = 7.7 Hz, 1H), 7.31 (d, J = 7.8 Hz, 1H), 7.24 (d, J = 10.4 Hz, 3H), 7.21 – 7.13 (m, 2H), 2.91 (t, J = 7.9 Hz, 2H), 2.33 (t, J = 7.9 Hz, 2H), 1.28 (s, 9H). **^{13}C NMR** (126 MHz, $CDCl_3$) δ 172.6, 160.0, 149.3, 140.5, 138.9, 136.5, 130.0, 129.8, 128.5, 126.4, 124.1, 121.9, 80.2, 36.9, 28.7, 28.2. Spectral data matches those reported.[14]

4-(2-(pyridin-2-yl)phenyl)butan-2-one (3ad)

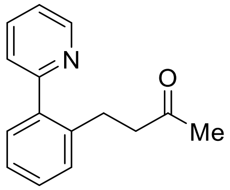

Compound **3ad** was prepared according to general procedure **B**, with the use of 2-phenylpyridine **1a** (64.3 µL, 0.45 mmol) and but-3-en-2-one **2d** (25.0 µL, 0.3 mmol). The reaction was stirred at 35 °C for 72 h. The crude mixture was purified using flash chromatography (85:15 Hexane/EtOAc) to yield the title compound **3ad** (38 mg, 57%) as a yellow oil.^{1}**H NMR** (400 MHz, $CDCl_3$) δ 8.57 (d, J = 4.0 Hz, 1H), 7.67 (app. t, J = 7.7 Hz, 1H), 7.31 (d, J = 7.8 Hz, 1H), 7.26 – 7.15 (m, 5H), 2.89 – 2.83 (m, 2H), 2.59 (t, J = 7.8 Hz, 2H), 1.95 (s, 3H). ^{13}**C NMR** (101 MHz, $CDCl_3$) δ 208.5, 160.1, 149.2, 140.5, 139.2, 136.6, 130.0, 130.0, 128.7, 126.4, 124.2, 122.0, 45.6, 30.0, 27.6. Spectral data matches those reported.[16]

3-(2-(Pyridin-2-yl)phenyl)cyclohexanone (3ae)

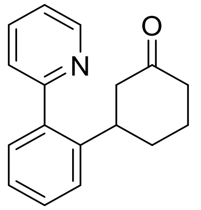

Compound **3ae** was prepared according to general procedure **B**, with the use of 2-phenylpyridine **1a** (64.3 µL, 0.45 mmol) and cyclohex-2-en-1-one **2e** (29.2 µL, 0.3 mmol). The crude mixture was purified using flash chromatography (80:20 Hexane/EtOAc) to yield the title compound **3ae** (35 mg, 46%) as a dark yellow oil. ^{1}**H NMR** (400 MHz, $CDCl_3$) δ 8.59 (d, J = 6.1 Hz, 1H), 7.69 (app. t, J = 8.6 Hz, 1H), 7.37 – 7.32 (m, 2H), 7.29 – 7.17 (m, 4H), 3.27 – 3.17 (m, 1H), 2.45 (d, J = 8.9 Hz, 2H), 2.29 – 2.25 (m, 2H), 2.01 – 1.94 (m, 2H), 1.80 – 1.70 (m, 1H), 1.55 – 1.45 (m, 1H). ^{13}**C NMR** (101 MHz, $CDCl_3$) δ 211.3, 159.7, 149.2, 142.3, 139.9, 136.7, 130.3, 129.0, 126.5, 126.2, 124.3, 122.1, 49.0, 41.3, 40.2, 32.9, 25.6. Spectral data matches those reported.[14]

1,3-diphenyl-3-(2-(pyridin-2-yl)phenyl)propan-1-one (3af)

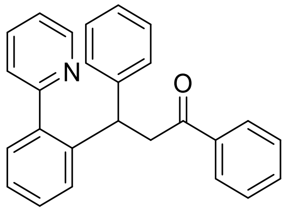

Compound **3af** was prepared according to general procedure **B**, with the use of 2-phenylpyridine **1a** (64.3 µL, 0.45 mmol) and chalcone **2f** (62.5 mg, 0.3 mmol). The reaction was stirred at 35 °C for 72 h. The crude mixture was purified using flash chromatography (80:20 Hexane/EtOAc) to yield the title compound **3af** (100 mg, 92%) as a colorless oil. **^{1}H NMR** (400 MHz, $CDCl_3$) δ 8.61 (d, *J* = 5.3 Hz, 1H), 7.85 – 7.83 (m, 2H), 7.66 (app. t, *J* = 7.7 Hz, 1H), 7.48 (t, *J* = 7.4 Hz, 1H), 7.36 (t, *J* = 7.7 Hz, 2H), 7.33 – 7.27 (m, 4H), 7.25 – 7.20 (m, 2H), 7.12 (t, *J* = 7.2 Hz, 2H), 7.07 – 7.01 (m, 3H), 5.15 (t, *J* = 7.4 Hz, 1H), 3.72 (d, *J* = 7.3 Hz, 2H). **^{13}C NMR** (101 MHz, $CDCl_3$) δ 198.0, 159.8, 148.8, 143.8, 142.4, 140.2, 137.0, 136.7, 133.0, 130.2, 128.8, 128.6, 128.4, 128.2, 128.0, 127.9, 126.5, 126.2, 124.7, 122.1, 45.1, 41.6. Spectral data matches those reported.[17]

4-acetyl-2-methoxyphenyl 3-(2-(pyridin-2-yl)phenyl)propanoate (3ag)

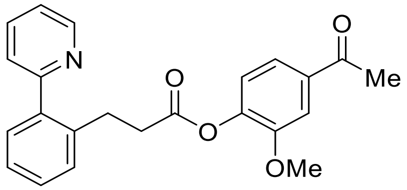

Compound **3ag** was prepared according to general procedure **B**, with the use of 2-phenylpyridine **1a** (64.3 µL, 0.45 mmol) and 4-acetyl-2-methoxyphenyl acrylate **2g** (66.1 mg, 0.3 mmol). The reaction was stirred at 35 °C for 72h. The crude mixture was purified using flash chromatography (85:15 Hexane/EtOAc) to yield the title compound **3ag** (37 mg, 33%) as a colorless oil. **^{1}H NMR** (400 MHz, $CDCl_3$) δ 8.75 – 8.74 (m, 1H), 7.87 (app. t, *J* = 6.7 Hz, 1H), 7.55 (s, 1H), 7.54 – 7.48 (m, 2H), 7.43 – 7.34 (m, 5H), 7.02 (d, *J* = 8.1 Hz, 1H), 3.81 (s, 3H), 3.18 (t, *J* = 7.6 Hz, 2H), 2.88 (t, *J* = 7.6 Hz, 2H), 2.58 (s, 3H). **^{13}C NMR** (126 MHz, $CDCl_3$) δ 197.1, 170.7, 158.7, 151.4, 147.7, 144.0, 139.8, 138.4, 136.0, 130.3, 130.0, 129.4, 126.9, 126.7, 125.0, 122.8, 122.6, 122.1, 111.5, 56.1, 35.2, 28.4, 26.7. **Mass** calcd for $C_{23}H_{22}O_4N$ [M+H]: 376.1543. Mass Found: 376.1542.

(3*S*,8*S*,9*S*,10*R*,13*R*,14*S*,17*R*)-10,13-dimethyl-17-((*R*)-6-methylheptan-2-yl)-2,3,4,7,8,9,10,11,12,13,14,15,16,17-tetradecahydro-1H-cyclopenta[a]phenanthren-3-yl 3-(2-(pyridin-2-yl)phenyl)propanoate (3ah)

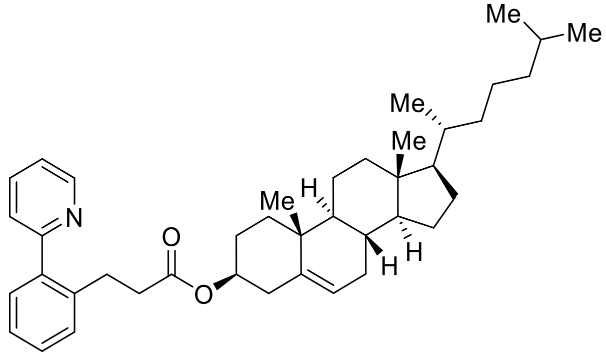

Compound **3ah** was prepared according to general procedure **B**, with the use of 2-phenylpyridine **1a** (64.3 µL, 0.45 mmol) and (3*S*,8*S*,9*S*,10*R*,13*R*,14*S*,17*R*)-10,13-dimethyl-17-((*R*)-6-methylheptan-2-yl)-2,3,4,7,8,9,10,11,12,13,14,15,16,17-tetradecahydro-1H-cyclopenta[a]phenanthren-3-yl acrylate **2h** (132.2 mg, 0.3 mmol). The reaction was stirred at 35 °C for 72h. The crude mixture was purified using flash chromatography (85:15 Hexane/EtOAc) to yield the title compound **3ah** (156 mg, 87%) as a white solid. **^{1}H NMR** (400 MHz, $CDCl_3$) δ 8.63 (d, *J* = 4.9 Hz, 1H), 7.71 (app. t, *J* = 7.7 Hz, 1H), 7.36 (d, *J* = 7.8 Hz, 1H), 7.32 – 7.27 (m, 3H), 7.24 – 7.18 (m, 2H), 5.30 (d, *J* = 5.5 Hz, 1H), 4.55 – 4.45 (m, 1H), 3.01 – 2.97 (m, 2H), 2.45 (t, *J* = 7.9 Hz, 2H), 2.19 (d, *J* = 7.5 Hz, 2H), 1.99 – 1.87 (m, 2H), 1.81 – 1.70 (m, 3H), 1.63 (s, 1H), 1.56 – 1.36 (m, 9H), 1.32 – 1.25 (m, 3H), 1.12 – 1.00 (m, 7H), 0.98 – 0.93 (m, 4H), 0.87 – 0.86 (m, 4H), 0.83 – 0.81 (m, 5H), 0.62 (s, 3H). **^{13}C NMR** (101 MHz, $CDCl_3$) δ 172.6, 160.1, 149.3, 140.5, 139.8, 138.8, 136.5, 130.0, 129.9, 128.6, 126.5, 124.1, 122.7, 121.9, 74.0, 56.8, 56.3, 50.1, 42.4, 39.9, 39.7, 38.2, 37.1, 36.7, 36.3, 36.1, 35.9, 32.0, 32.0, 28.6, 28.4, 28.2, 27.9, 24.4, 24.0, 23.0, 22.7, 21.2, 19.4, 18.8, 12.0; **mp** 82-84 ºC. **Mass** calcd for $C_{41}H_{58}O_2N$ [M+H]: 596.4462. Mass Found: 596.4465

Methyl(*S*)-2-((tert-butoxycarbonyl)amino)-3-(4-((3-(2-(pyridin-2 yl)phenyl)propanoyl)oxy)phenyl)propanoate (3ai)

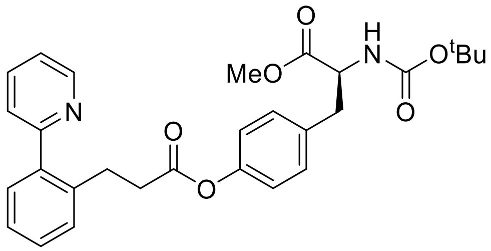

Compound **3ai** was prepared according to general procedure **B**, with the use of 2-phenylpyridine **1a** (64.3 µL, 0.45 mmol) and **2i** (104.8 mg, 0.3 mmol). The reaction was stirred at 35 °C for 72h. The crude mixture was purified using flash chromatography (80:20 Hexane/EtOAc) to yield the title compound **3ai** (134 mg, 88%) as a colorless oil. ^{1}H NMR (400 MHz, $CDCl_3$) δ 8.81 – 8.58 (m, 1H), 7.78 app. (t, *J* = 7.5 Hz, 1H), 7.44 (d, *J* = 7.6 Hz, 1H), 7.40 – 7.27 (m, 5H), 7.09 (d, *J* = 8.2 Hz, 2H), 6.91 (d, *J* = 8.2 Hz, 2H), 5.00 (d, *J* = 8.4 Hz, 1H), 4.58 – 4.53 (m, 1H), 3.69 (s, 3H),

3.16 (t, *J* = 7.6 Hz, 2H), 3.07 – 3.00 (m, 2H), 2.79 (t, *J* = 7.3 Hz, 2H), 1.41 (s, 9H).^{13}C NMR (101 MHz, $CDCl_3$) δ 172.3, 171.6, 159.7, 155.1, 149.8, 148.9, 140.2, 138.4, 136.9, 133.6, 130.2, 130.1, 130.0, 128.8, 126.7, 124.2, 122.1, 121.6, 80.0, 54.4, 52.3, 37.7, 35.8, 29.7, 28.6, 28.3. **Mass** calcd for $C_{29}H_{32}O_6N_2Na$ [M+Na]: 527.215 Mass Found: 527.2154.

(*E*)-2-(2-(hex-1-en-1-yl)phenyl)pyridine (5aa)

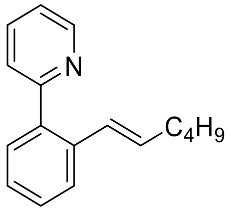

Compound **5aa** was prepared according to general procedure **B**, with the use of 2-phenylpyridine **1a** (64.3 µL, 0.45 mmol) and hexyne **4a** (33.0 µL, 0.3 mmol). The crude mixture was purified using flash chromatography (90:10 Hexane/EtOAc) to yield the title compound **5aa** (36 mg, 50%) as a light yellow oil. **^{1}H NMR** (400 MHz, $CDCl_3$) δ 8.63 (d, *J* = 7.9 Hz, 1H), 7.64 (app. t, *J* = 7.6 Hz, 1H), 7.48 (d, *J* = 8.1 Hz, 1H), 7.40 – 7.33 (m, 2H), 7.28 – 7.14 (m, 3H), 6.35 (d, *J* = 15.2 Hz, 1H), 6.08 – 6.03 (m, 1H), 2.05 (d, *J* = 7.2 Hz, 2H), 1.36 – 1.19 (m, 4H), 0.80 (t, *J* = 7.3 Hz, 3H). **^{13}C NMR** (101 MHz, $CDCl_3$) δ 159.1, 149.3, 138.6, 136.4, 136.1, 132.9, 130.1, 128.6, 128.4, 127.0, 126.4, 125.2, 121.8, 32.9, 31.5, 22.3, 14.0. Spectral data matches those reported.[18]

(*E*)-2-(2-(dec-1-en-1-yl)phenyl)pyridine (5ab)

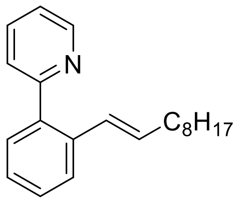

Compound **5ab** was prepared according to general procedure **B**, with the use of 2-phenylpyridine **1a** (64.3 µL, 0.45 mmol) and hexyne **4b** (56.0 µL, 0.3 mmol). The reaction was stirred at 35 °C for 72 h. The crude mixture was purified using flash chromatography (88:12 Hexane/EtOAc) to yield the title compound **5ab** (48 mg, 54%) as a yellow oil. **^{1}H NMR** (400 MHz, $CDCl_3$) δ 8.65 – 8.64 (m, 1H), 7.67 – 7.63 (m, 1H), 7.52 – 7.49 (m, 1H), 7.42 – 7.39 (m, 1H), 7.36 (d, *J* = 7.8 Hz, 1H), 7.30 – 7.26 (m, 1H), 7.25 – 7.21 (m, 1H), 7.19 – 7.16 (m, 1H), 6.37 (d, *J* = 15.8 Hz, 1H), 6.12 – 6.05(m, 1H), 2.10 – 2.02 (m, 2H), 1.38 – 1.30 (m, 2H), 1.24 – 1.14 (m, 10H), 0.83 – 0.77 (m, 3H). **^{13}C NMR** (101 MHz, $CDCl_3$) δ 159.2, 149.4, 138.7, 136.4, 136.0, 133.0, 130.1, 128.6, 128.4, 127.0, 126.4, 125.1, 121.8, 33.3, 32.0, 29.6, 29.4, 29.4, 29.3, 22.8, 14.2. **Mass** calcd for $C_{21}H_{28}N$ [M+H]: 294.2216. Mass Found: 294.2210

(*E*)-2-(2-(4-methoxystyryl)phenyl)pyridine (5ac)

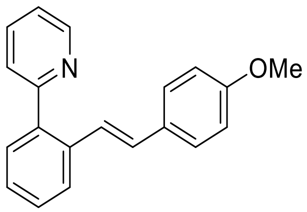

Compound **5ac** was prepared according to general procedure **B**, with the use of 2-phenylpyridine **1a** (64.3 µL, 0.45 mmol) and 1-ethynyl-4-methoxybenzene **4c** (39.3 µL, 0.3 mmol). The crude mixture was purified using flash chromatography (85:15 Hexane/EtOAc) to yield the title compound **5ac** (74 mg, 86%) as a yellow oil. **^{1}H NMR** (400 MHz, $CDCl_3$) δ 8.66 (d, *J* = 5.1 Hz, 1H), 7.65 (d, *J* = 7.9 Hz, 2H), 7.46 (d, *J* = 7.8 Hz, 1H), 7.40 – 7.21 (m, 5H), 7.20 – 7.16 (m, 1H), 7.02 (d, *J* = 16.1 Hz, 1H), 6.92 (d, *J* = 16.3 Hz, 1H), 6.76 (d, *J* = 8.7 Hz, 2H), 3.71 (s, 3H). **^{13}C NMR** (101 MHz, $CDCl_3$) δ 159.3, 159.0, 149.5, 139.4, 136.0, 130.5, 130.3, 129.6, 128.7, 127.8, 127.4, 126.1, 125.4, 125.1, 121.9, 114.1, 55.3. Spectral data matches those reported.[19]

(*E*)-2-(2-styrylphenyl)pyridine (5ad)

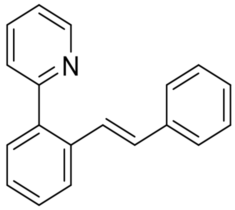

Compound **5ad** was prepared according to general procedure **B**, with the use of 2-phenylpyridine **1a** (64.3 µL, 0.45 mmol) and phenylacetylene **4d** (32.9 µL, 0.3 mmol). The crude mixture was purified using flash chromatography (85:15 Hexane/EtOAc) to yield the title compound **5ad** (65 mg, 84%) as a light yellow oil. **^{1}H NMR** (500 MHz, $CDCl_3$) δ 8.65 (d, *J* = 4.5 Hz, 1H), 7.66 – 7.63 (m, 2H), 7.45 (d, *J* = 7.4 Hz, 1H), 7.37 – 7.33 (m, 1H), 7.32 (d, *J* = 7.5 Hz, 1H), 7.28 (d, *J* = 8.0 Hz, 3H), 7.21 – 7.17 (m, 3H), 7.14 – 7.09 (m, 2H), 6.95 (d, *J* = 16.2 Hz, 1H). **^{13}C NMR** (126 MHz, $CDCl_3$) δ 158.8, 149.5, 139.4, 137.7, 136.4, 135.9, 130.4, 130.3, 128.9, 128.8, 127.9, 127.7, 127.6, 126.7, 126.4, 125.3, 122.1. Spectral data matches those reported.[19]

(*E*)-2-(2-(4-bromostyryl)phenyl)pyridine (5ae)

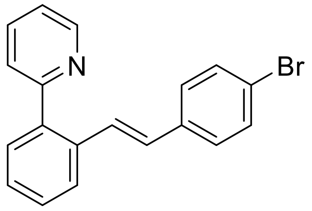

Compound **5ae** was prepared according to general procedure **B**, with the use of 2-phenylpyridine **1a** (64.3 µL, 0.45 mmol) and 1-bromo-4-ethynylbenzene **4e** (54.3 µL, 0.3 mmol). The crude mixture was purified using flash chromatography (85:15 Hexane/EtOAc) to yield the title compound **5ae** (93 mg, 92%) as a yellow oil. **^{1}H NMR** (400 MHz, $CDCl_3$) δ 8.64 (d, *J* = 4.9 Hz, 1H), 7.66 – 7.62 (m, 2H), 7.44 (d, *J* = 8.4 Hz, 1H), 7.36 – 7.27 (m, 5H), 7.19 – 7.16 (m, 1H), 7.15 – 7.08 (m, 3H), 6.87 (d, *J* = 16.3 Hz, 1H). **^{13}C NMR** (101 MHz, $CDCl_3$) δ 158.9, 149.5, 139.6, 136.6, 136.3, 135.5, 131.8, 130.4, 128.9, 128.4, 128.2, 128.0, 126.4, 125.1, 122.1, 121.4. Spectral data matches those reported.[19]

(*E*)-methyl 4-(2-(pyridin-2-yl)styryl)benzoate (5af)

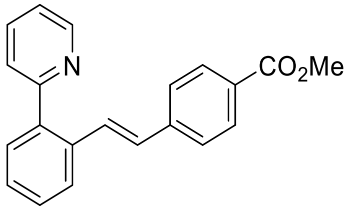

Compound **5af** was prepared according to general procedure **B**, with the use of 2-phenylpyridine **1a** (64.3 µL, 0.45 mmol) and methyl 4-ethynylbenzoate **4f** (48.0 mg, 0.3 mmol). The crude mixture was purified using flash chromatography (80:20 Hexane/EtOAc) to yield the title compound **5ae** (37 mg, 39%) as a yellow oil. **^{1}H NMR** (400 MHz, $CDCl_3$) δ 8.79 (d, *J* = 4.5 Hz, 1H), 7.97 (d, *J* = 8.1 Hz, 2H), 7.86 – 7.75 (m, 2H), 7.60 (d, *J* = 7.5 Hz, 1H), 7.53 – 7.41 (m, 5H), 7.38 – 7.31 (m, 2H), 7.08 (d, *J* = 16.1 Hz, 1H), 3.90 (s, 3H). **^{13}C NMR**. (101 MHz, $CDCl_3$) δ 167.0, 158.9, 149.7, 142.2, 140.0, 136.3, 135.3, 130.4, 130.3, 130.1, 129.0, 128.9, 128.8, 128.4, 126.5, 126.5, 125.1, 122.1, 52.2. Spectral data matches those reported.[19]

(*E*)-2-(2-(2-(naphthalen-2-yl)vinyl)phenyl)pyridine (5ag)

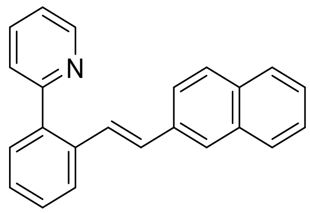

Compound **5ag** was prepared according to general procedure **B**, with the use of 2-phenylpyridine **1a** (64.3 µL, 0.45 mmol) and 2-ethynylnaphthalene **4g** (45.6 mg, 0.3 mmol). The crude mixture was purified using flash chromatography (80:20 Hexane/EtOAc) to yield the title compound **5ag** (85 mg, 92%) as a white solid.**^{1}H NMR** (400 MHz, $CDCl_3$) δ 8.68 (d, *J* = 5.4 Hz, 1H), 7.74 – 7.62 (m, 6H), 7.48 (app.t, *J* = 8.6 Hz, 2H), 7.40 – 7.29 (m, 6H), 7.20 – 7.17 (m, 1H), 7.13 (d, *J* = 16.3 Hz, 1H). **^{13}C NMR** (101 MHz, $CDCl_3$) δ 158.9, 149.6, 139.7, 136.2, 135.8, 135.2, 133.7, 133.0, 130.4, 130.3, 128.8, 128.3, 128.1, 128.0, 127.8, 127.8, 126.8, 126.4, 126.3, 126.0, 125.2, 123.7, 122.0. Spectral data matches those reported.[19]

(*E*)-2-(2-(3-phenylprop-1-en-1-yl)phenyl)pyridine (5ah)

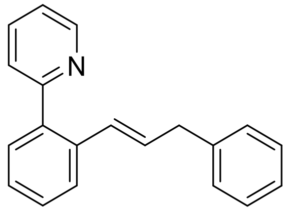

Compound **5ah** was prepared according to general procedure **B**, with the use of 2-phenylpyridine **1a** (64.3 µL, 0.45 mmol) and prop-2-yn-1-ylbenzene **4h** (38.0 µL, 0.3 mmol). The reaction was stirred at 35 °C for 72 h. The crude mixture was purified using flash chromatography (85:15 Hexane/EtOAc) to yield the title compound **5ah** (52 mg, 64%) as a light yellow oil. **^{1}H NMR** (400 MHz, $CDCl_3$) δ 8.61(d, *J* = 4.9 Hz, 1H), 7.61 (app.t, *J* = 8.6 Hz, 1H), 7.48 (d, *J* = 7.3 Hz, 1H), 7.39 – 7.37 (m, 1H), 7.31 (d, *J* = 7.8 Hz, 1H), 7.25 – 7.12 (m, 5H), 7.09 (d, *J* = 7.2 Hz, 3H), 6.43 (d, *J* = 15.6 Hz, 1H), 6.23 – 6.15 (m, 1H), 3.38 (d, *J* = 6.8 Hz, 2H). **^{13}C NMR** (101 MHz, $CDCl_3$) δ 159.0, 149.4, 140.3, 138.9, 136.1, 136.0, 130.8, 130.1, 129.9, 128.8, 128.7, 128.6, 127.3, 126.5, 126.2, 125.1, 121.9, 39.6. Spectral data matches those reported.[20]

(*E*)-2-(2-(4-Phenylbut-1-en-1-yl)phenyl)pyridine (5ai)

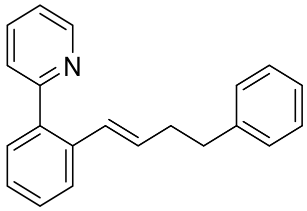

Compound **5ai** was prepared according to general procedure **B**, with the use of 2-phenylpyridine **1a** (64.3 µL, 0.45 mmol) and but-3-yn-1-ylbenzene **4i** (41.7 µL, 0.3 mmol). The reaction was stirred at 35 °C for 72 h. The crude mixture was purified using flash chromatography (85:15 Hexane/EtOAc) to yield the title compound **5ai** (53 mg, 62%) as a yellow oil. **^{1}H NMR** (400 MHz, $CDCl_3$) δ 8.64 (d, J = 4.7 Hz, 1H), 7.62 – 7.56 (m, 1H), 7.51 (d, J = 7.5 Hz, 1H), 7.42 (d, J = 7.2 Hz, 1H), 7.33 – 7.25 (m, 2H), 7.22 – 7.11 (m, 7H), 6.39 (d, J = 15.7 Hz, 1H), 6.18 – 6.10 (m, 1H), 2.70 (t, J = 7.5 Hz, 2H), 2.43 (q, J = 7.1 Hz, 2H). **^{13}C NMR** (126 MHz, $CDCl_3$) δ 158.4, 148.8, 141.8, 137.9, 136.7, 136.2, 131.9, 130.2, 129.1, 129.0, 128.7, 128.4, 127.3, 126.6, 125.9, 125.5, 122.0, 35.7, 35.1. Spectral data matches those reported.[21]

Methyl (*E*)-(4-(2-(pyridin-2-yl)styryl)benzoyl)-L-alaninate (5aj)

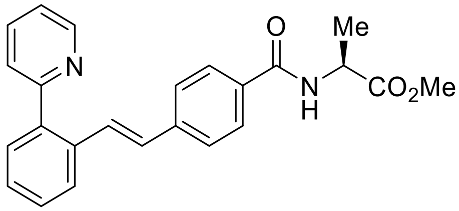

Compound **5aj** was prepared according to general procedure **B**, with the use of 2-phenylpyridine **1a** (64.3 µL, 0.45 mmol) and methyl (4-ethynylbenzoyl)-L-alaninate **4j** (69.4 mg, 0.3 mmol). The reaction was stirred at 35 °C for 72 h. The crude mixture was purified using flash chromatography (70:30 Hexane/EtOAc) to yield the title compound **5aj** (102 mg, 88%) as a yellow oil. **^{1}H NMR** (400 MHz, $CDCl_3$) δ 8.68 (d, J = 4.2 Hz, 1H), 7.74 – 7.62 (m, 4H), 7.49 – 7.47 (m, 1H), 7.40 – 7.32 (m, 5H), 7.25 – 7.16 (m, 2H), 6.97 (d, J = 16.3 Hz, 1H), 6.74 (d, J = 7.3 Hz, 1H), 4.73 – 4.66 (m, 1H), 3.69 (s, 3H), 1.42 (d, J = 7.2 Hz, 3H). **^{13}C NMR** (101 MHz, $CDCl_3$) δ 173.8, 166.4, 158.4, 149.1, 141.0, 139.2, 136.9, 135.4, 132.6, 130.5, 129.6, 129.1, 129.1, 128.3, 127.6, 126.7, 126.5, 125.3, 122.3, 52.7, 48.6, 18.7. Spectral data matches those reported.[22]

(*E*)-2-(2-(1-phenylprop-1-en-1-yl)phenyl)pyridine (5ak)

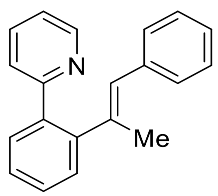

Compound **5ak** was prepared according to general procedure **C**, with the use of 2-phenylpyridine **1a** (43.0 µL, 0.3 mmol) and prop-1-yn-1-ylbenzene **4k** (55.6 µL, 0.45 mmol). The crude mixture was purified using flash chromatography (85:15 Hexane/EtOAc) to yield the title compound **5ak** (46 mg, 57%) as a colorless oil. **^{1}H NMR** (400 MHz, $CDCl_3$) δ 8.71 (d, *J* = 5.6 Hz, 1H), 7.69 – 7.61 (m, 2H), 7.54 (d, *J* = 7.8 Hz, 1H), 7.43 – 7.42 (m, 3H), 7.33 (d, *J* = 6.8 Hz, 2H), 7.24 – 7.19 (m, 4H), 6.47 (s, 1H), 1.93 (s, 3H). **^{13}C NMR** (101 MHz, $CDCl_3$) δ 159.7, 149.6, 144.8, 139.1, 138.8, 138.2, 135.9, 130.8, 130.3, 129.1, 128.9, 128.5, 128.3, 127.5, 126.5, 124.3, 121.7, 20.2. **Mass** calcd for $C_{20}H_{18}N$ [M+H]: 272.1434. Mass Found: 272.1425. Structure confirmed using **HMBC** and **2-D NOESY NMR**.

(*E*)-2-(2-(1-phenylpent-1-en-2-yl)phenyl)pyridine (5al)

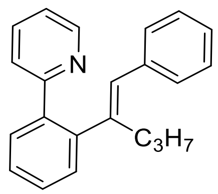

Compound **5al** was prepared according to general procedure **C**, with the use of 2-phenylpyridine **1a** (43.0 µL, 0.3 mmol) and pent-1-yn-1-ylbenzene **4l** (71.0 µL, 0.45 mmol). The crude mixture was purified using flash chromatography (85:15 Hexane/EtOAc) to yield the title compound **5al** (61 mg, 68%) as a colorless oil. **^{1}H NMR** (400 MHz, $CDCl_3$) δ 8.60 (d, *J* = 4.9 Hz, 1H), 7.56 – 7.49 (m, 3H), 7.32 – 7.29 (m, 3H), 7.23 (d, *J* = 7.5 Hz, 2H), 7.14 – 7.09 (m, 4H), 6.45 (s, 1H), 2.04 – 1.96 (m, 2H), 1.17 – 1.08 (m, 2H), 0.59 (t, *J* = 7.3 Hz, 3H). **^{13}C NMR** (101 MHz, $CDCl_3$) δ 159.6, 149.6, 144.7, 143.0, 138.8, 138.2, 135.8, 130.8, 130.2, 130.0, 128.8, 128.4, 128.3, 127.5, 126.6, 124.4, 121.8, 34.1, 21.8, 14.1. **Mass** calcd for $C_{22}H_{22}N$ [M+H]: 300.1747. Mass Found: 300.1744. Structure confirmed using **HMBC** and **2-D NOESY NMR**.

Methyl (*Z*)-3-phenyl-2-(2-(pyridin-2-yl)phenyl)acrylate (5am)

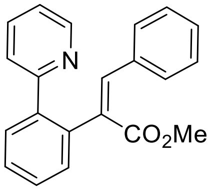

Compound **5am** was prepared according to general procedure **C**, with the use of 2-phenylpyridine **1a** (43.0 µL, 0.3 mmol) and methyl 3-phenylpropiolate **4m** (64.4 µL, 0.45 mmol). The crude mixture was purified using flash chromatography (80:20 Hexane/EtOAc) to yield the title compound **5am** (44 mg, 47%) as a colorless oil. ^{1}**H NMR** (400 MHz, $CDCl_3$) δ 8.56 (d, *J* = 4.9 Hz, 1H), 7.72 (d, *J* = 6.0 Hz, 2H), 7.59 – 7.55 (m, 1H), 7.51 – 7.47 (m, 1H), 7.40 – 7.36 (m, 1H), 7.34 (d, *J* = 7.9 Hz, 1H), 7.24 – 7.22 (m, 1H), 7.20 – 7.10 (m, 4H), 7.03 – 7.00 (m, 2H), 3.57 (s, 3H).^{13}**C NMR** (126 MHz, $CDCl_3$) δ 168.4, 158.6, 158.4, 149.4, 140.5, 140.3, 136.1, 134.8, 134.8, 132.9, 131.1, 130.5, 130.0, 129.1, 129.0, 128.7, 128.4, 123.1, 121.8, 121.6, 52.3. **Mass** calcd for $C_{21}H_{18}O_2N$ [M+H]: 316.1332. Mass Found: 316.1326. Structure confirmed using **HMBC** and **2-D NOESY NMR**.

(*E*)-2-(2-(1,2-diphenylvinyl)phenyl)pyridine (5an)

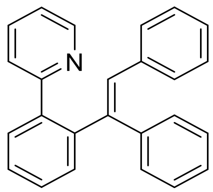

Compound **5an** was prepared according to general procedure **C**, with the use of 2-phenylpyridine **1a** (43.0 µL, 0.3 mmol) and 1,2-diphenylethyne **4n** (80.2 µL, 0.45 mmol). The crude mixture was purified using flash chromatography (80:20 Hexane/EtOAc) to yield the title compound **5an** (47 mg, 47%) as a colorless oil. ^{1}**H NMR** (500 MHz, $CDCl_3$) δ 8.46 (d, *J* = 4.9 Hz, 1H), 7.52 – 7.51 (m, 1H), 7.46 – 7.40(m, 4H), 7.32 (d, *J* = 7.8 Hz, 1H), 7.13 – 7.08 (m, 3H), 7.05 – 7.01 (m, 4H), 7.00 – 6.98 (m, 2H), 6.93 – 6.92 (m, 2H), 6.67 (s, 1H). ^{13}**C NMR** (126 MHz, $CDCl_3$) δ 159.7, 149.1, 143.6, 142.5, 140.6, 140.3, 137.7, 135.5, 131.2, 131.0, 130.3, 129.3, 128.3, 128.0, 127.8, 127.8, 126.9, 126.8, 124.5, 121.3. Spectral data matches those reported.[23] Structure confirmed using **2-D NOESY NMR**.

(*E*)-2-(2-(1,2-bis(4-bromophenyl)vinyl)phenyl)pyridine (5ao)

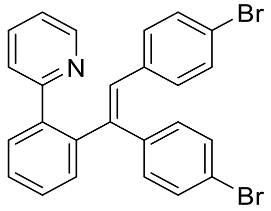

Compound **5ao** was prepared according to general procedure **C**, with the use of 2-phenylpyridine **1a** (43.0 µL, 0.3 mmol) and 1,2-bis(4-bromophenyl)ethyne **4o** (151.2 µL, 0.45 mmol). The crude mixture was purified using flash chromatography (80:20 Hexane/EtOAc) to yield the title compound **5ao** (74 mg, 50%) as a colorless oil. **^{1}H NMR** (400 MHz, $CDCl_3$) δ 8.53 (d, *J* = 4.0 Hz, 1H), 7.49 (app. t, *J* = 4.7 Hz, 4H), 7.30 (d, *J* = 9.5 Hz, 4H), 7.15 (d, *J* = 8.4 Hz, 3H), 6.94 (d, *J* = 8.6 Hz, 2H), 6.77 (d, *J* = 8.6 Hz, 2H), 6.69 (s, 1H). **^{13}C NMR** (126 MHz, $CDCl_3$) δ 159.4, 148.8, 142.9, 142.2, 140.2, 139.0, 136.2, 136.0, 131.9, 131.3, 131.1, 131.0, 130.9, 130.4, 130.2, 128.6, 128.3, 124.4, 121.6, 121.2, 121.0. **Mass** calcd for $C_{25}H_{18}NBr_2$ [M+H]: 489.9801. Mass Found: 489.9799. Structure confirmed using **2-D NOESY NMR**.

7. Mechanistic Studies

7.1 Competition experiments between electron-rich and poor aromatics

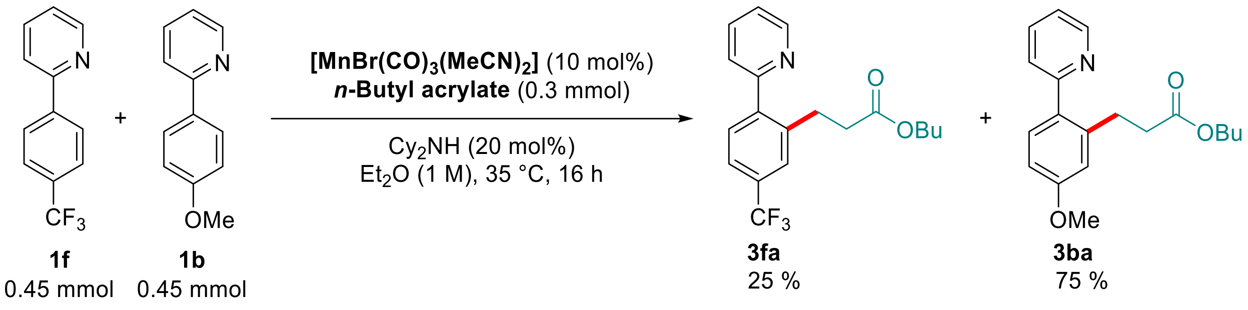

In an argon-filled glove box, $[MnBr(CO)_3(MeCN)_2]$ (9.0 mg, 10 mol%) catalyst was weighed and transferred to an oven dried microwave vial containing magnetic stirrer bar, followed by addition of **1f** (100.4 mg, 0.45 mmol), **1b** (83.25 mg,0.45 mmol), *n*-butyl acrylate (43.2 µL, 0.3 mmol), and Cy_2NH (11.9 µL, 20 mol%) dissolved in diethyl ether (0.3 mL, 1 M).The vial was sealed, taken out of the glove box and the reaction stirred at 35 °C for 16 h. After this time, 1 mL of a stock solution with internal standard (1,3,5- trimethoxybenzene (0.1 M in Et_2O)) was added to the reaction. The reaction was then filtered through a short pad of silica into an NMR tube. Analysis of the crude using **^{1}H NMR**, with reference to the spectra of pure compounds, showed the formation of **3fa** (25%) and **3ba** (75%).

7.2 Kinetic Concentration Sensitivity Experiments

General procedure employing 2-phenylpyridine **1a** and butylacrylate **2a**:

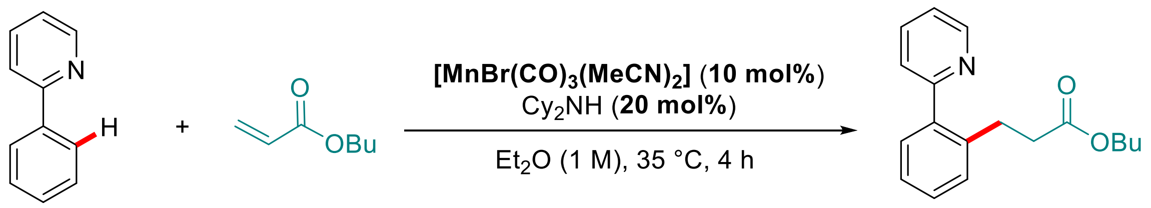

In an argon-filled glove box, $MnBr(CO)_3(MeCN)_2$ catalyst was weighed and transferred to an oven dried microwave vial containing a magnetic stirrer bar, followed by addition of 2-phenylpyridine **1a**. Stock solutions of *n*-butyl acrylate **2a** and Cy_2NH in Et_2O were prepared, these were added to the vial via microsyringe. The vial was sealed, taken out of the glove box and the reaction stirred at 35 °C for 4 h. After the time duration, a stock solution of internal standard hexadecane was added and the reaction mixture was filtered through a short plug of silica into a GC vial ready for analysis.

Entry	Variation	Recovered 1a (mmol)	3aa (mmol)	kinetic sensitivity
1	-	0.34	0.10	-
2	**1a** (0.9 mmol)	0.70	0.19	Positive on **1a**
3	**2a** (0.9 mmol)	0.37	0.06	Negative on **2a**
4	**3aa** (0.1 mmol)	0.36	0.18	Negative on **3aa**
5	[Mn] (5 mol%)	0.38	0.07	Positive on [Mn]
6	Cy_2NH (10 mol%)	0.37	0.08	Positive on Cy_2NH

Table 6. Results of kinetic order. Yields determined by GC-FID using hexadecane as an internal standard.

7.3 Kinetic Experiments for determination of orders

7.3.1 General procedure for kinetic experiments employing 2-phenylpyridine **1a** and butylacrylate **2a**:

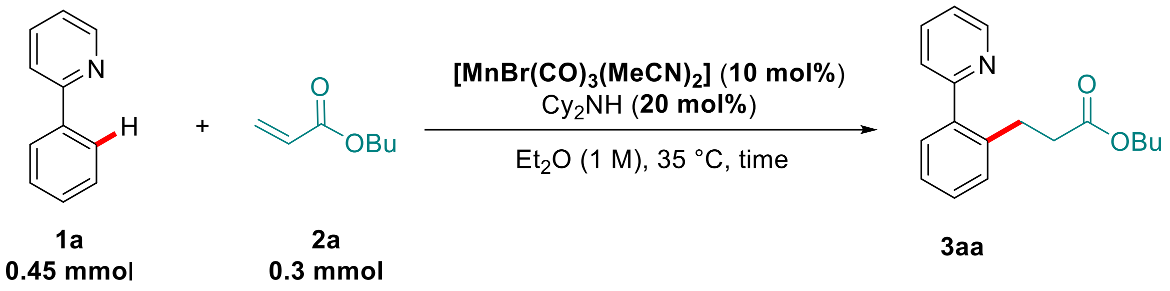

In an argon-filled glove box, $MnBr(CO)_3(MeCN)_2$ catalyst was weighed and transferred to an oven dried microwave vial containing a magnetic stirrer bar. Stock solutions in Et_2O were prepared for *n*-butyl acrylate **2a** and Cy_2NH, and internal standard hexadecane, these were added to the vial. The vial was capped with a rubber stopper and the reaction was then heated at 35 °C inside the glove box, before a solution of 2-phenylpyridine **1a** in Et_2O was added at 0 min to start the reaction. Aliquots of approximately 20 µL were then taken throughout the first 4 h of the reaction at specified time points. Each aliquot was added to approximately 0.5 mL of a solution of 1% pyridine in EtOAc (v/v), before being passed through a short plug of silica into a GC vial ready for analysis. The reaction was then monitored by GC-FID, using hexadecane as the internal standard.

7.3.2 Determination of Order in Catalyst

The order in catalyst has been determined using normalized time scale analysis. Reactions were carried out with different concentrations of catalyst and their temporal profiles were normalized according to the catalyst loading raised to the power of the order in the catalyst. All the resulting curves were plotted together and the correct order in catalyst is the one that causes the curves to overlay.

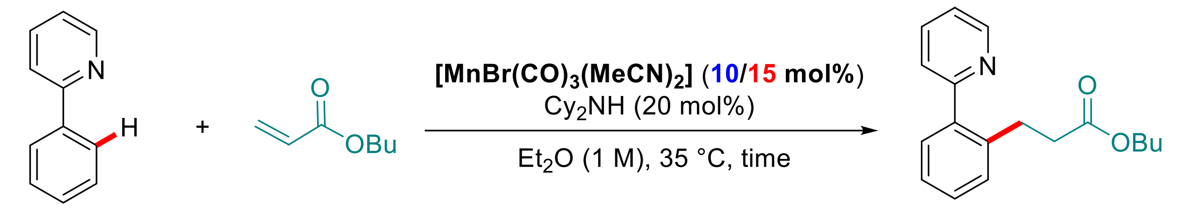

1a
2.25 mmol

2a
1.5 mmol

3aa

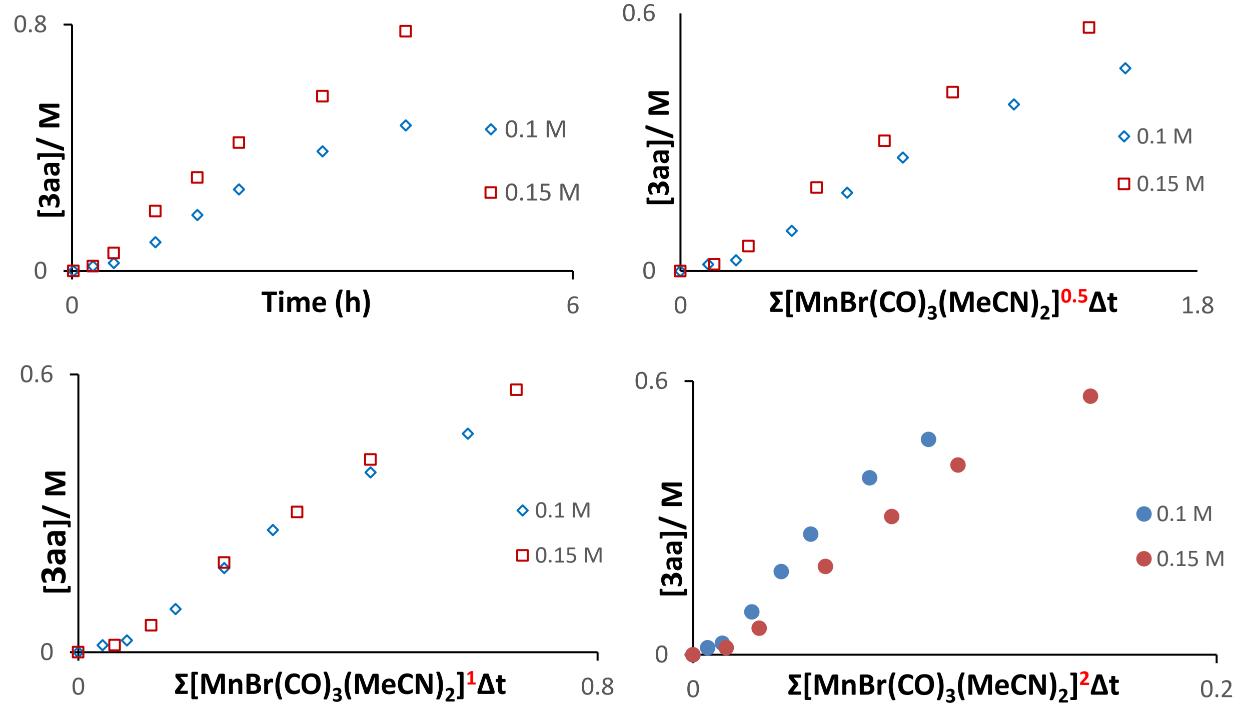

Figure 1. Determination of order in catalyst. (a) Temporal reaction profiles of reactions carried out with 10/15 mol % of $[MnBr(CO)_3(MeCN)_2]$; (b) Normalized time scale profiles for order 0.5 in $[MnBr(CO)_3(MeCN)_2]$; (c) Normalized time scale profiles for order 1.0 in $[MnBr(CO)_3(MeCN)_2]$; (d) Normalized time scale profiles for order 2.0 in $[MnBr(CO)_3(MeCN)_2]$.

The overlap between the temporal reaction profiles with catalyst loadings of 10 and 15 mol % suggests that the order in $[MnBr(CO)_3(MeCN)_2]$ is 1.0 at these concentrations.

7.3.3 Determination of Orders in Additive

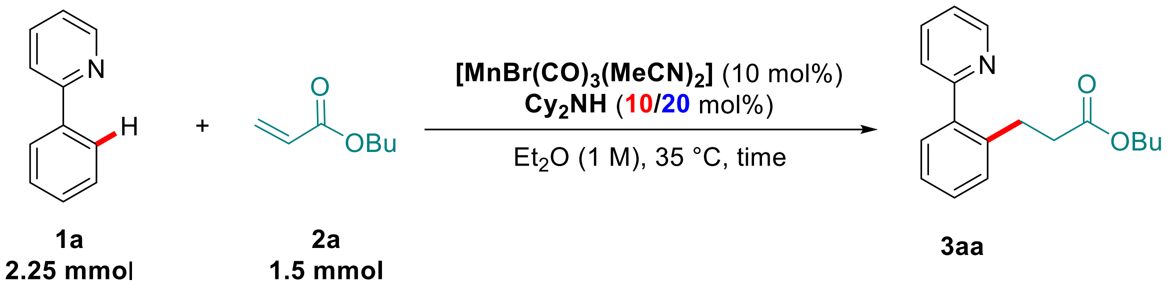

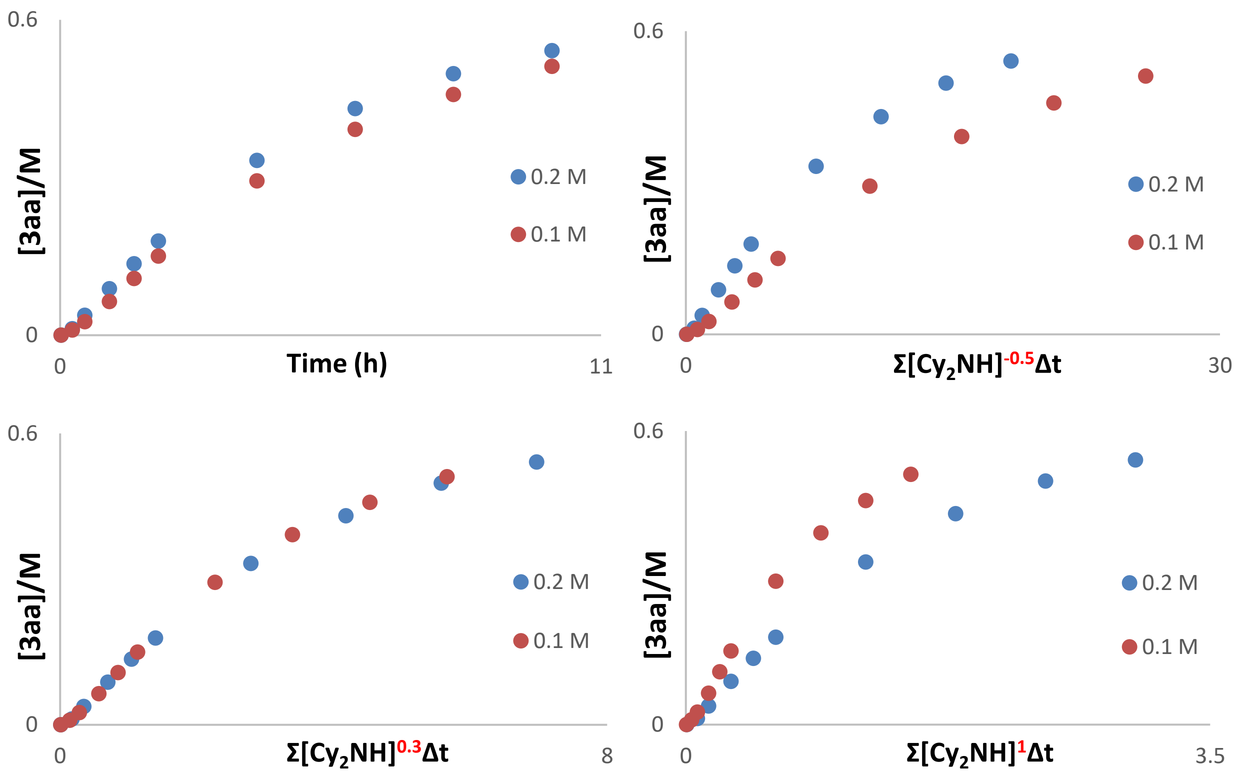

Figure 2. Determination of order in Cy_2NH. (a) Temporal reaction profiles of reactions carried out with 10/20 mol % of [Cy_2NH]; (b) Normalized time scale profiles for order -0.5 in [Cy_2NH]; (c) Normalized time scale profiles for order 0.3 in [Cy_2NH]; (d) Normalized time scale profiles for order 1 in [Cy_2NH].

The overlap between the temporal reaction profiles with 10 and 20 mol % of additive suggests that the order in Cy_2NH is 0.3 at these concentrations.

7.3.4 Determination of Orders in Reagents

Determination of order in 1a

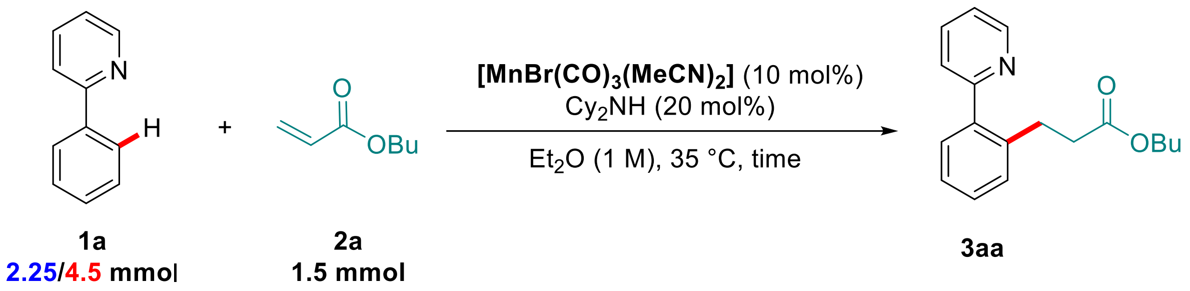

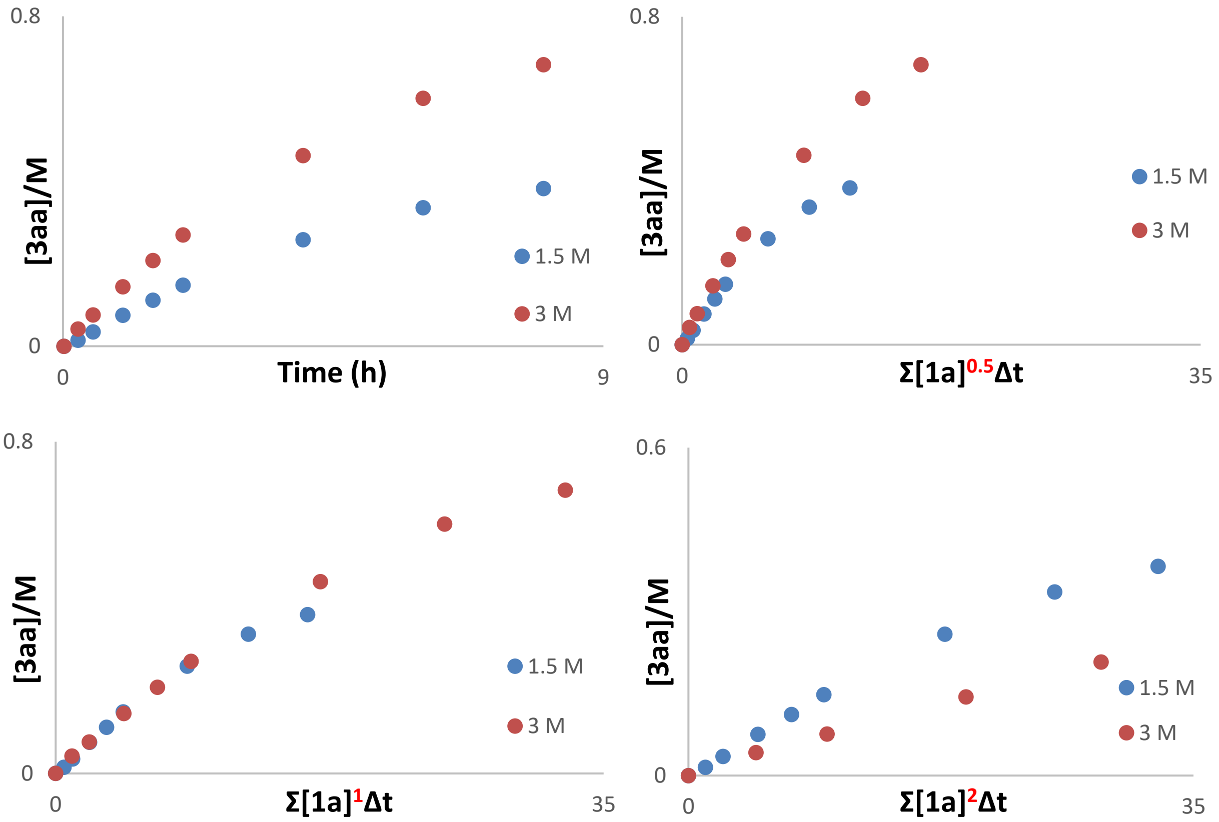

Figure 3. Determination of order in 1a. (a) Temporal reaction profiles of reactions carried out with 2.25/4.5 mmol of [1a]; (b) Normalized time scale profiles for order 0.5 in [1a]; (c) Normalized time scale profiles for order 1 in [1a]; (d) Normalized time scale profiles for order 2 in [1a].

The overlap between normalised time scale reaction profiles for these two reactions with differing concentrations of 1a shows an order of 1. This strongly suggests the C-H activation step of 1a is kinetically relevant.

Determination of order in 2a

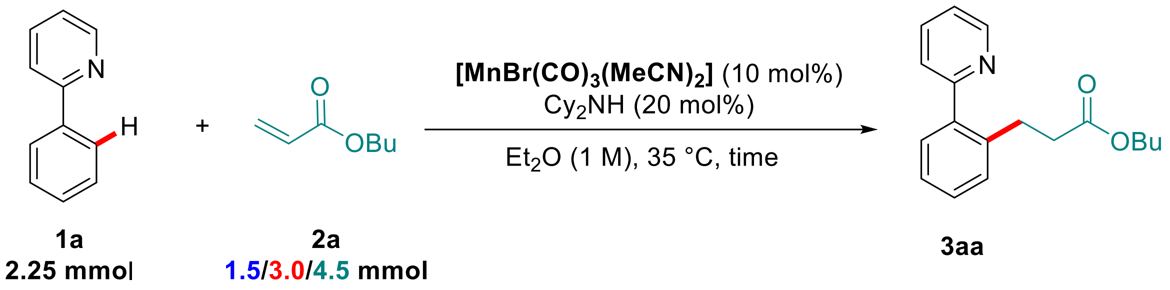

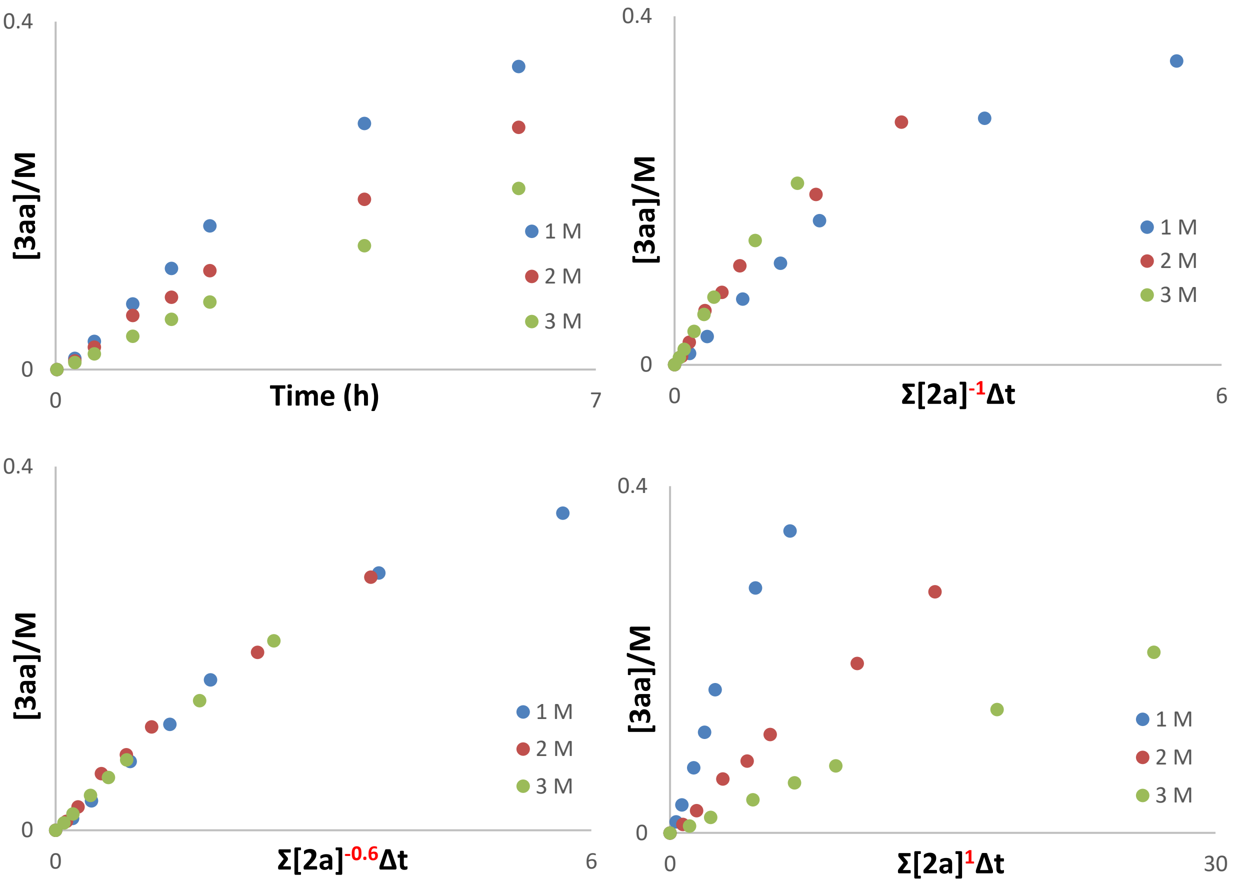

Figure 4. Determination of order in 2a. (a) Temporal reaction profiles of reactions carried out with 1.5/3/4.5 mmol of [2a]; (b) Normalized time scale profiles for order -1 in [2a]; (c) Normalized time scale profiles for order -0.6 in [2a]; (d) Normalized time scale profiles for order 1 in [2a].

The overlap between reaction profiles for these two reactions with differing concentrations of 2a shows an order of -0.6. This inverse dependence on the concentration of alkene suggests that multiple coordination of the alkene are possible to form an off-cycle species of the type [Mn(2a)$_2$].

8. Copies of ^{1}H and ^{13}C NMR for isolated Compounds

[MnBr(CO)3(MeCN)2] complex

^{1}H-NMR (500 MHz, DMSO-d6)

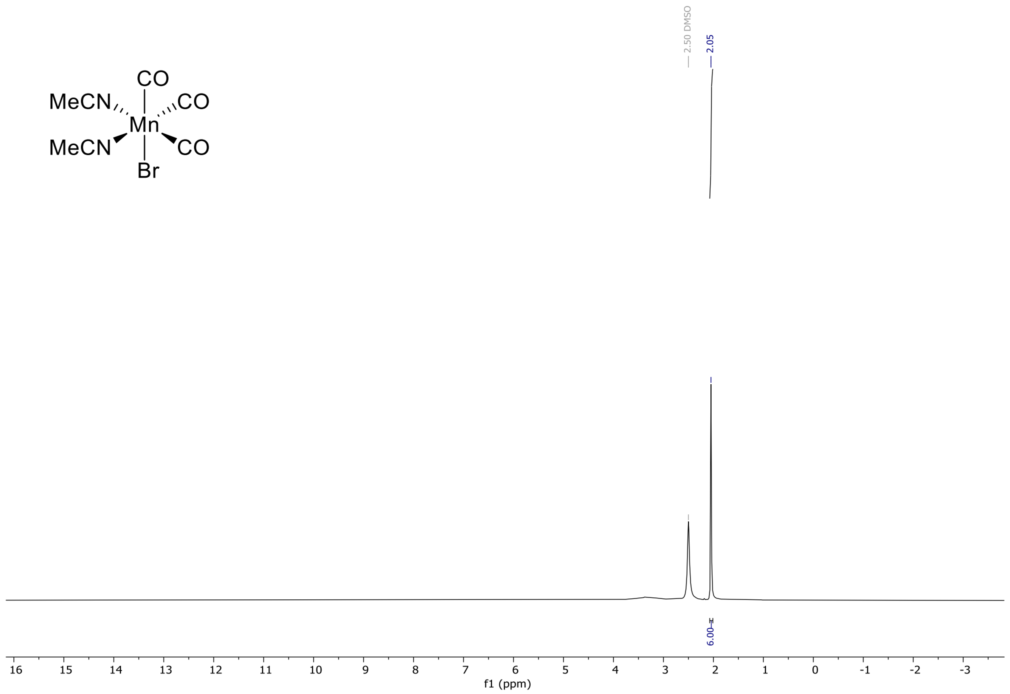

^{13}C-NMR (126 MHz, DMSO-d6)

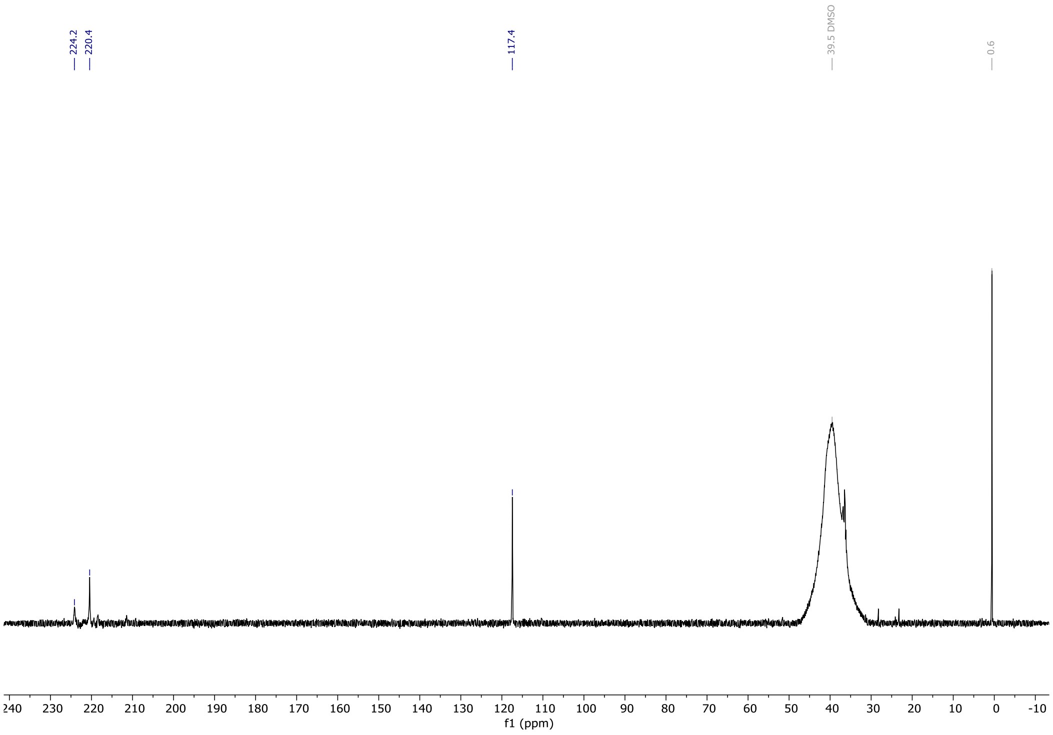

4-acetyl-2-methoxyphenyl acrylate (2g)

^{1}H-NMR (400 MHz, $CDCl_3$)

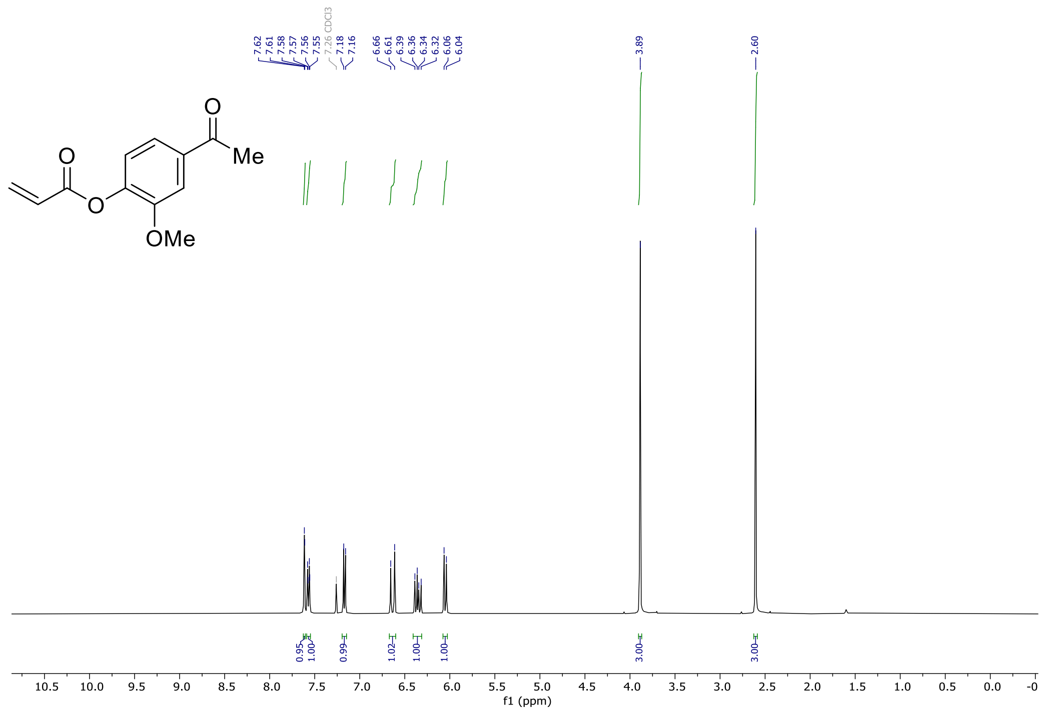

^{13}C-NMR (101 MHz, $CDCl_3$)

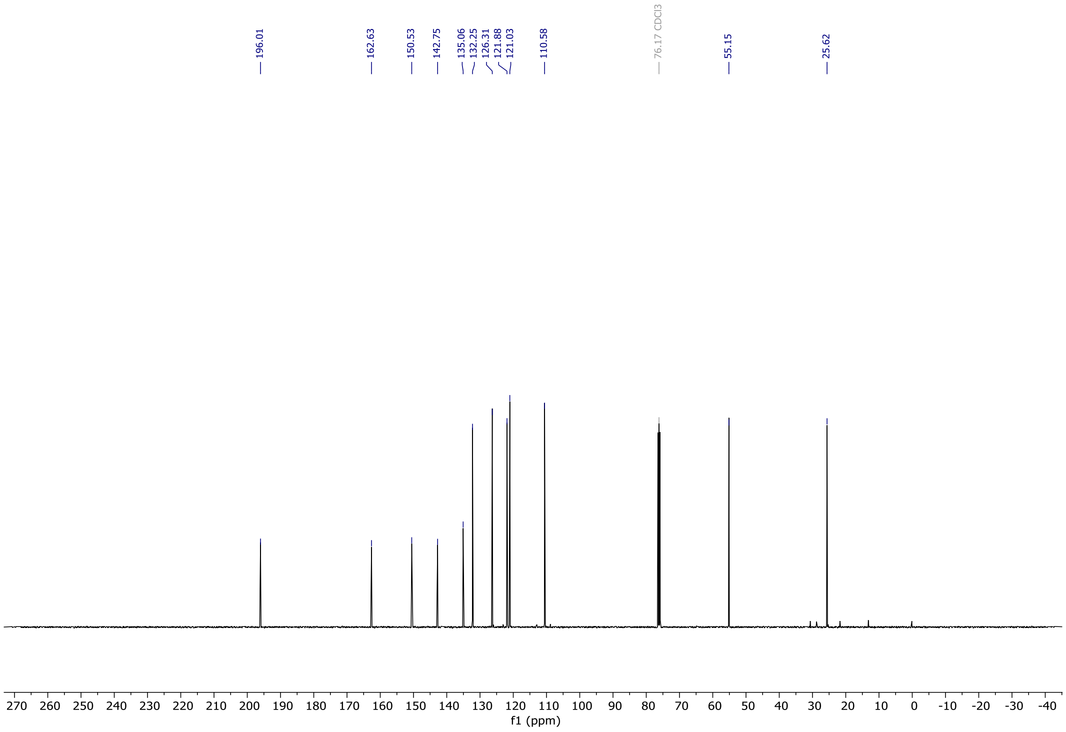

(3*S*,8*S*,9*S*,10*R*,13*R*,14*S*,17*R*)-10,13-dimethyl-17-((*R*)-6-methylheptan-2-yl)-2,3,4,7,8,9,10,11,12,13,14,15,16,17-tetradecahydro-1H-cyclopenta[a]phenanthren-3-yl acrylate (2h)

^{1}H-NMR (400 MHz, $CDCl_3$)

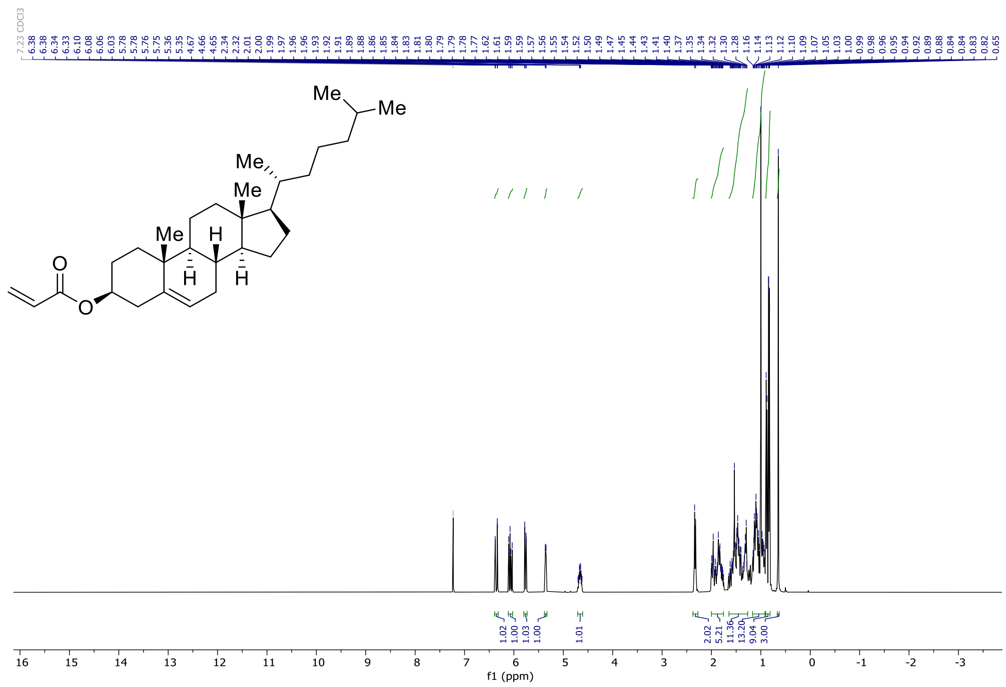

^{13}C-NMR (101 MHz, $CDCl_3$)

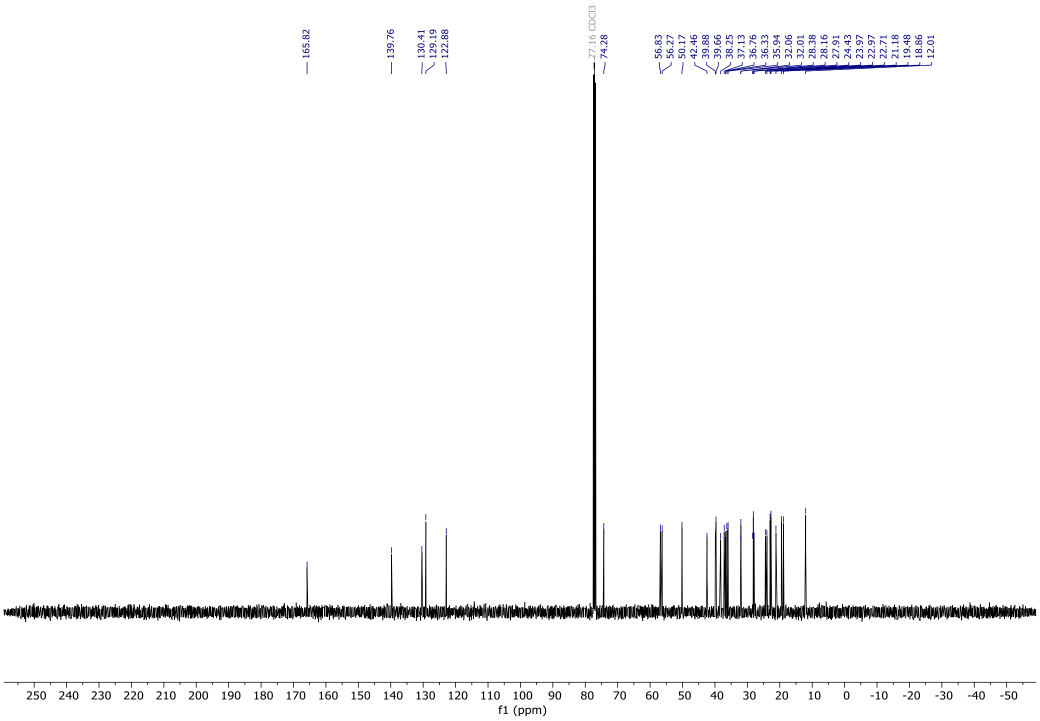

(*S*)-4-(2-((tert-butoxycarbonyl)amino)-3-methoxy-3-oxopropyl)phenyl acrylate (2i)

^{1}H-NMR (400 MHz, $CDCl_3$)

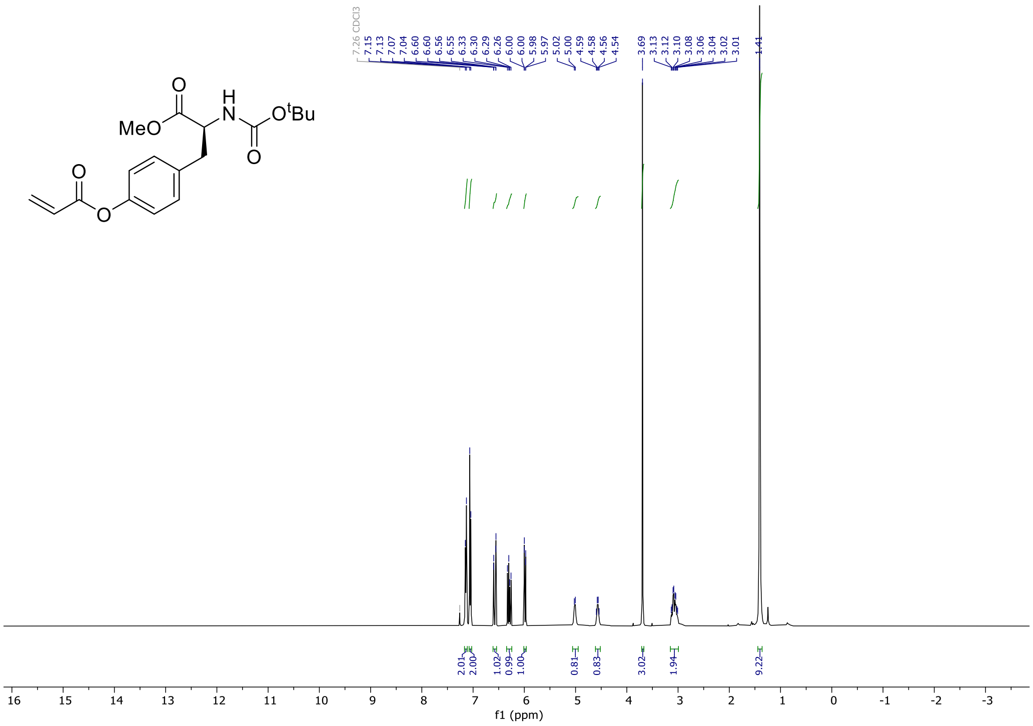

^{13}C-NMR (101 MHz, $CDCl_3$)

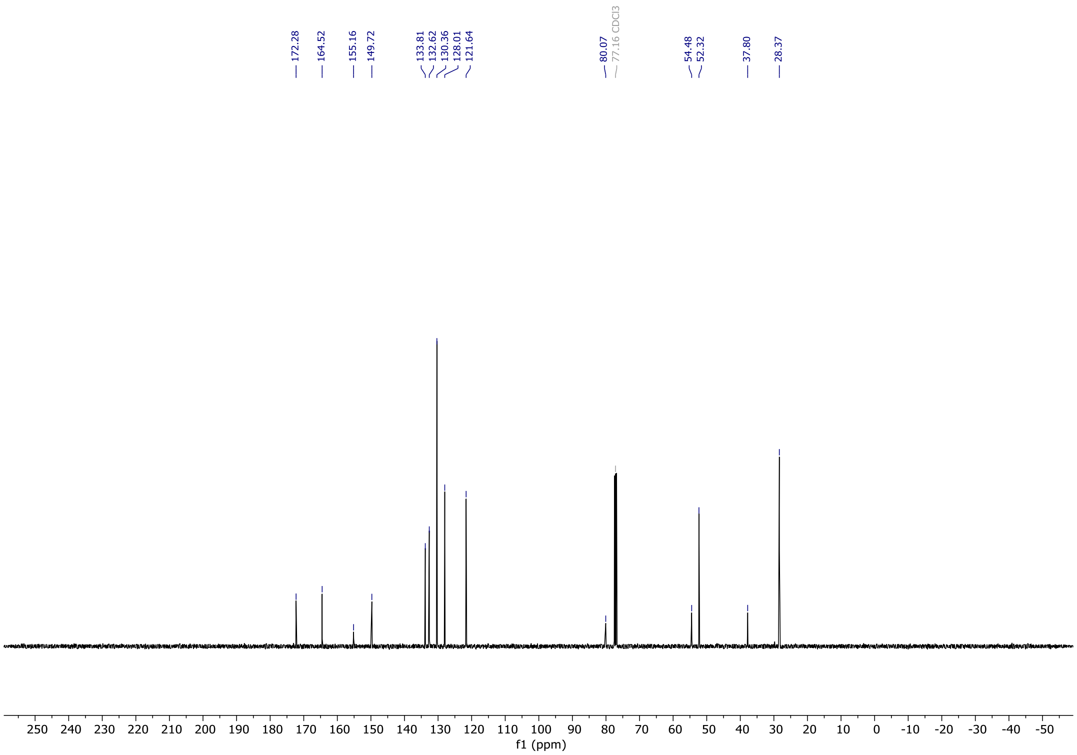

***n*-Butyl 3-(2-(pyridin-2-yl)phenyl)propanoate (3aa)**

^{1}H-NMR (500 MHz, $CDCl_3$)

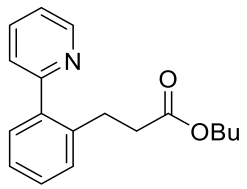

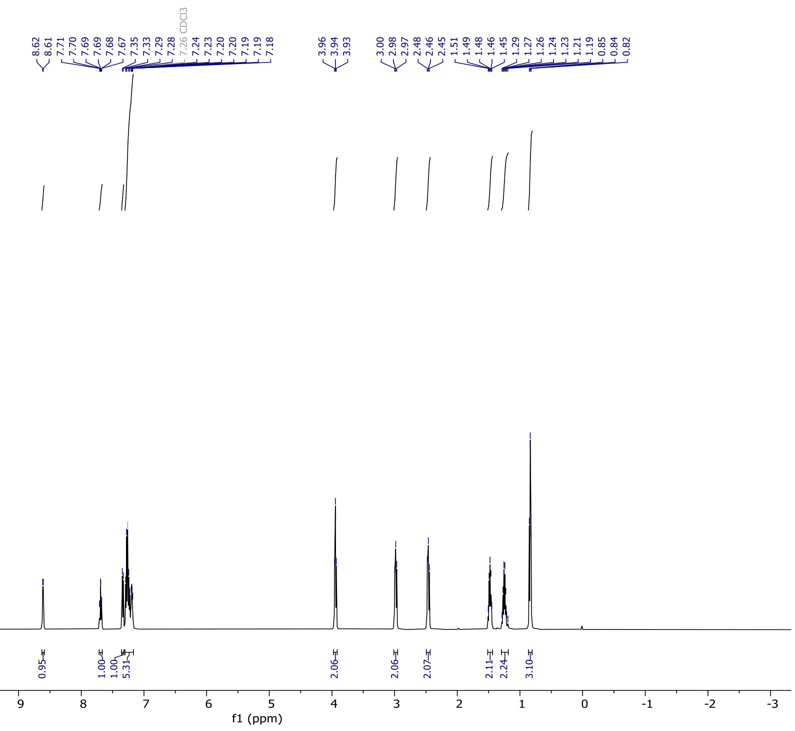

^{13}C-NMR (126 MHz, $CDCl_3$)

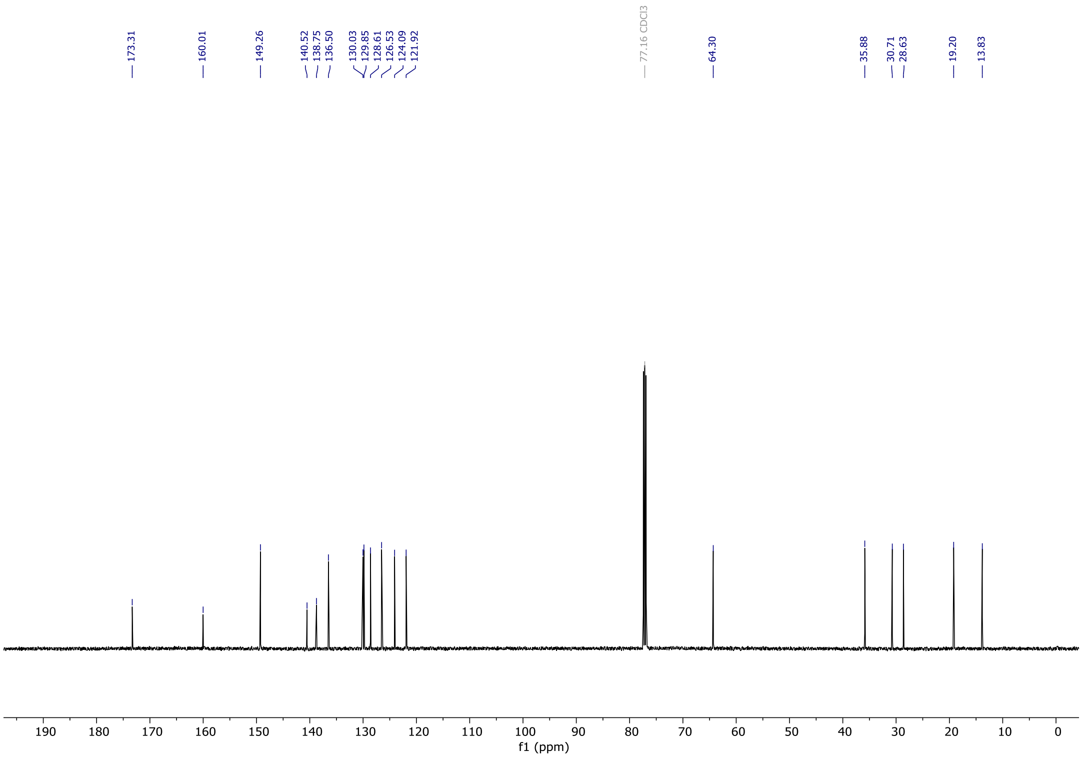

n-Butyl 3-(5-methoxy-2-(pyridin-2-yl)phenyl)propanoate (3ba)

^{1}H-NMR (400 MHz, $CDCl_3$)

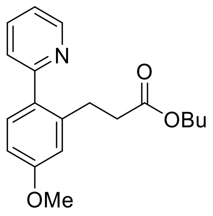

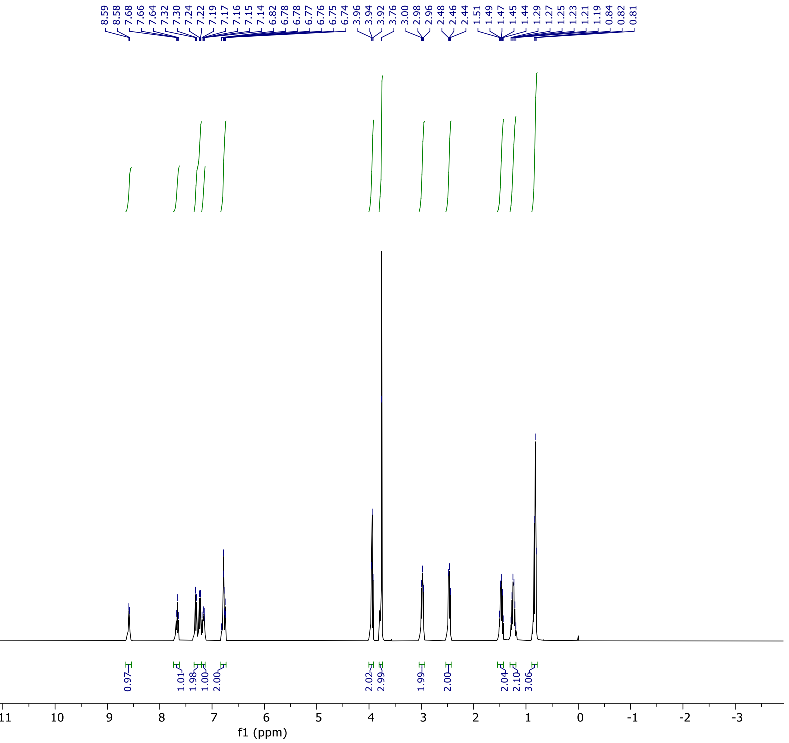

^{13}C-NMR (126 MHz, $CDCl_3$)

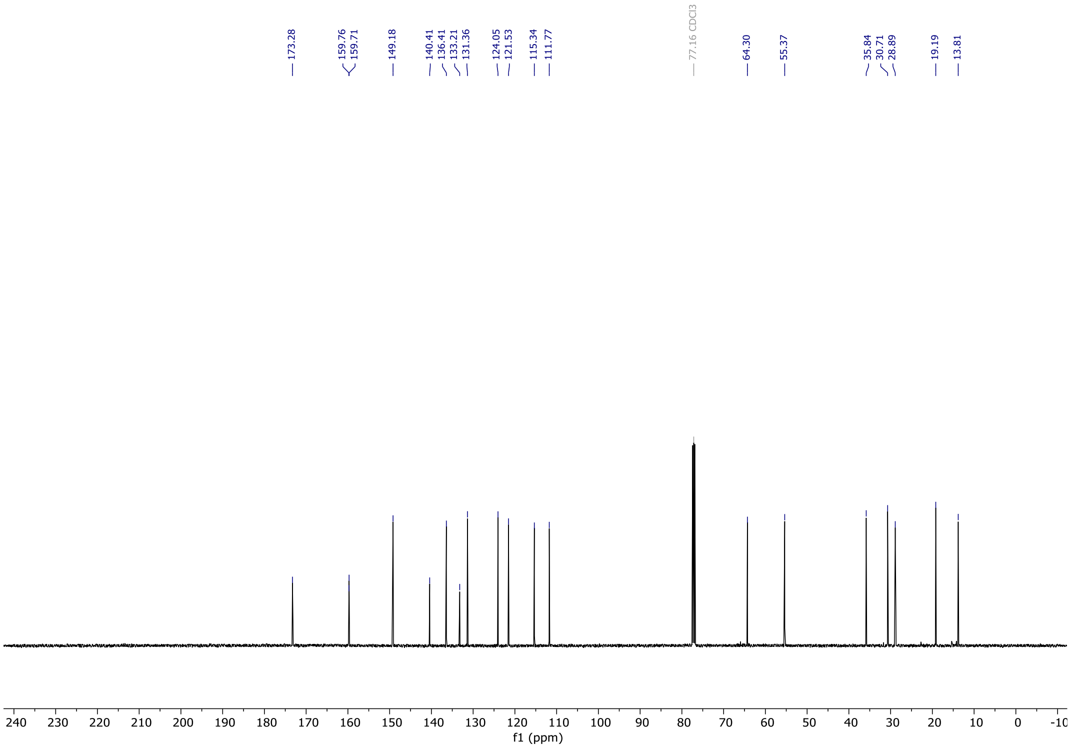

***n*-Butyl 3-(4-(pyridin-2-yl)-[1,1'-biphenyl]-3-yl)propanoate (3ca)**

^{1}H NMR (400 MHz, $CDCl_3$)

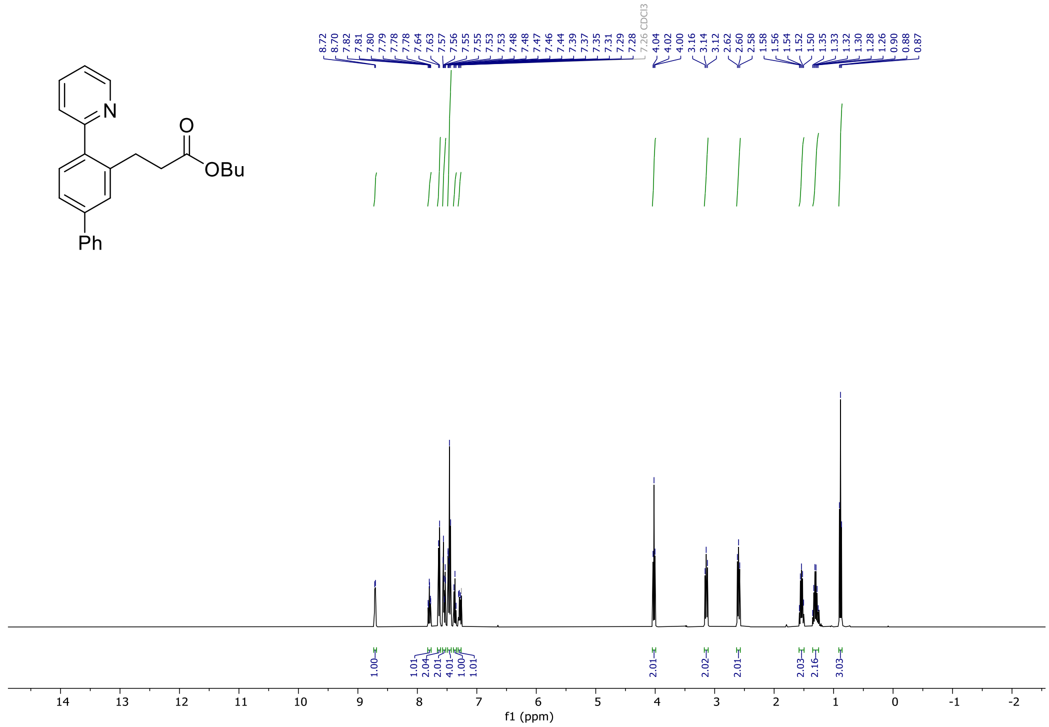

^{13}C NMR (101 MHz, $CDCl_3$)

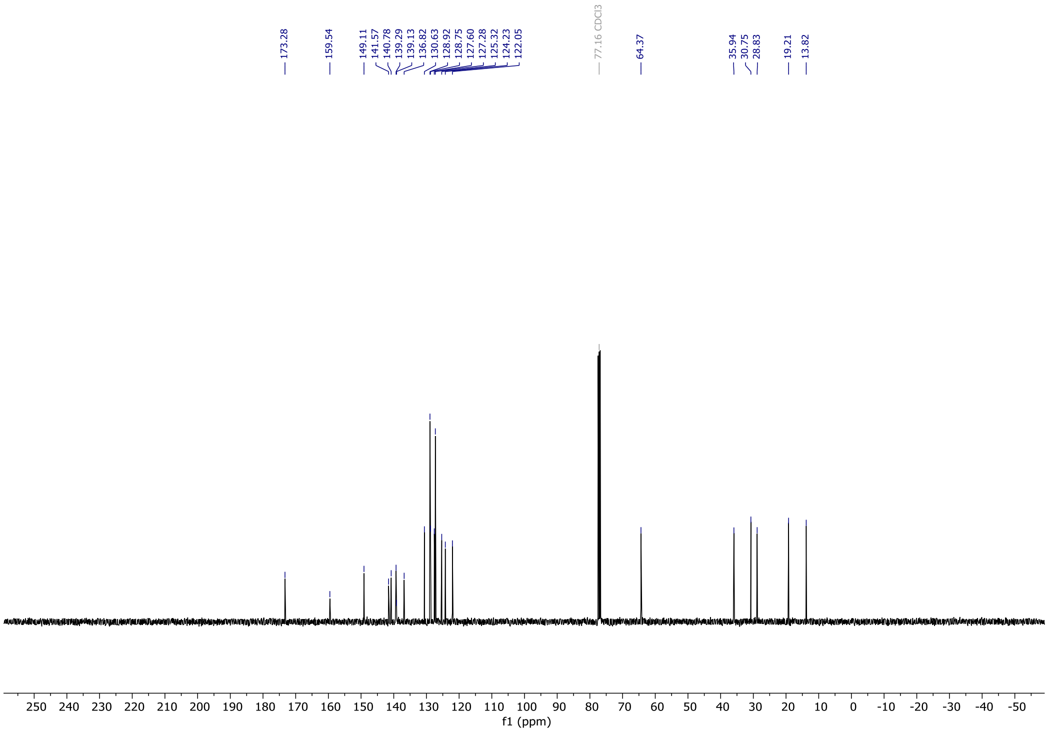

_n_-Butyl 3-(5-fluoro-2-(pyridin-2-yl)phenyl)propanoate (3da)

^{1}H-NMR (500 MHz, $CDCl_3$)

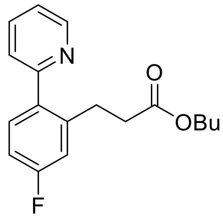

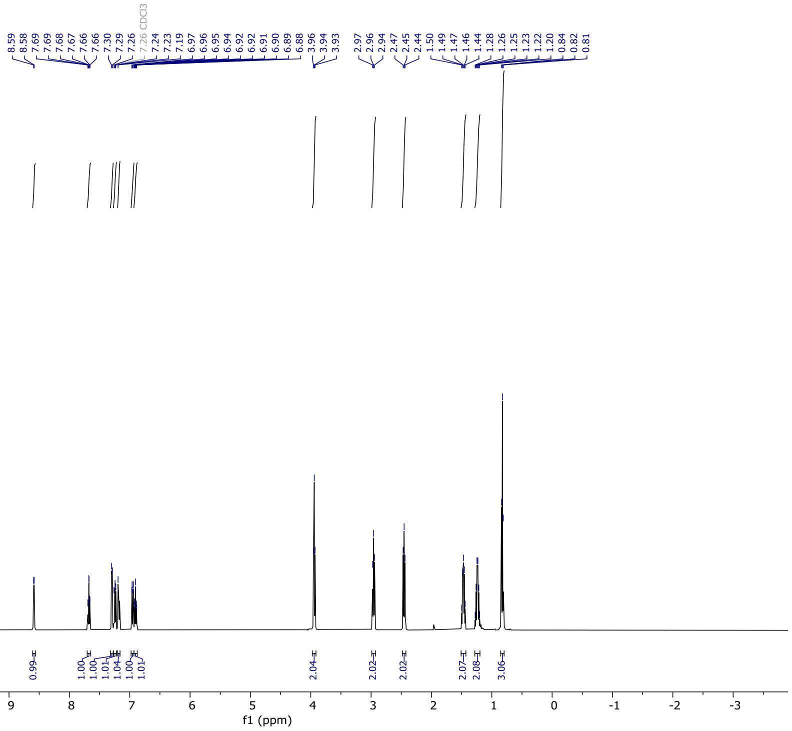

^{13}C-NMR (126 MHz, $CDCl_3$)

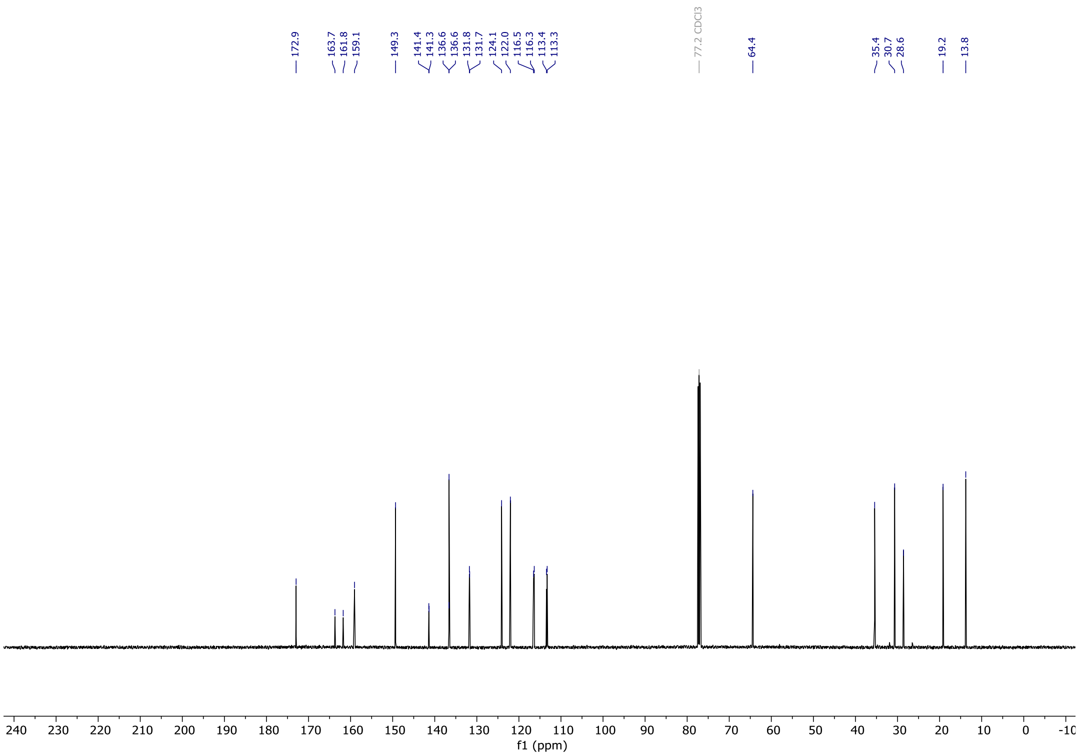

^{19}F-NMR (376 MHz, $CDCl_3$)

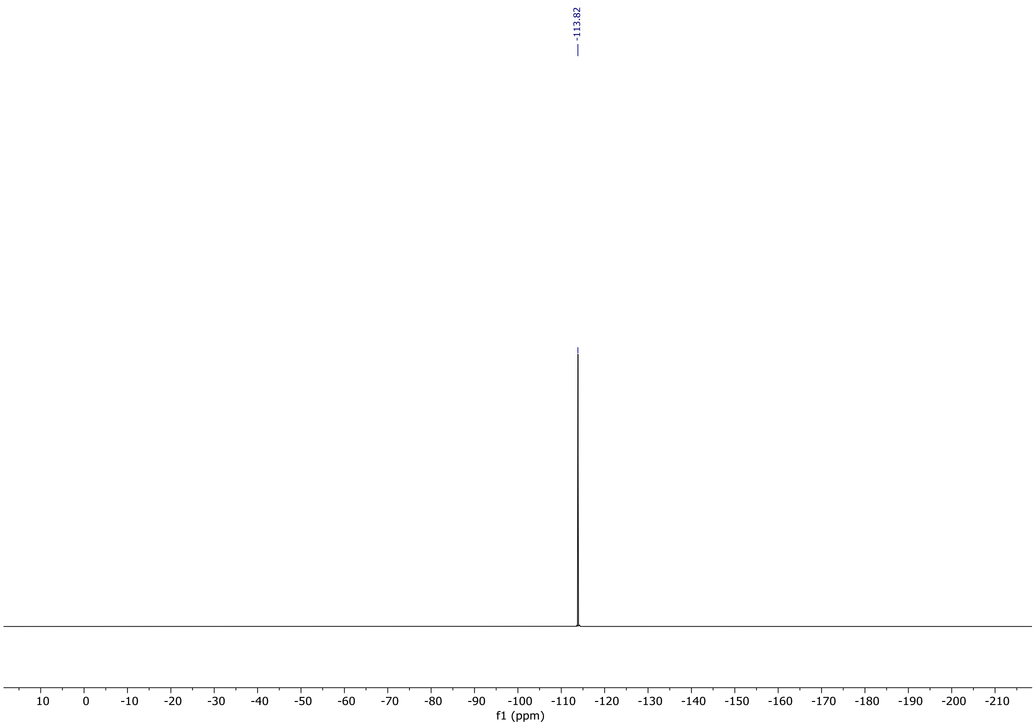

n-Butyl 3-(5-chloro-2-(pyridin-2-yl)phenyl)propanoate (3ea)

^{1}H-NMR (500 MHz, $CDCl_3$)

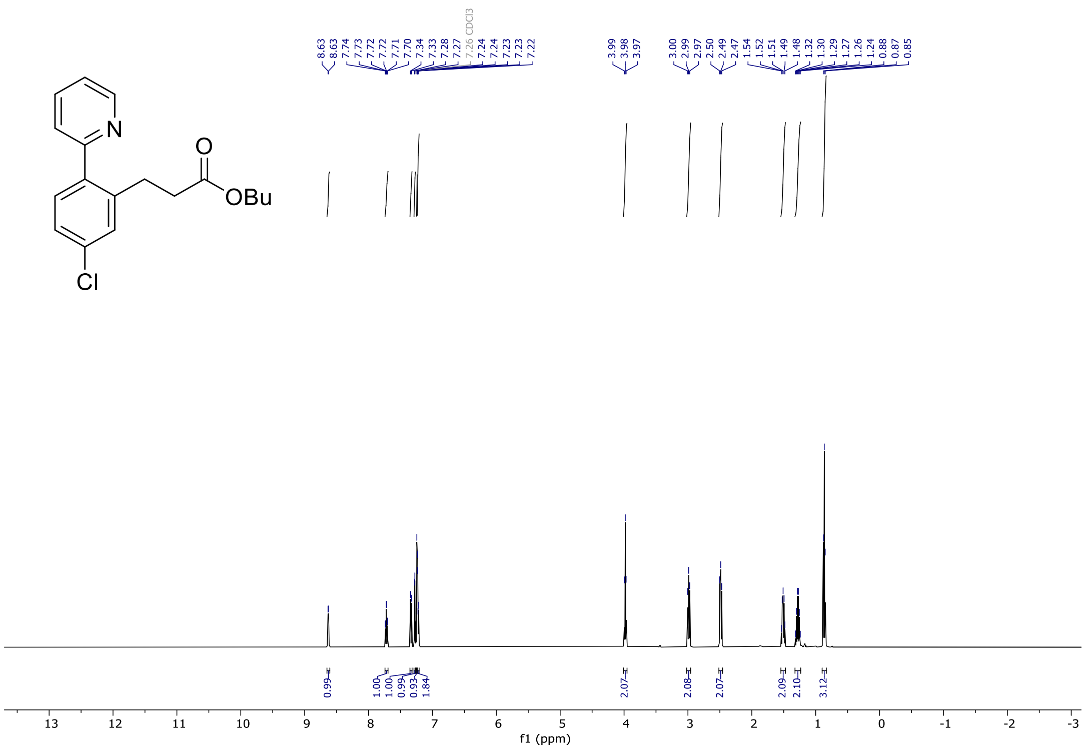

^{13}C-NMR (126 MHz, $CDCl_3$)

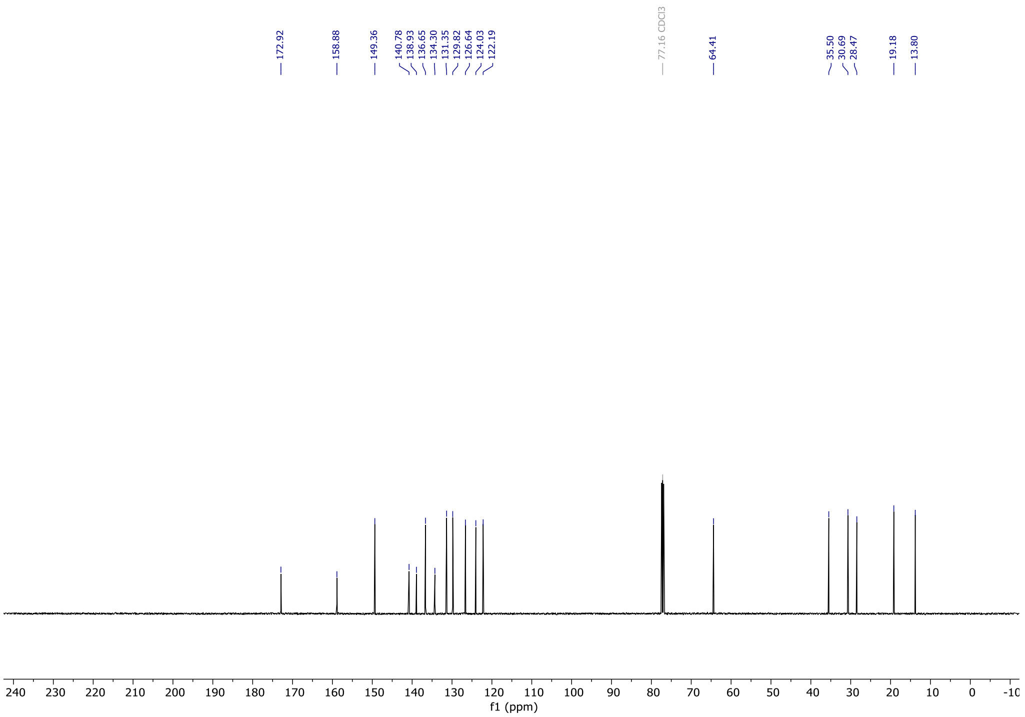

_n_-Butyl 3-(2-(pyridin-2-yl)-5-(trifluoromethyl)phenyl)propanoate (3fa)

^{1}H-NMR (400 MHz, $CDCl_3$)

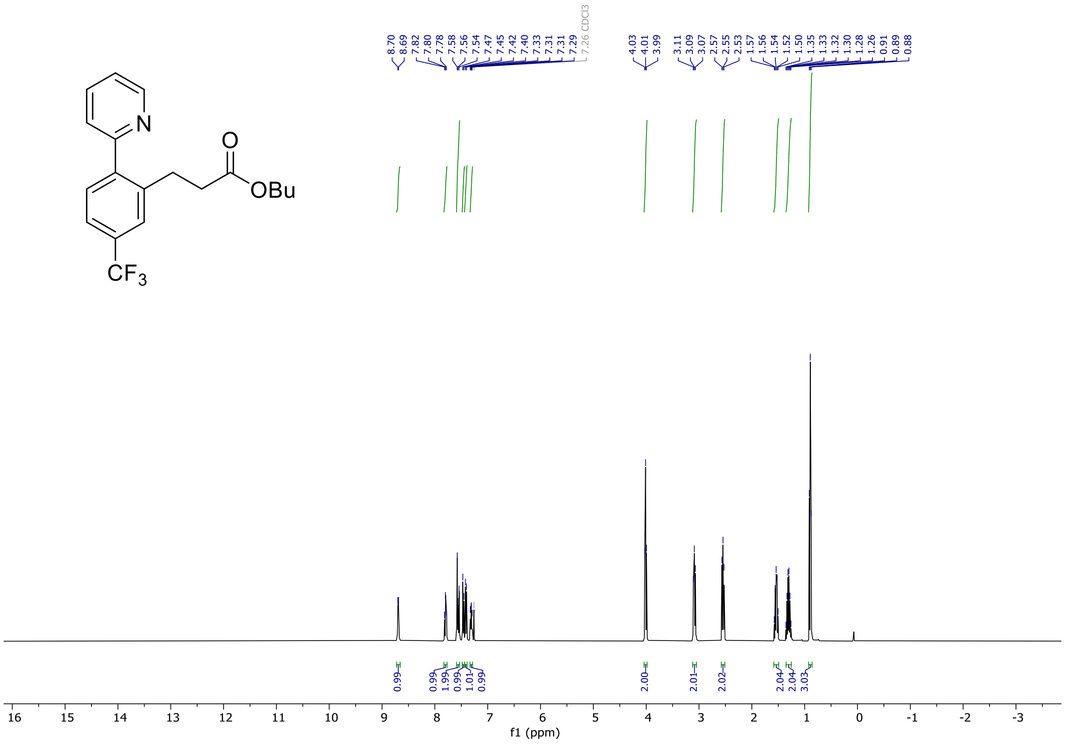

^{13}C-NMR (101 MHz, $CDCl_3$)

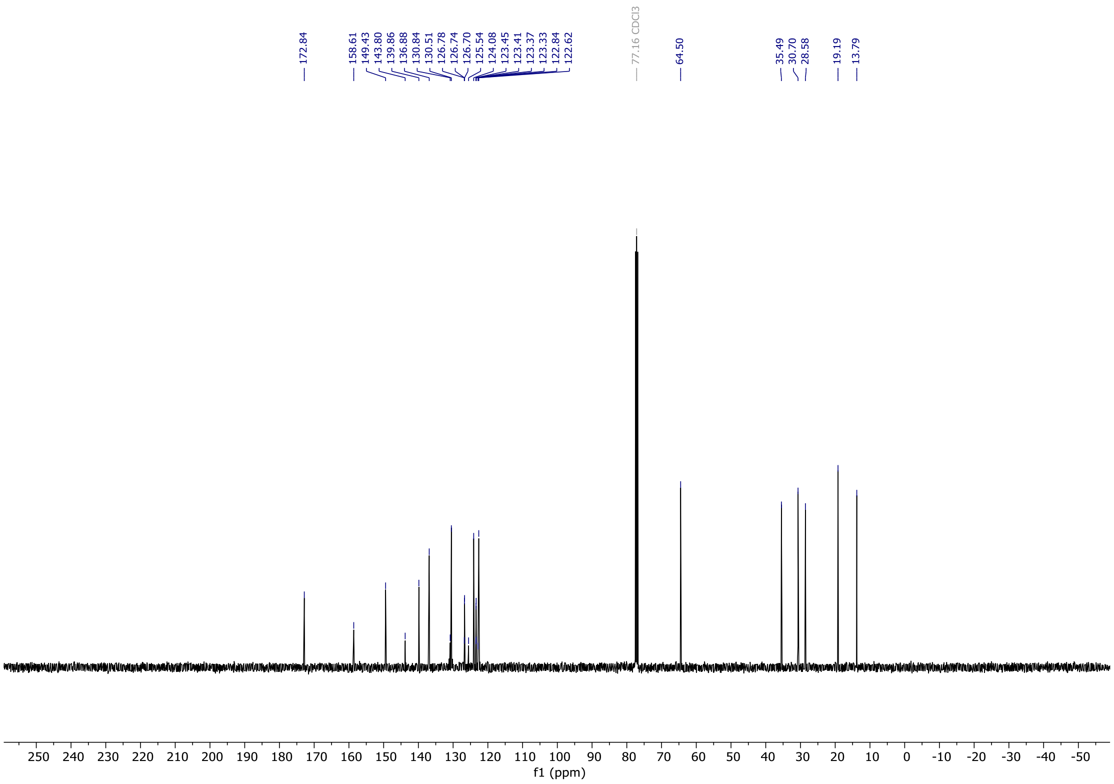

^{19}F-NMR (376 MHz, $CDCl_3$)

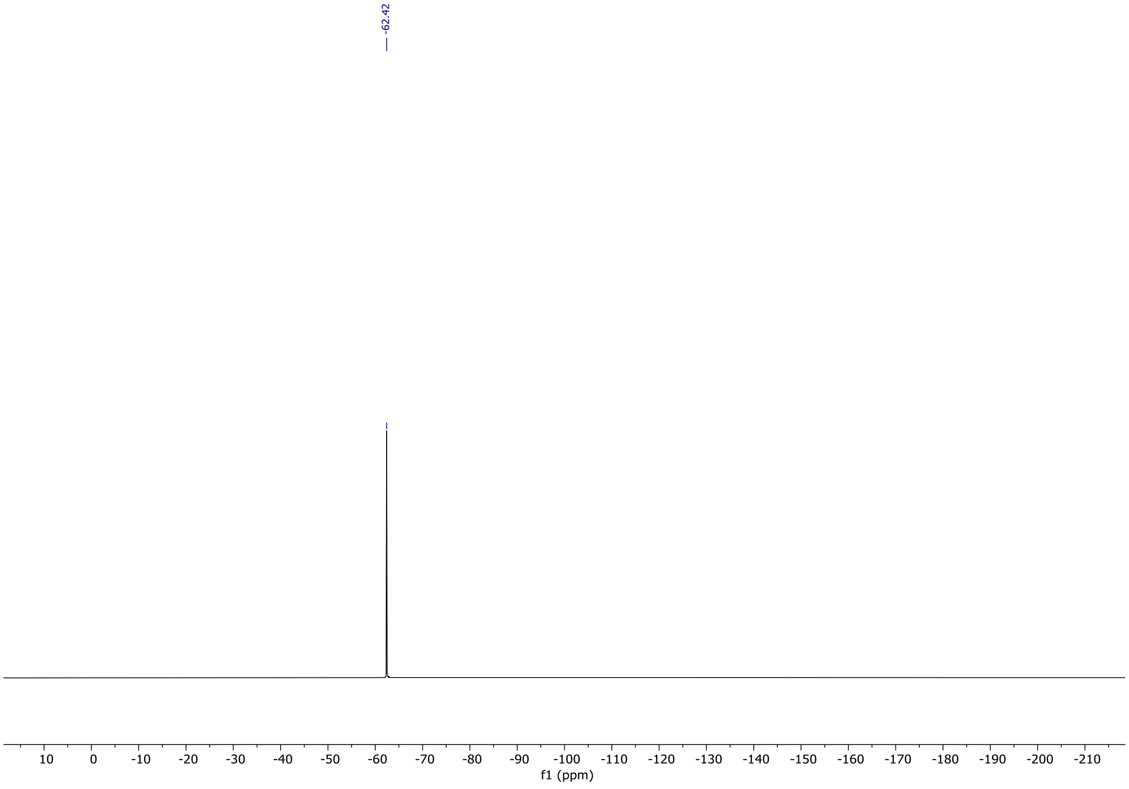

Methyl 3-(3-butoxy-3-oxopropyl)-4-(pyridin-2-yl)benzoate (3ga)

^{1}H-NMR (500 MHz, $CDCl_3$)

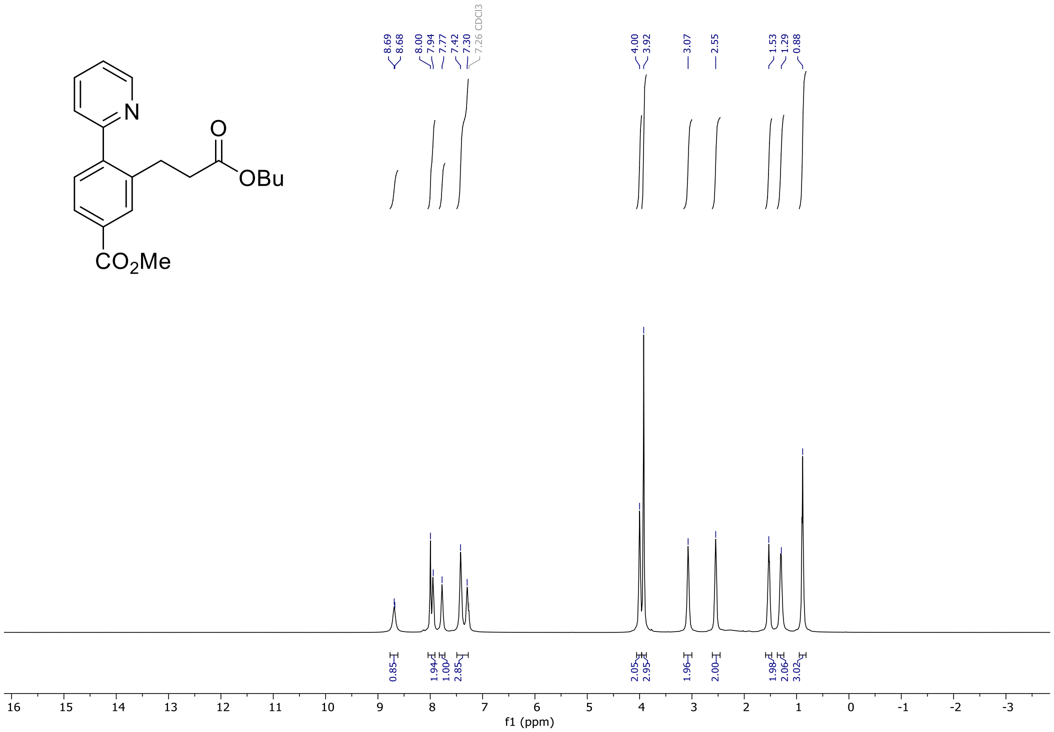

^{13}C-NMR (126 MHz, $CDCl_3$)

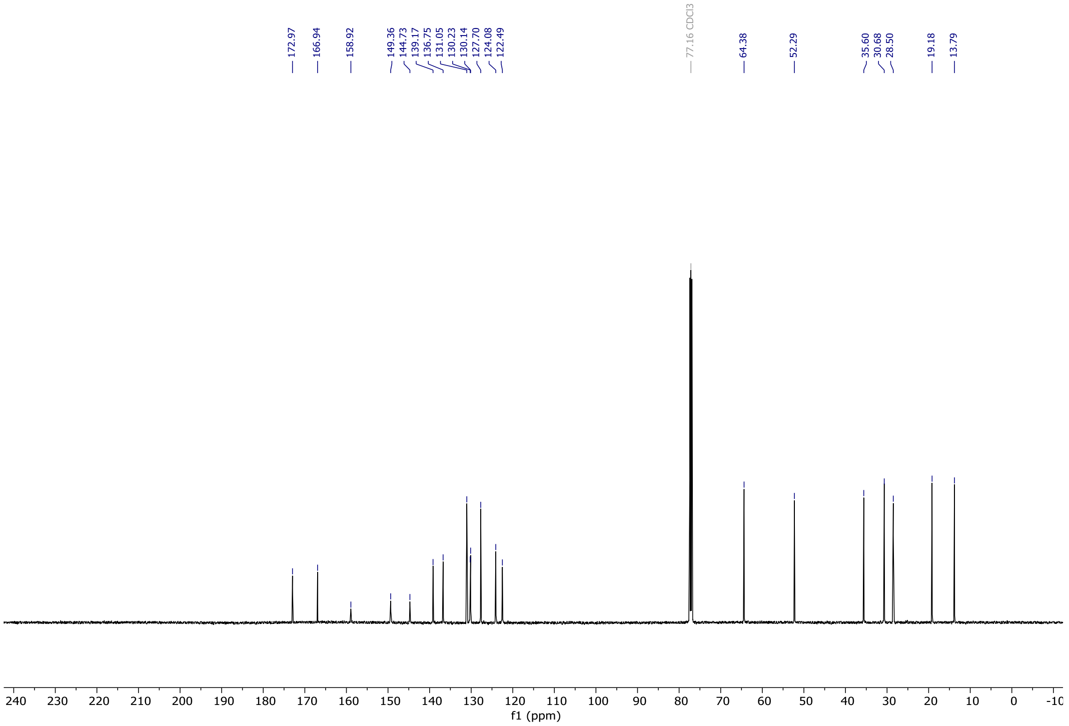

n-Butyl 3-(3-methoxy-2-(pyridin-2-yl)phenyl)propanoate (3ha)

^{1}H-NMR (400 MHz, $CDCl_3$)

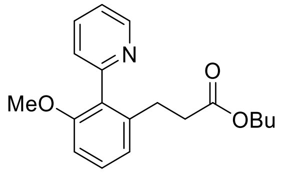

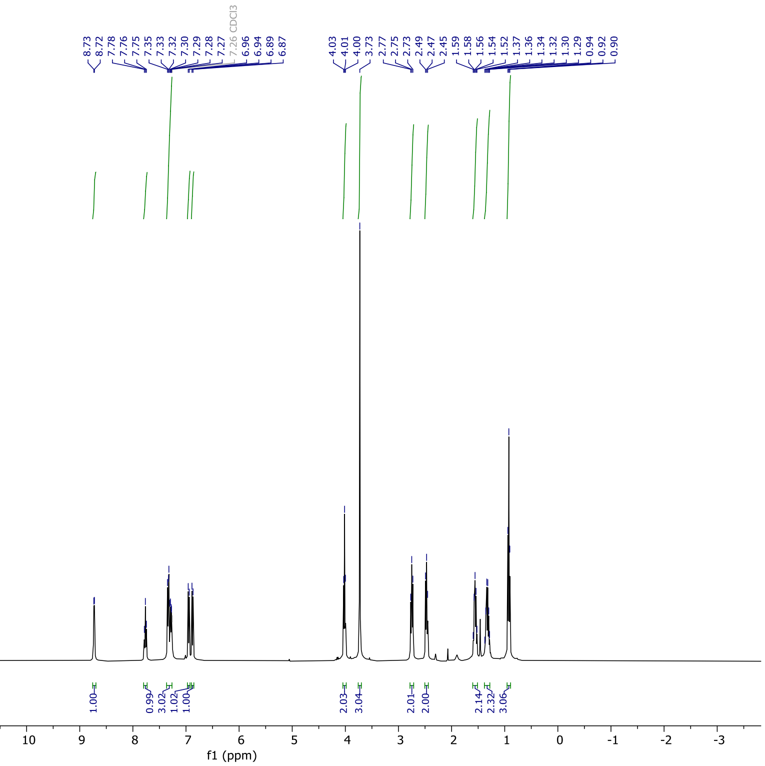

^{13}C-NMR (126 MHz, $CDCl_3$)

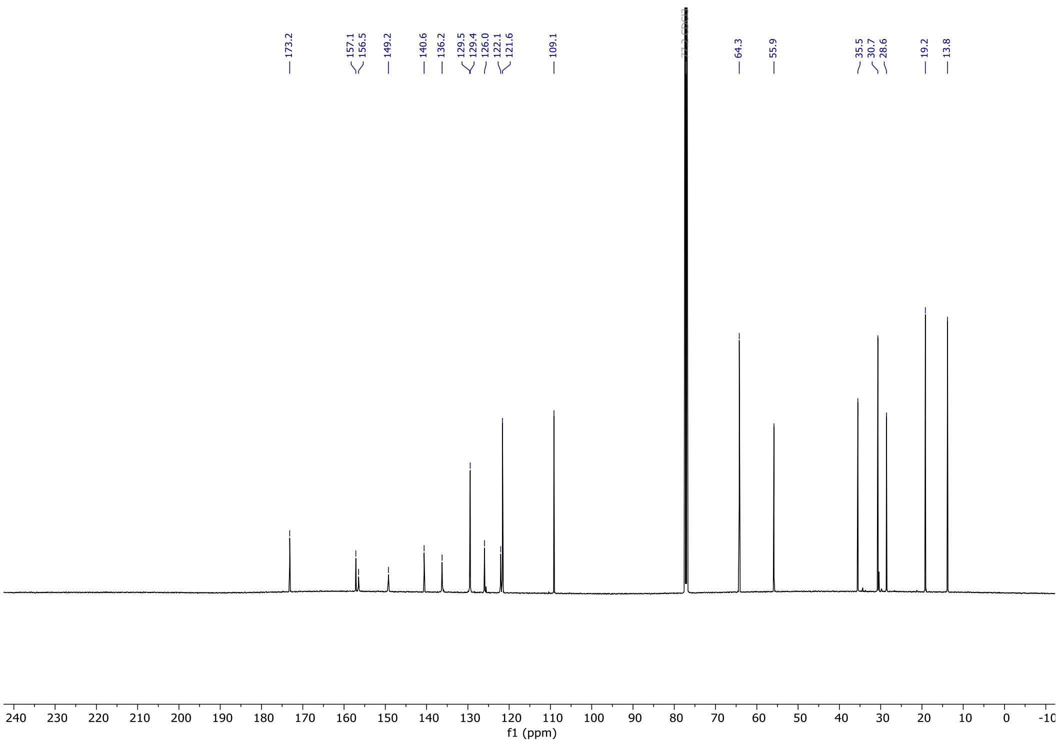

***n*-Butyl 3-(3-fluoro-2-(pyridin-2-yl)phenyl)propanoate (3ia)**

^{1}H-NMR (400 MHz, $CDCl_3$)

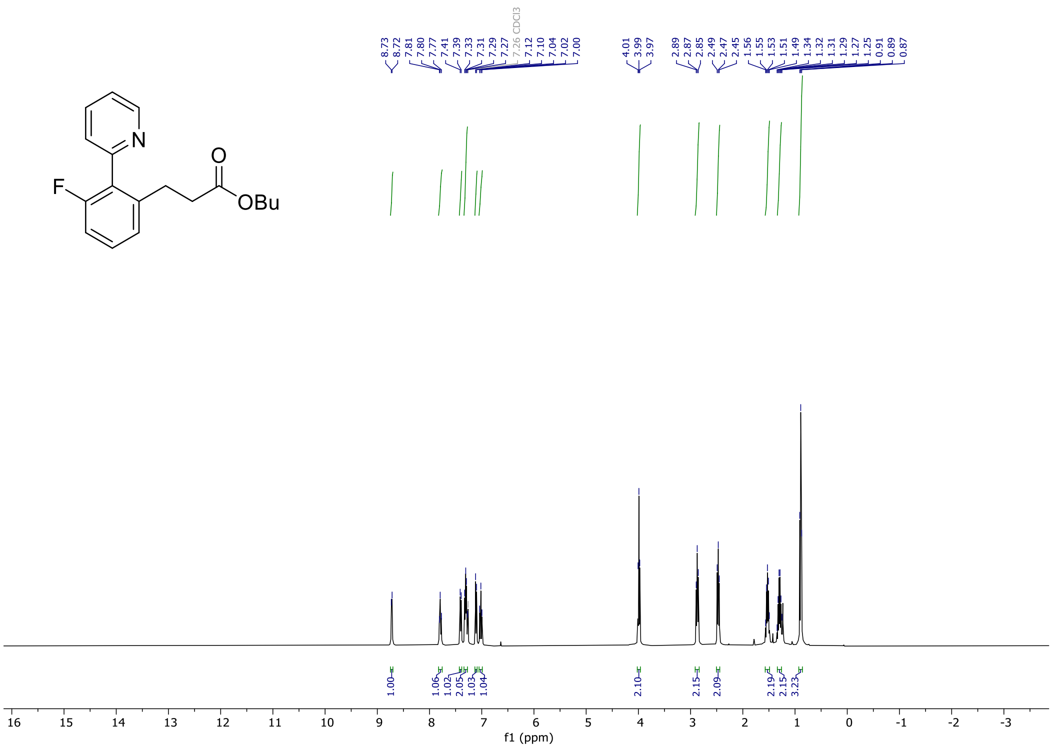

^{13}C-NMR (101 MHz, $CDCl_3$)

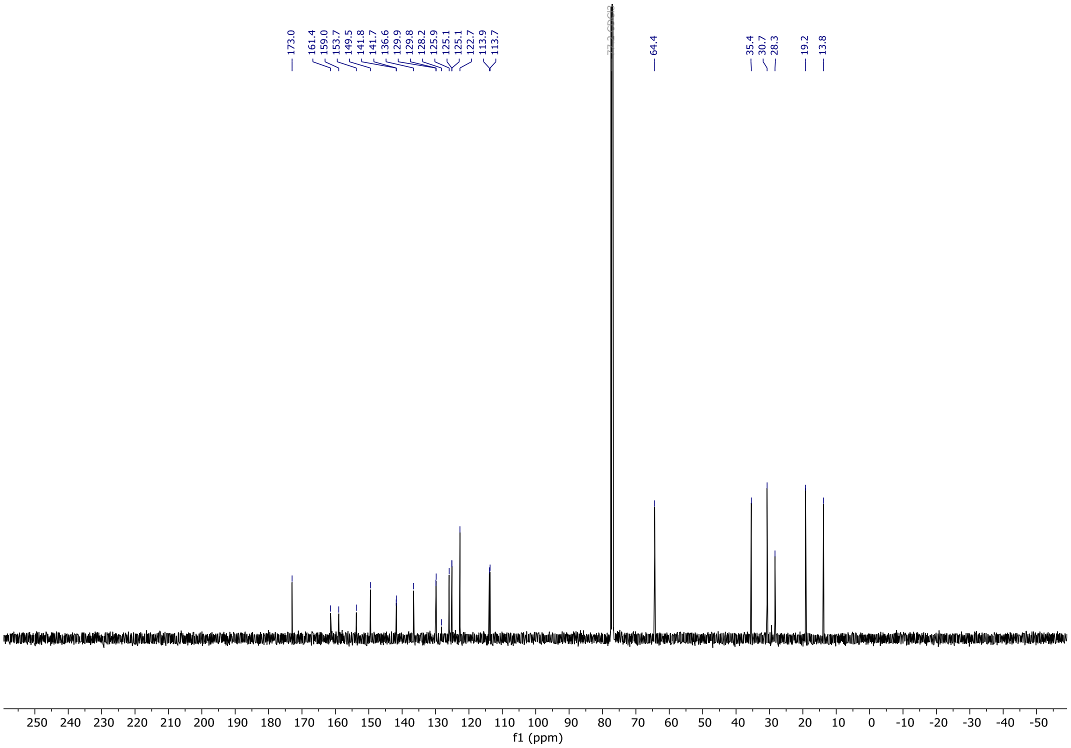

^{19}F-NMR (376 MHz, $CDCl_3$)

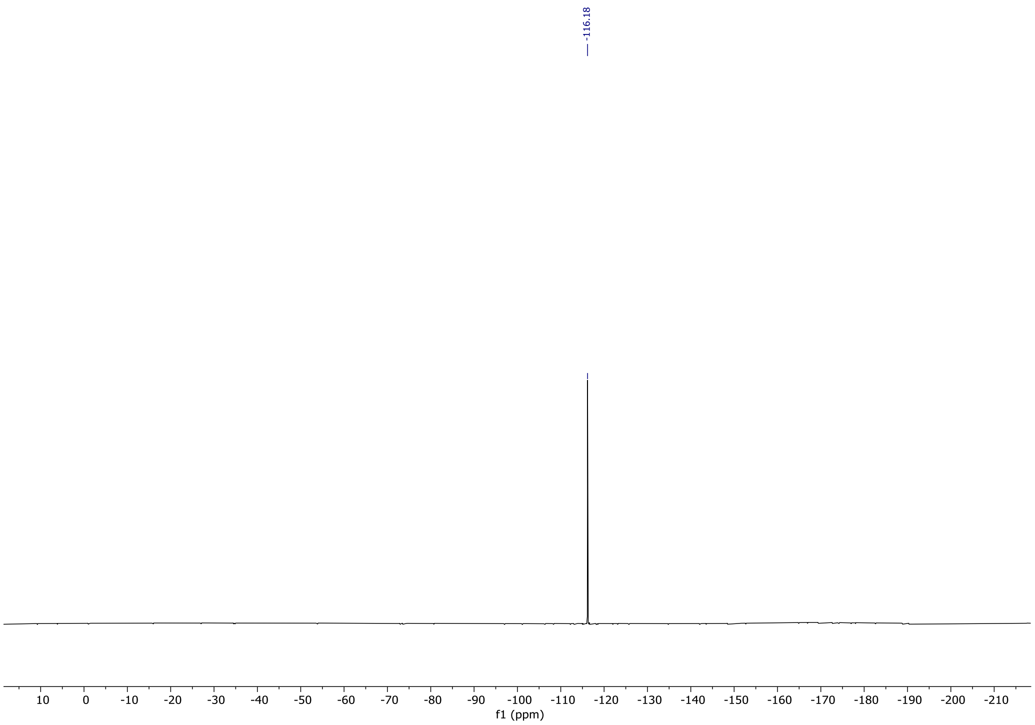

***n*-Butyl 3-(4-methyl-2-(pyridin-2-yl)phenyl)propanoate (3ja)**

^{1}H-NMR (400 MHz, $CDCl_3$)

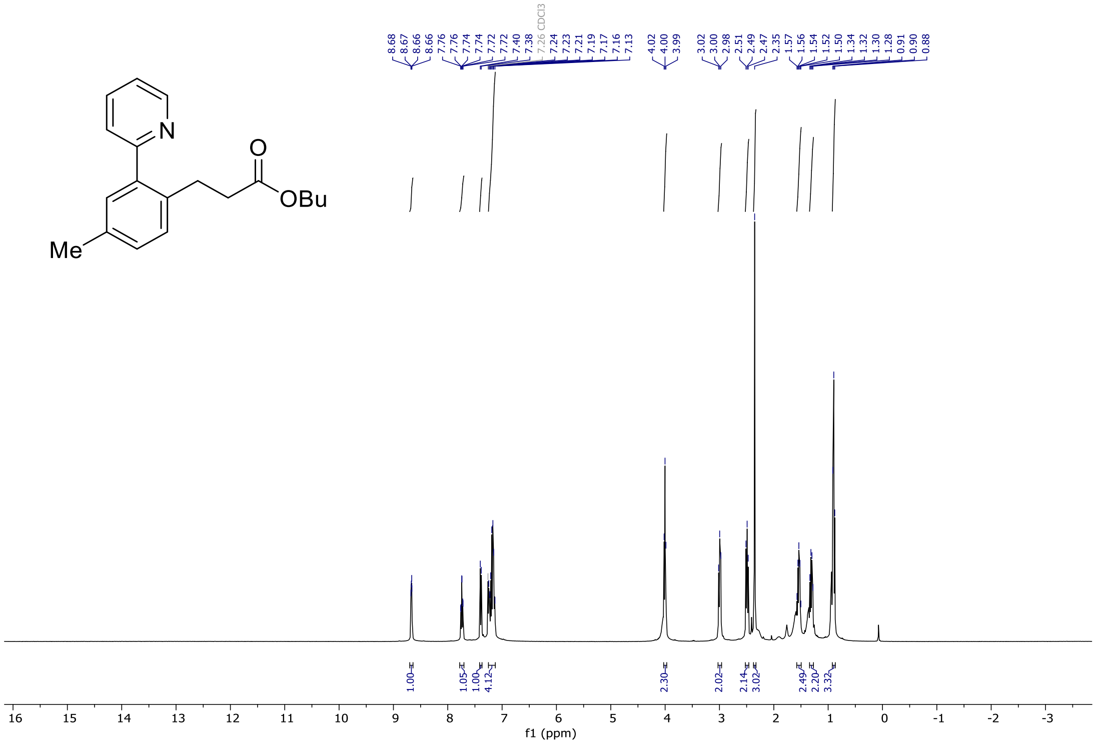

^{13}C-NMR (101 MHz, $CDCl_3$)

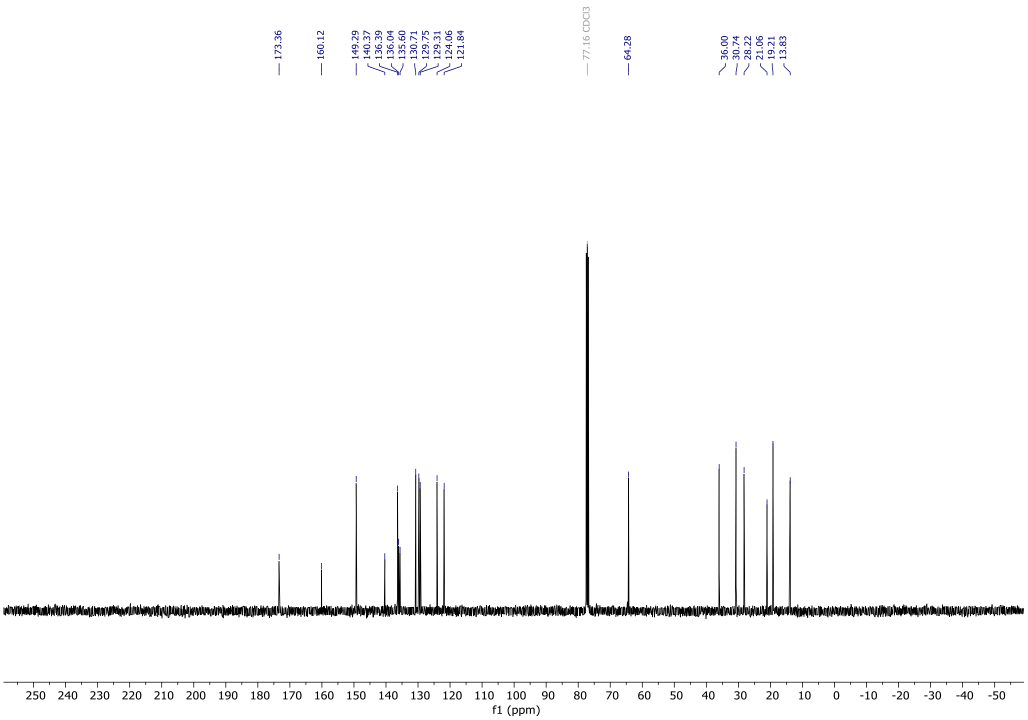

n-Butyl 3-(2-(pyridin-2-yl)-4-(trifluoromethyl)phenyl)propanoate (3ka)

^{1}H-NMR (400 MHz, $CDCl_3$)

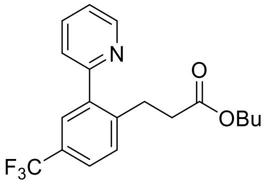

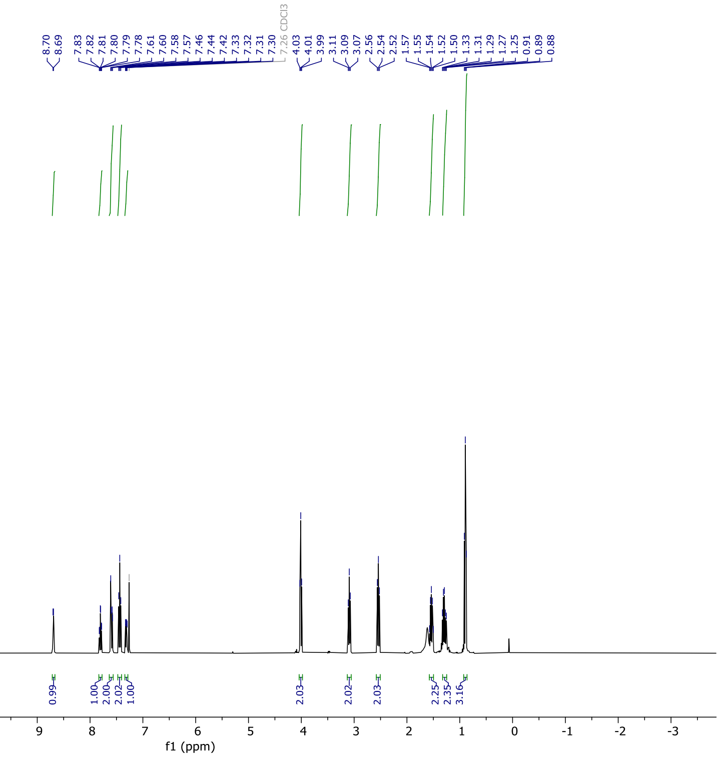

^{13}C-NMR (126 MHz, $CDCl_3$)

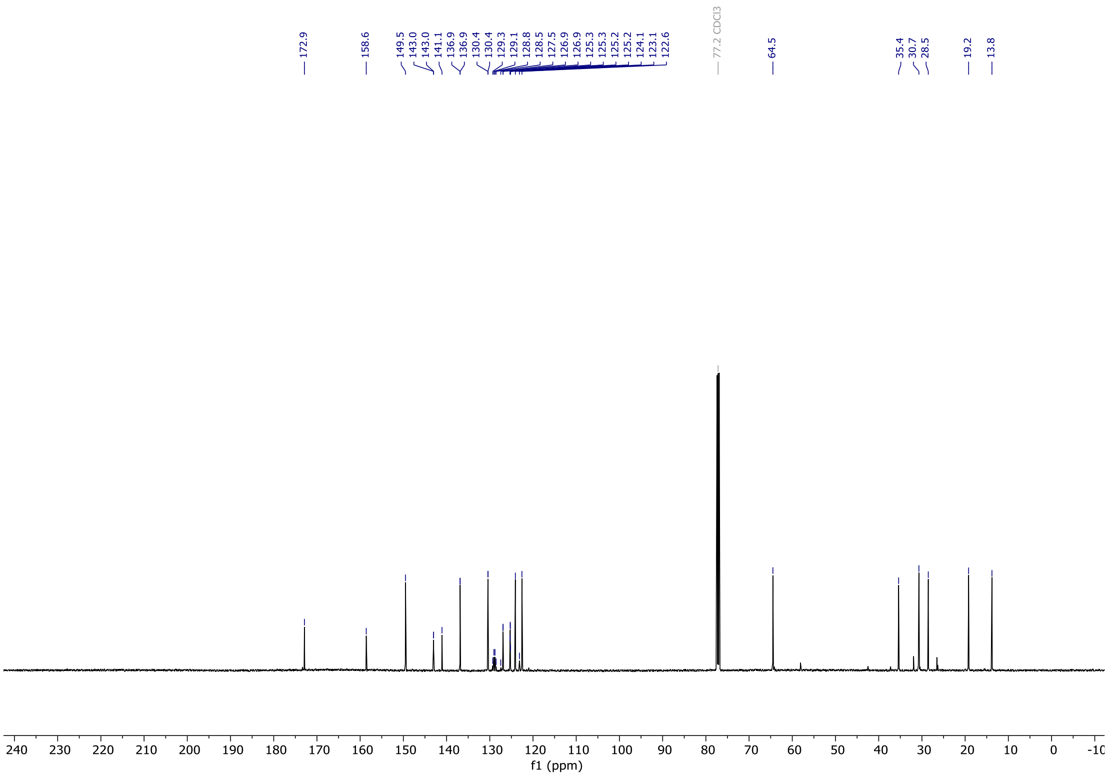

^{19}F-NMR (376 MHz, $CDCl_3$)

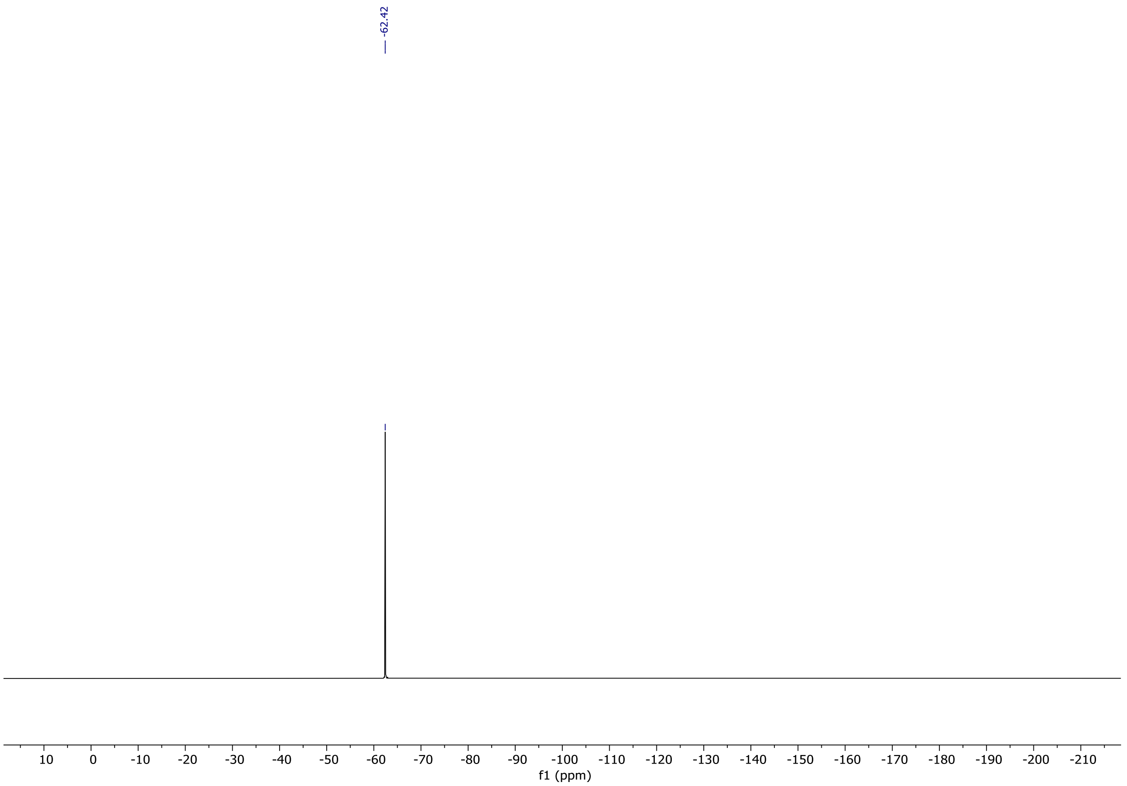

Methyl 4-(3-butoxy-3-oxopropyl)-3-(pyridin-2-yl)benzoate (3la)

^{1}H-NMR (400 MHz, $CDCl_3$)

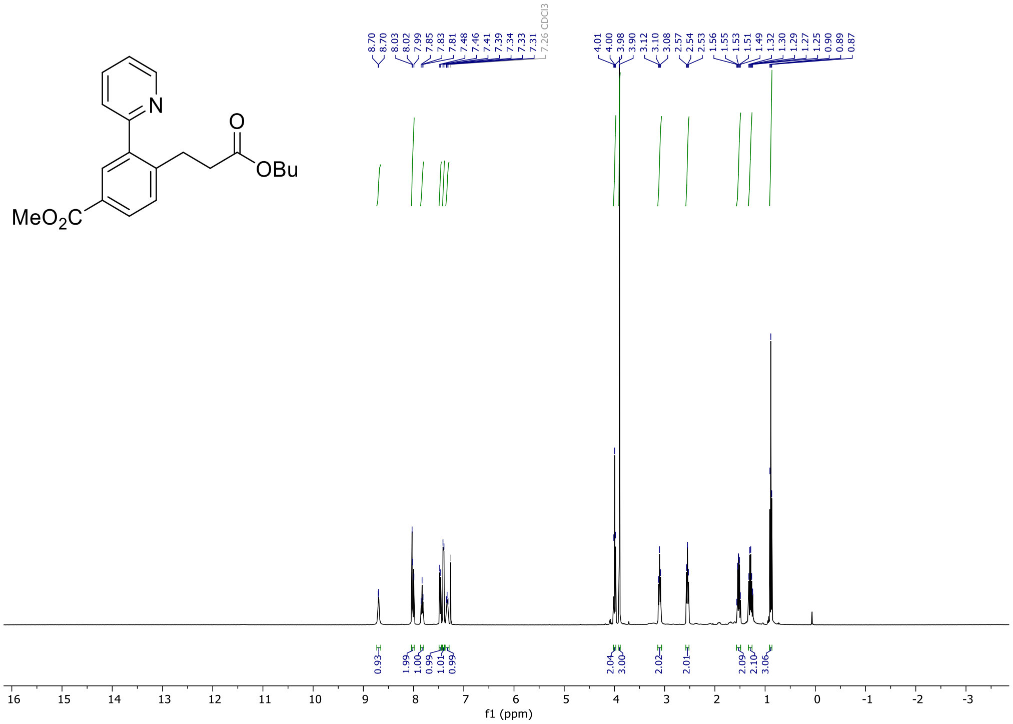

^{13}C-NMR (126 MHz, $CDCl_3$)

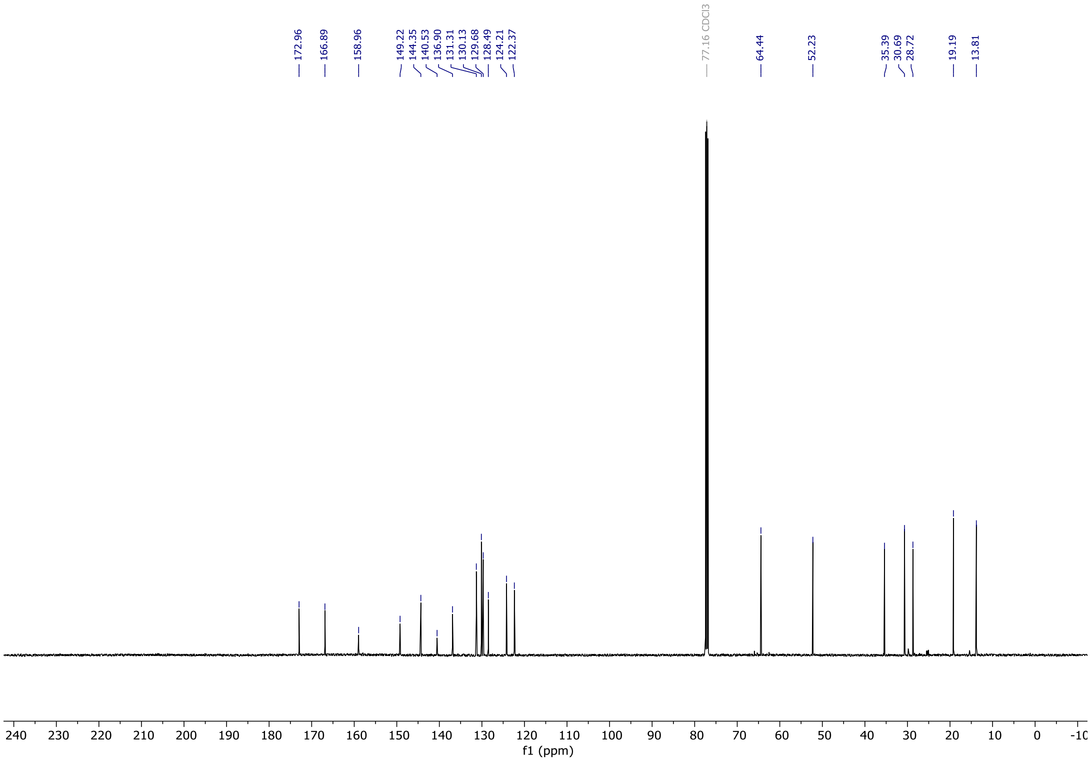

_n_-Butyl 3-(2-(5-methylpyridin-2-yl)phenyl)propanoate (3ma)

^{1}H-NMR (500 MHz, $CDCl_3$)

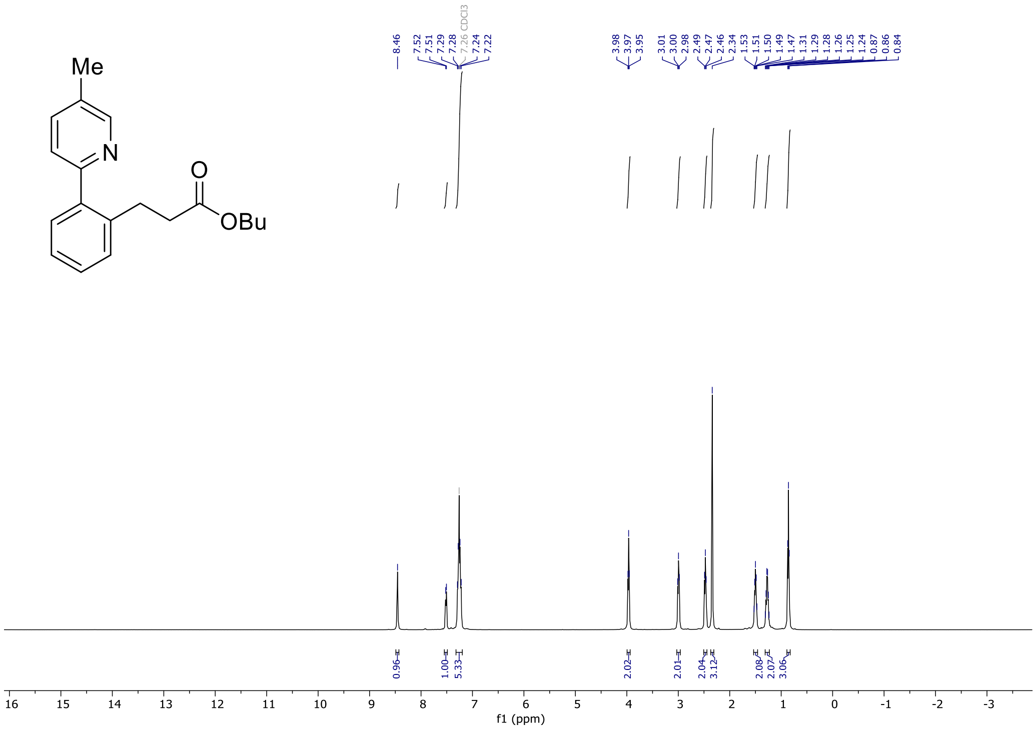

^{13}C-NMR (126 MHz, $CDCl_3$)

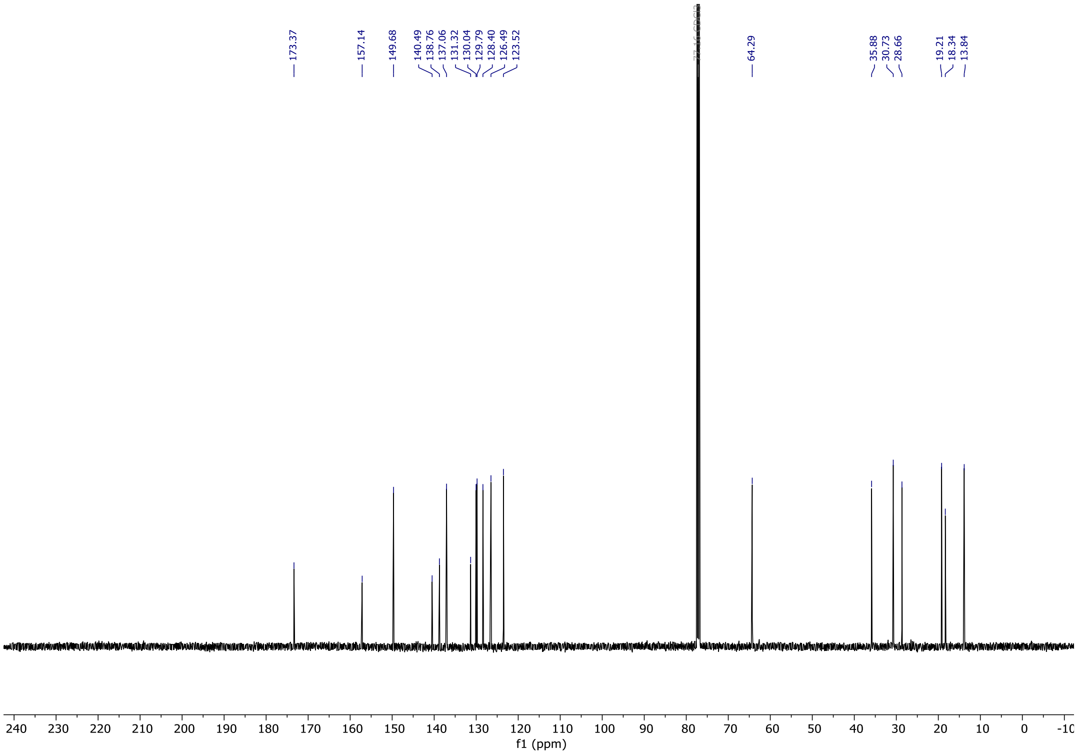

***n*-Butyl 3-(2-(1H-pyrazol-1-yl)phenyl)propanoate (3na)**

^{1}H-NMR (500 MHz, $CDCl_3$)

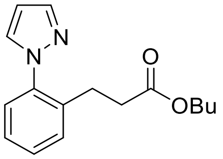

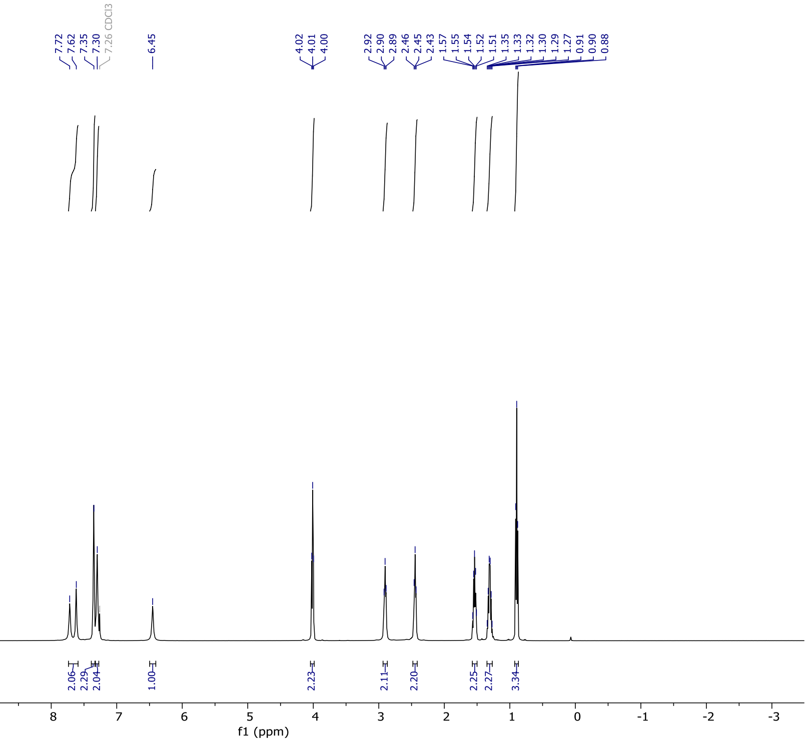

^{13}C-NMR (126 MHz, $CDCl_3$)

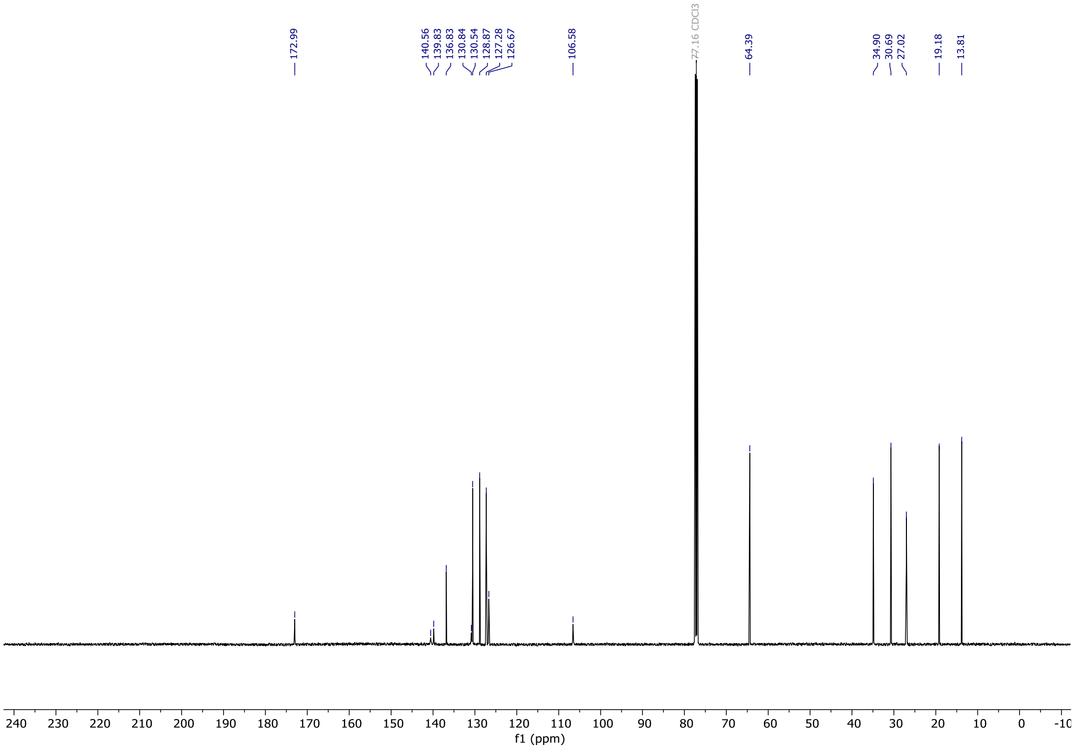

n-Butyl 3-(2-(2-oxo-2,3-dihydro-1H-benzo[e][1,4]diazepin-5-yl)phenyl)propanoate (3oa)

^{1}H-NMR (400 MHz, $CDCl_3$)

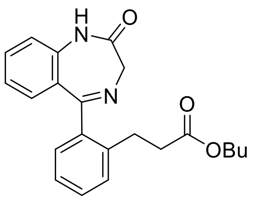

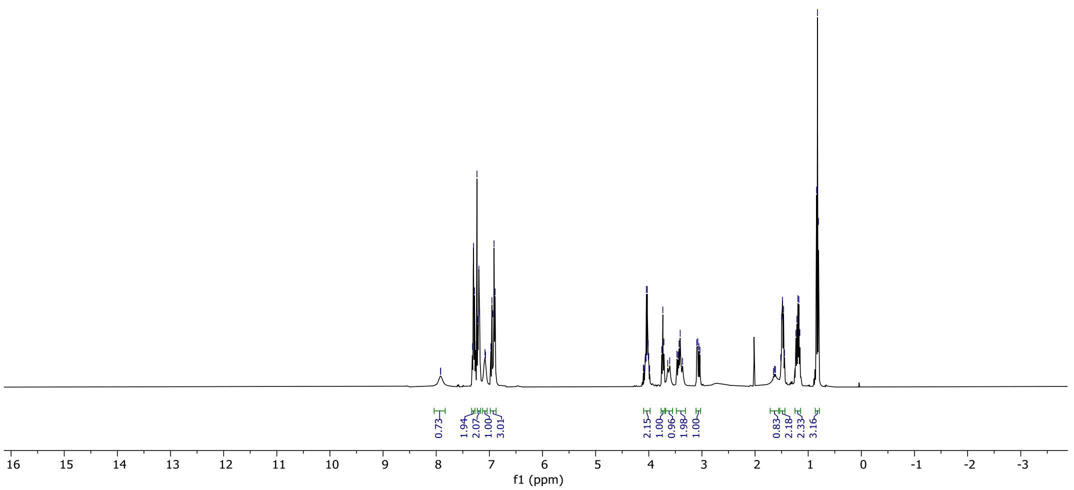

^{13}C-NMR (126 MHz, $CDCl_3$)

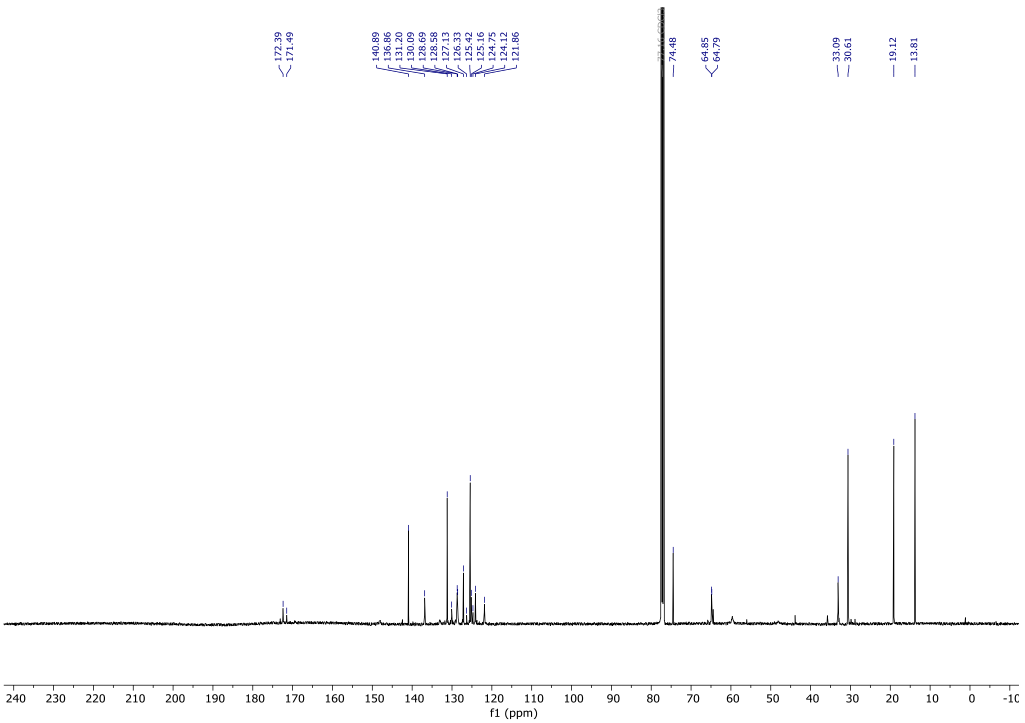

_n_-Butyl 3-(2-(7-chloro-1-methyl-2-oxo-2,3-dihydro-1H-benzo[e][1,4]diazepin-5-yl)phenyl)propanoate (3pa)

^{1}H-NMR (400 MHz, $CDCl_3$)

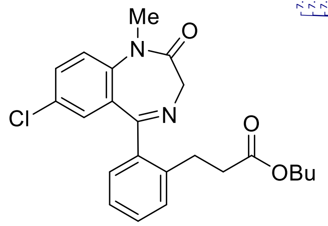

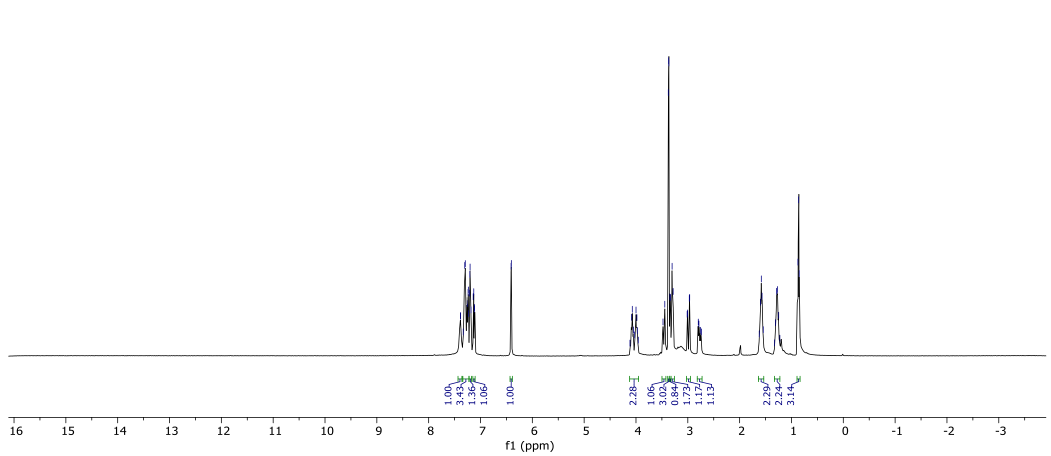

^{13}C-NMR (126 MHz, $CDCl_3$)

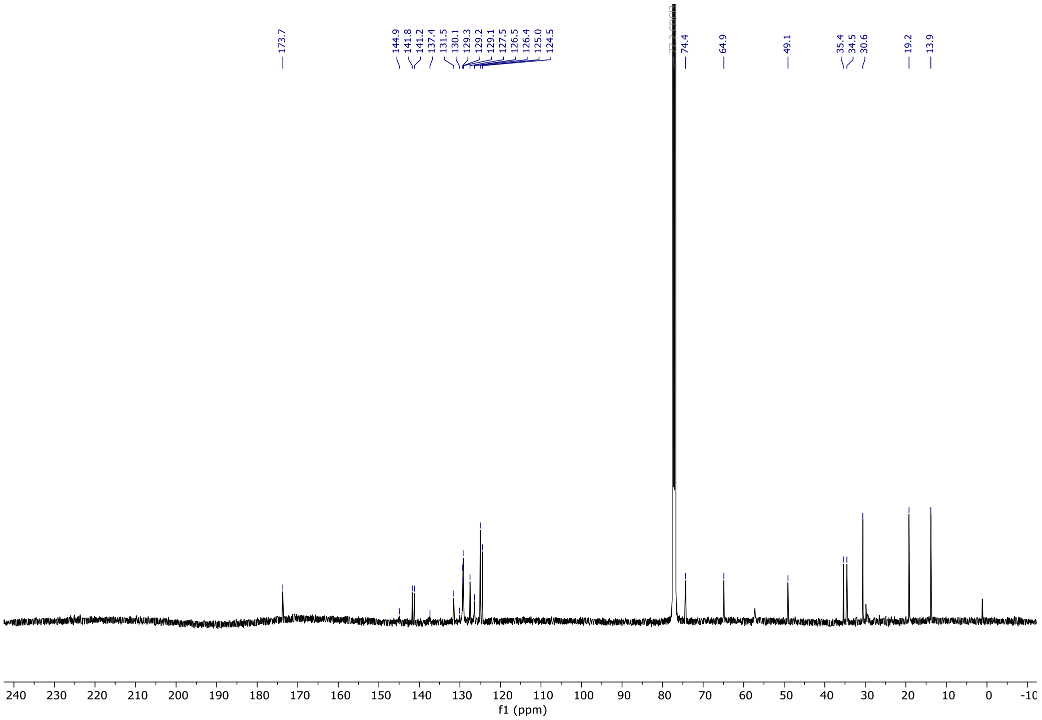

_n_-Butyl 3-(2-fluoro-6-(pyridin-2-yl)phenyl)propanoate (3qa) and _n_-Butyl 3-(4-fluoro-2-(pyridin-2-yl)phenyl)propanoate (3qa')

^{1}H NMR (400 MHz, $CDCl_3$)

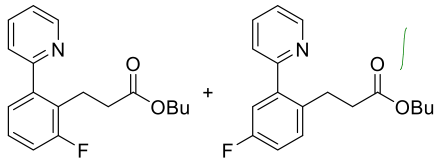

3qa   3qa'

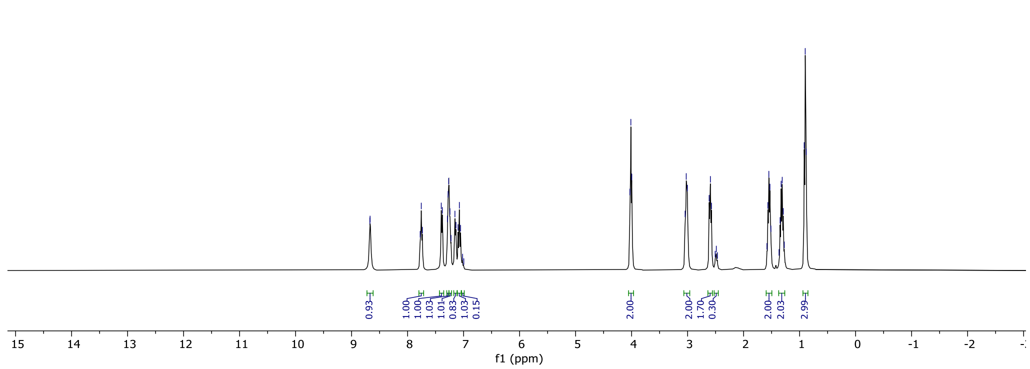

^{13}C NMR (101 MHz, $CDCl_3$)

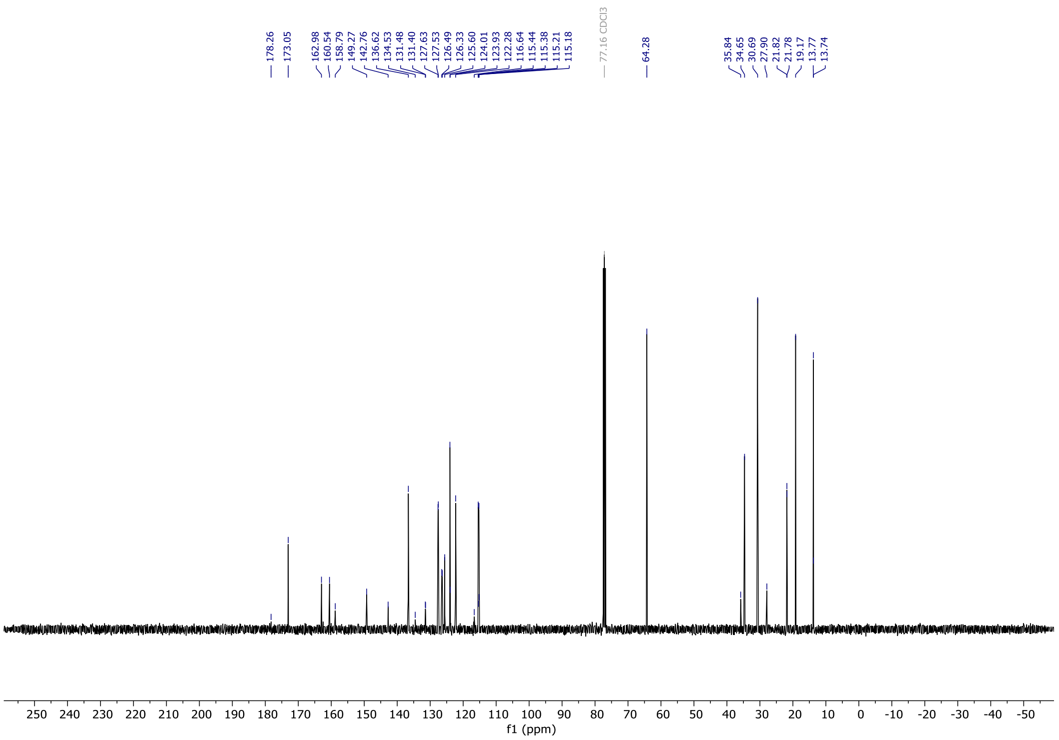

^{19}F NMR (376 MHz, $CDCl_3$)

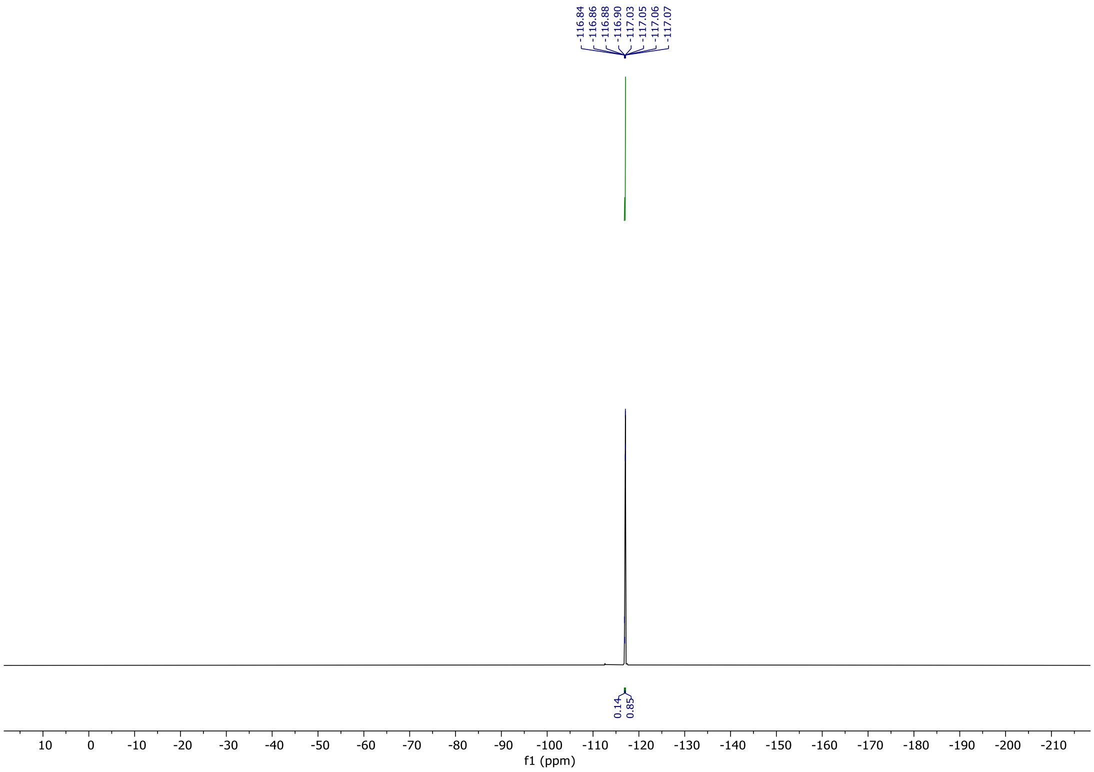

2-D COSY-NMR (400 MHz, $CDCl_3$)

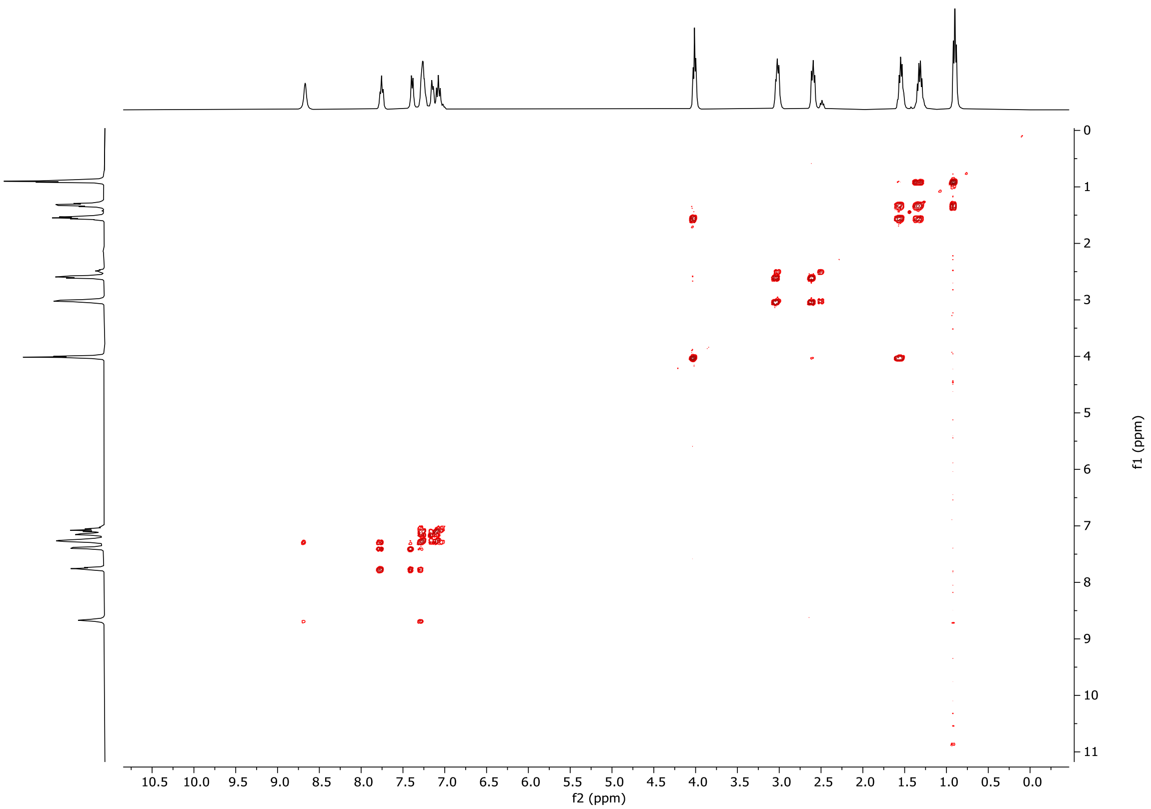

2-D NOESY-NMR (400 MHz, $CDCl_3$)

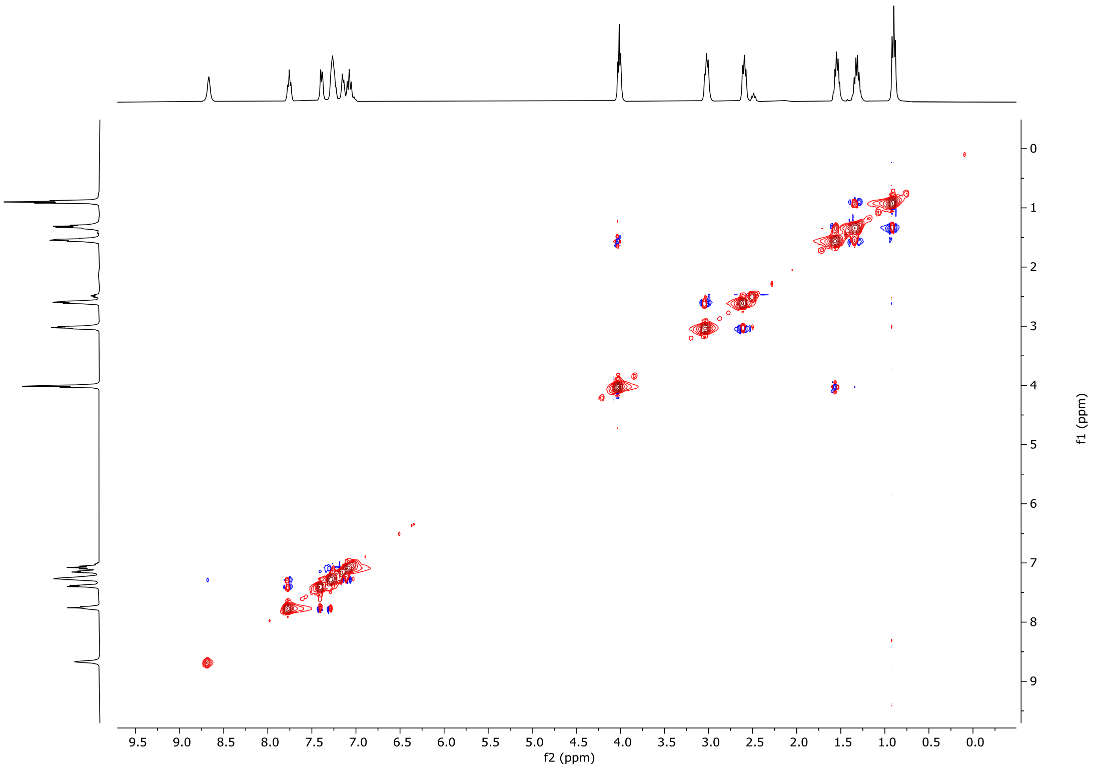

Methyl 3-(2-(pyridin-2-yl)phenyl)propanoate (3ab)

^{1}H-NMR (500 MHz, $CDCl_3$)

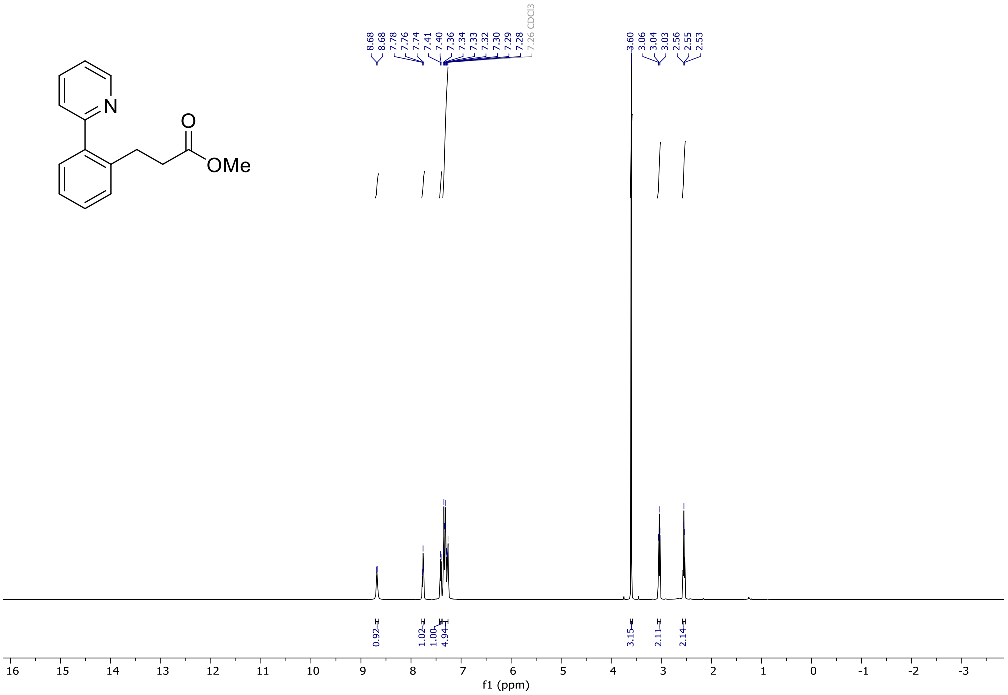

^{13}C-NMR (126 MHz, $CDCl_3$)

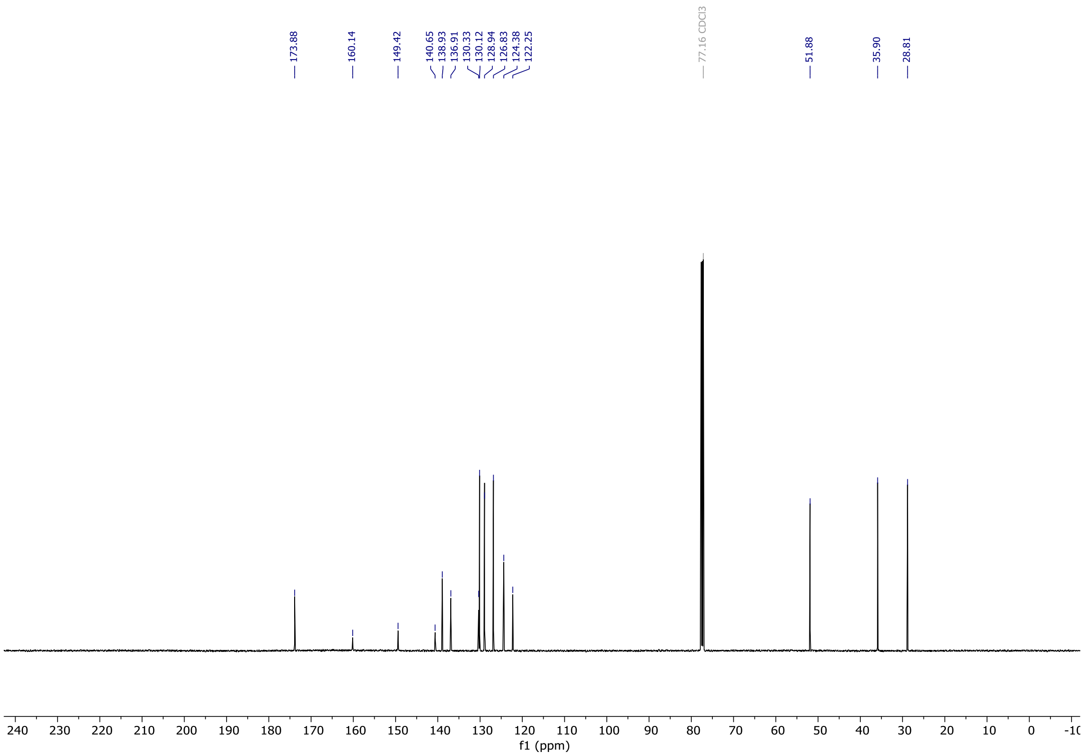

t-Butyl 3-(2-(pyridin-2-yl)phenyl)propanoate (3ac)

^{1}H-NMR (500 MHz, $CDCl_3$)

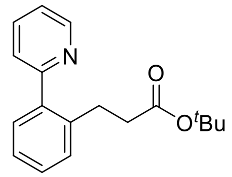

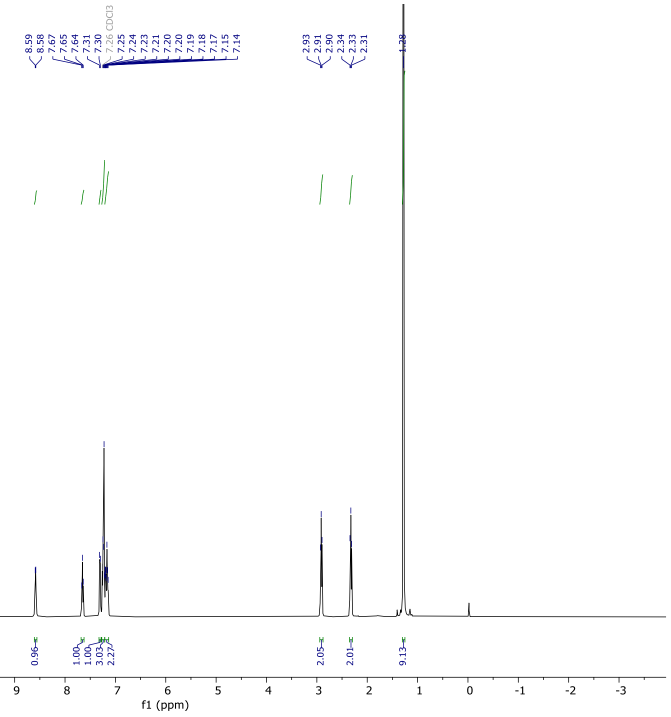

^{13}C-NMR (126 MHz, $CDCl_3$)

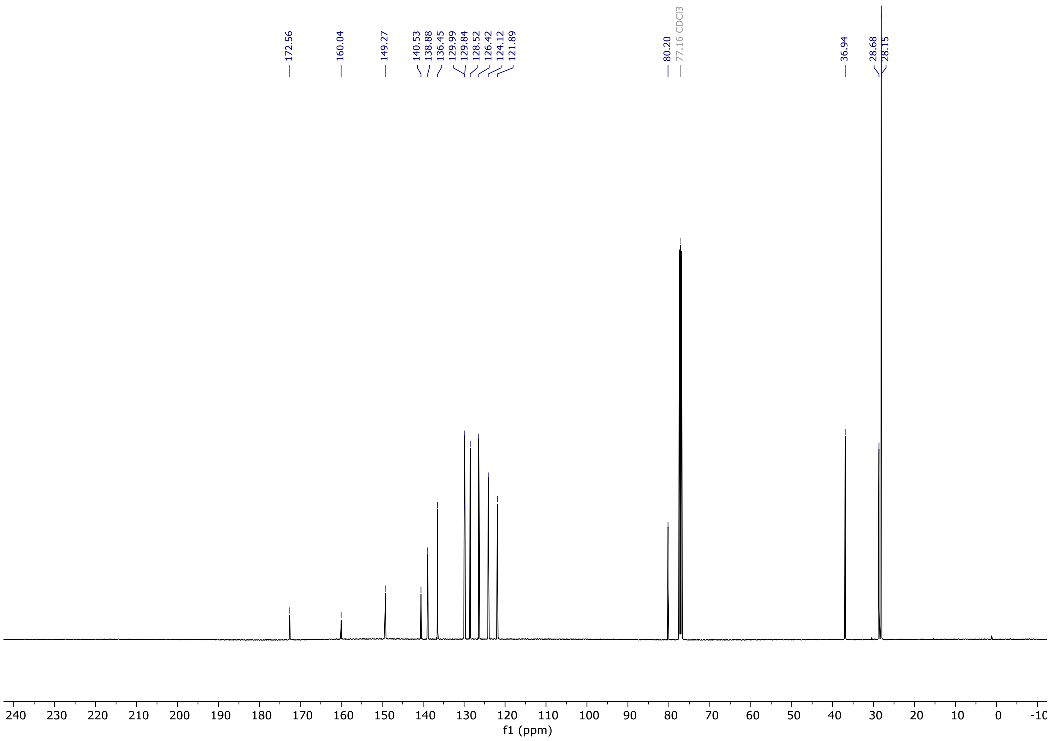

4-(2-(pyridin-2-yl)phenyl)butan-2-one (3ad)

^{1}H-NMR (400 MHz, $CDCl_3$)

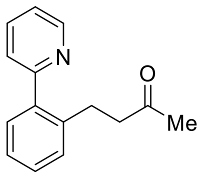

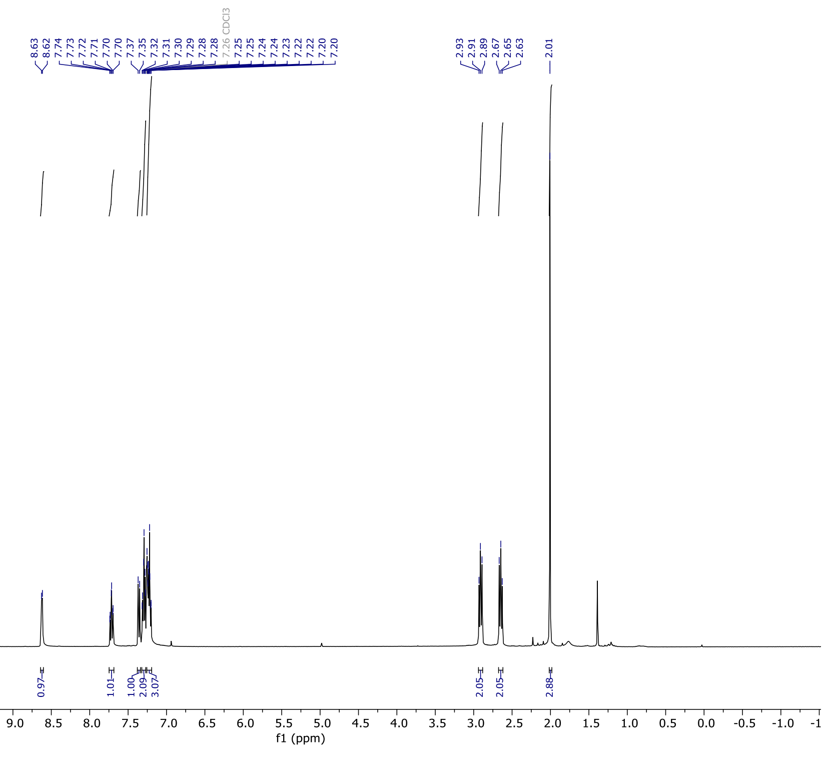

^{13}C-NMR (101 MHz, $CDCl_3$)

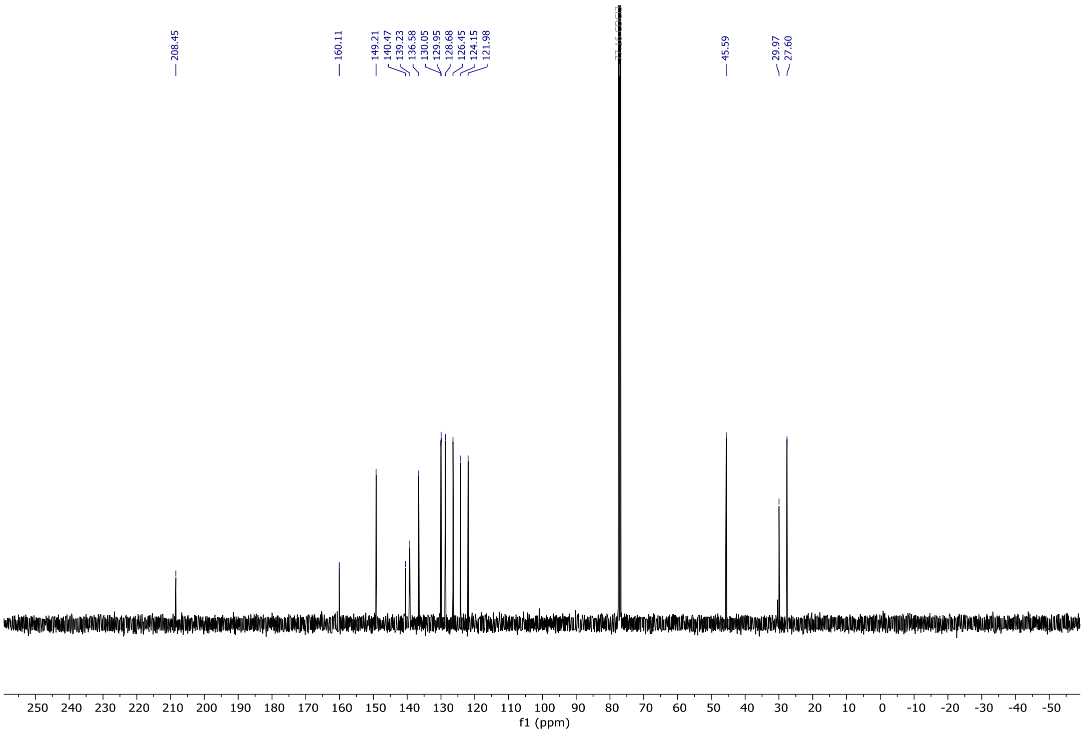

3-(2-(Pyridin-2-yl)phenyl)cyclohexanone (3ae)

^{1}H-NMR (400 MHz, $CDCl_3$)

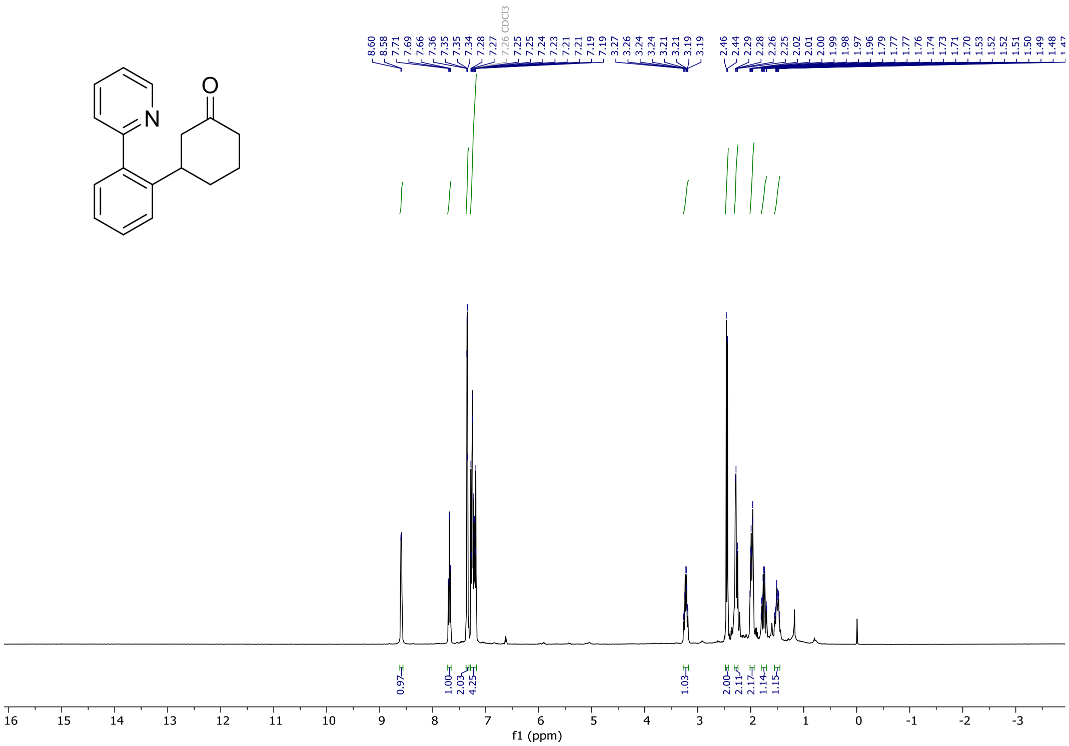

^{13}C-NMR (101 MHz, $CDCl_3$)

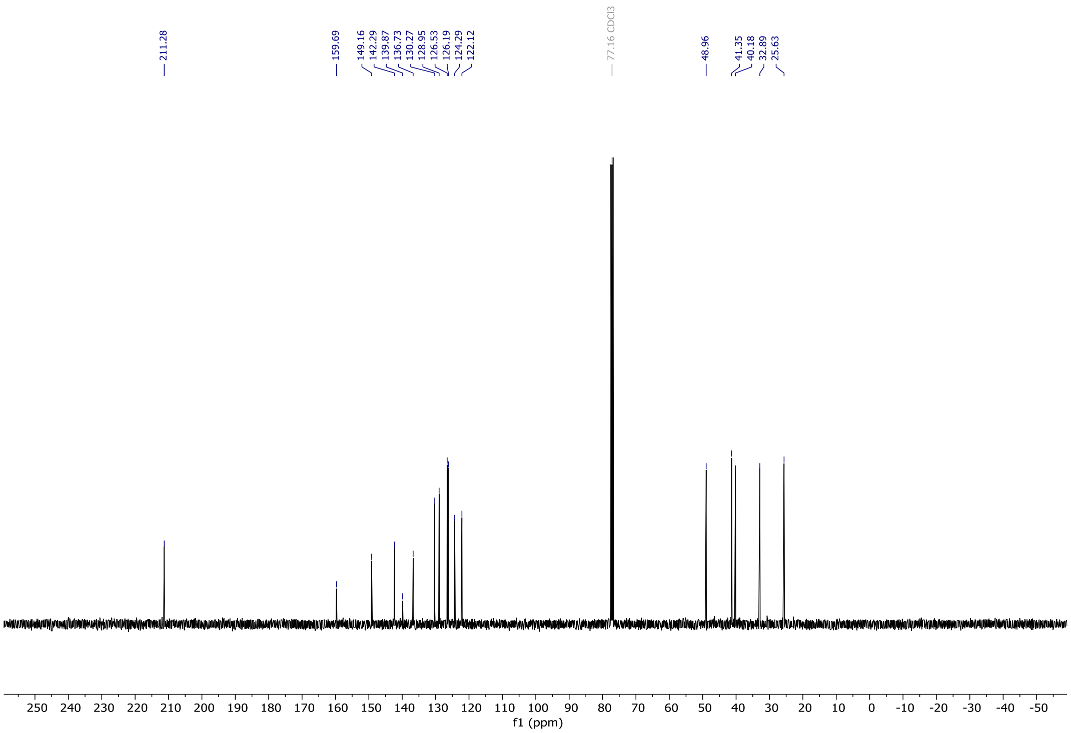

1,3-diphenyl-3-(2-(pyridin-2-yl)phenyl)propan-1-one (3af)

^{1}H-NMR (400 MHz, $CDCl_3$)

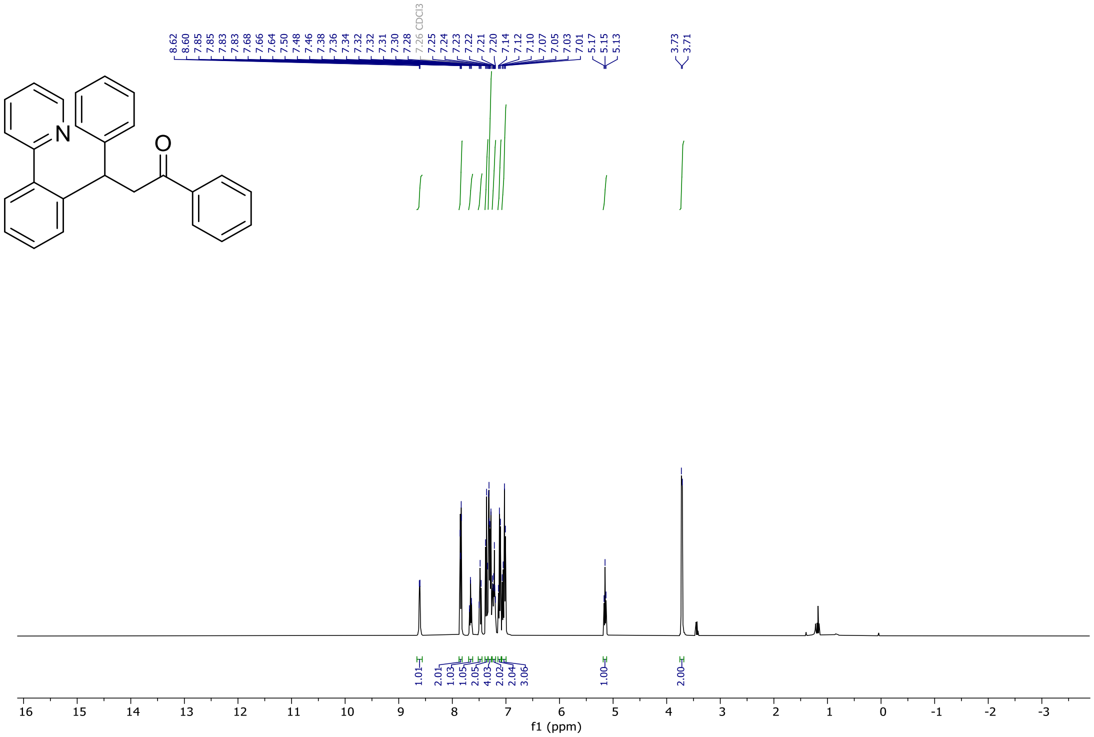

^{13}C-NMR (101 MHz, $CDCl_3$)

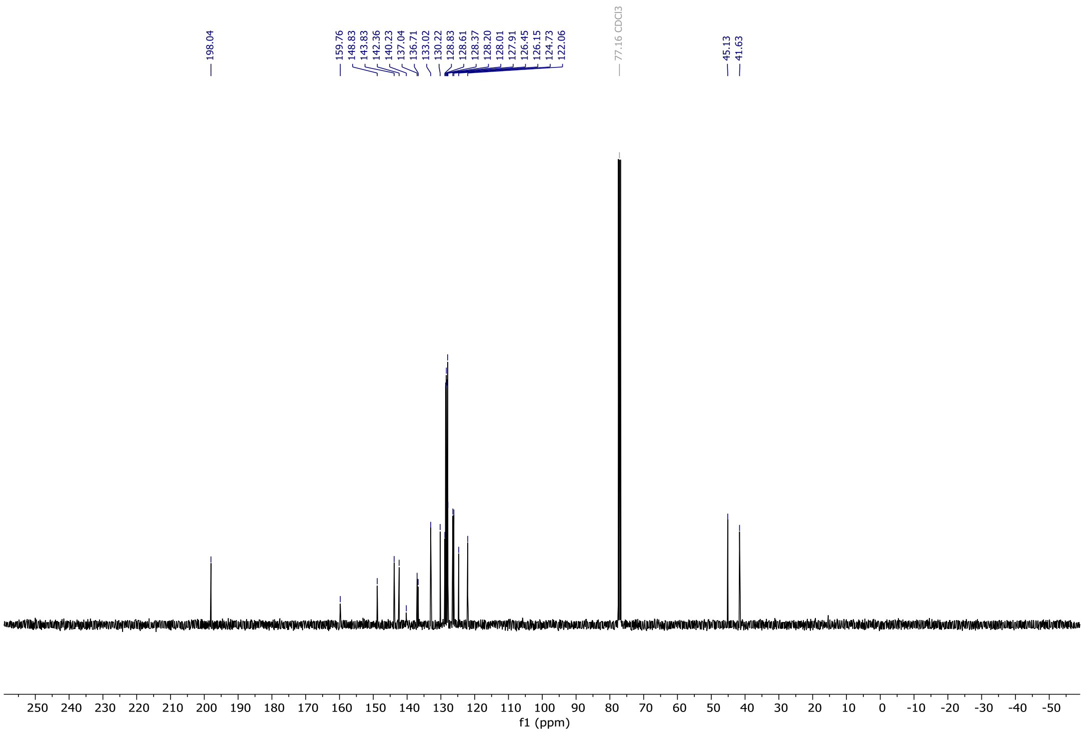

4-acetyl-2-methoxyphenyl 3-(2-(pyridin-2-yl)phenyl)propanoate (3ag)

^{1}H-NMR (400 MHz, $CDCl_3$)

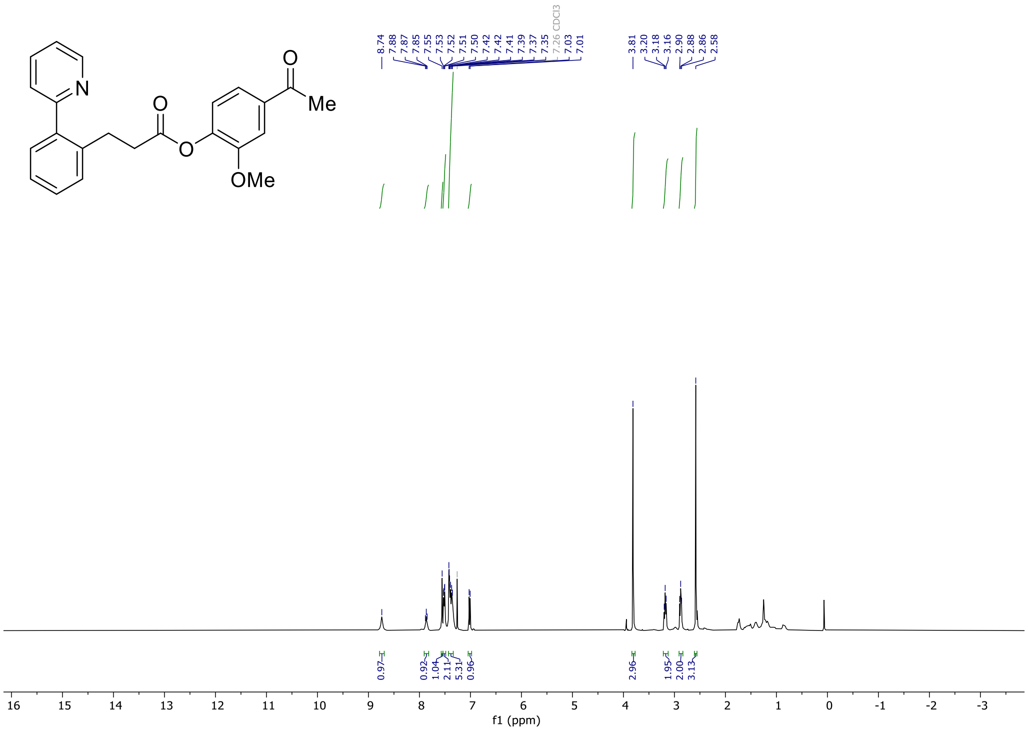

^{13}C-NMR (126 MHz, $CDCl_3$)

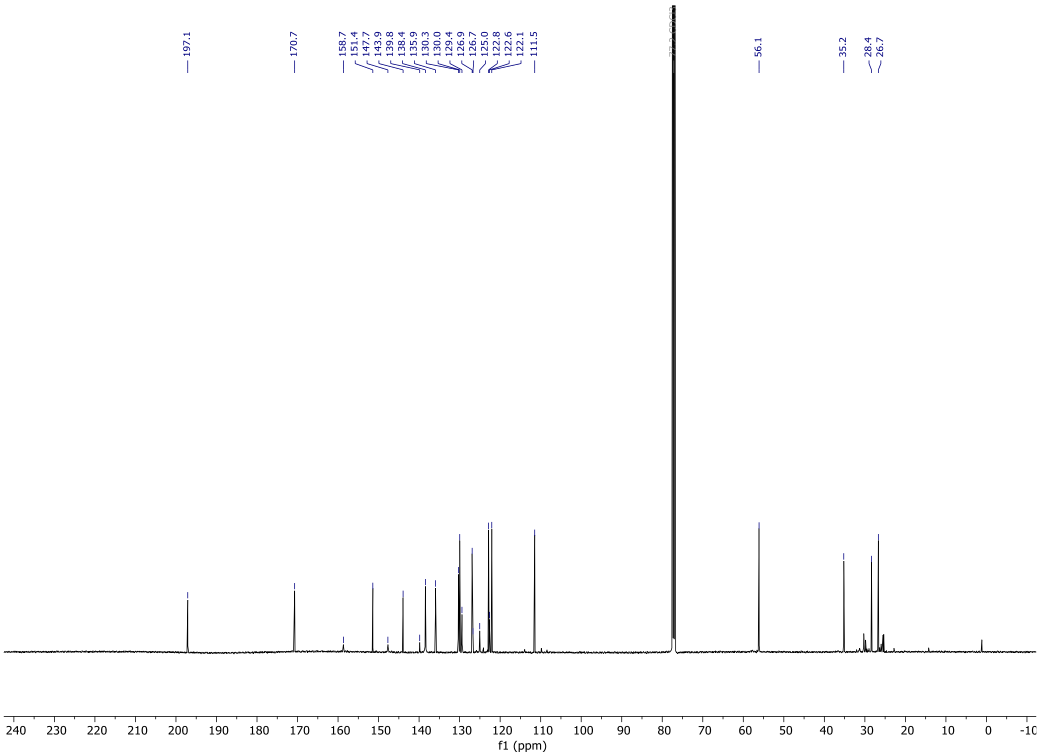

(3*S*,8*S*,9*S*,10*R*,13*R*,14*S*,17*R*)-10,13-dimethyl-17-((*R*)-6-methylheptan-2-yl)-2,3,4,7,8,9,10,11,12,13,14,15,16,17-tetradecahydro-1H-cyclopenta[a]phenanthren-3-yl 3-(2-(pyridin-2-yl)phenyl)propanoate (3ah)

^{1}H-NMR (400 MHz, $CDCl_3$)

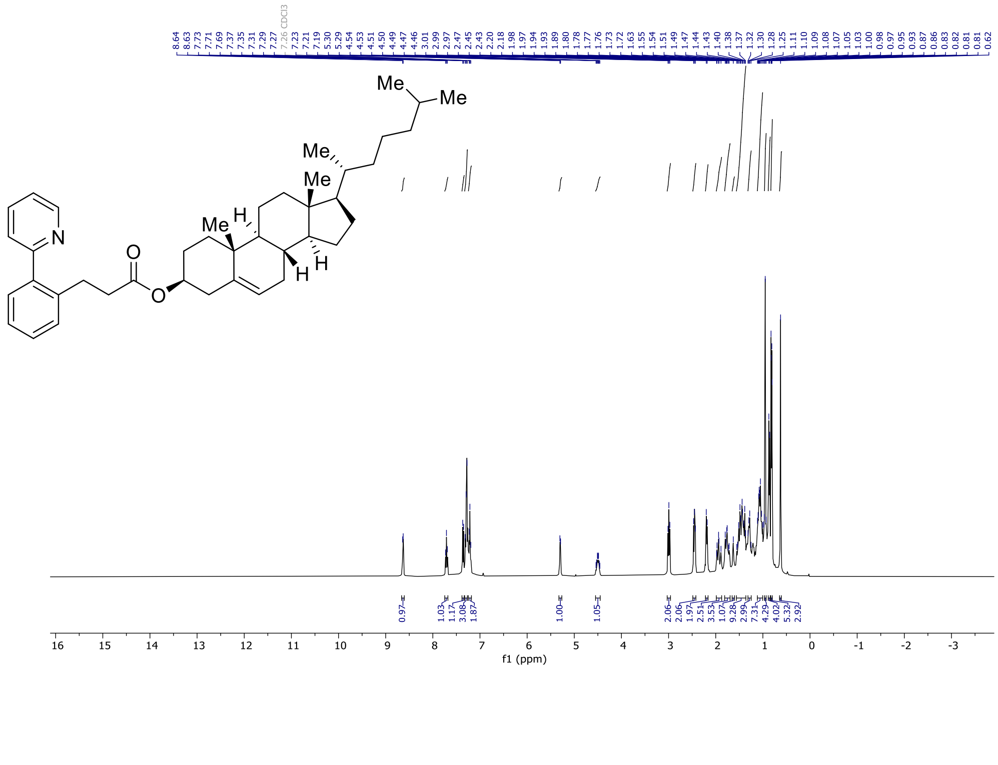

^{13}C-NMR (101 MHz, $CDCl_3$)

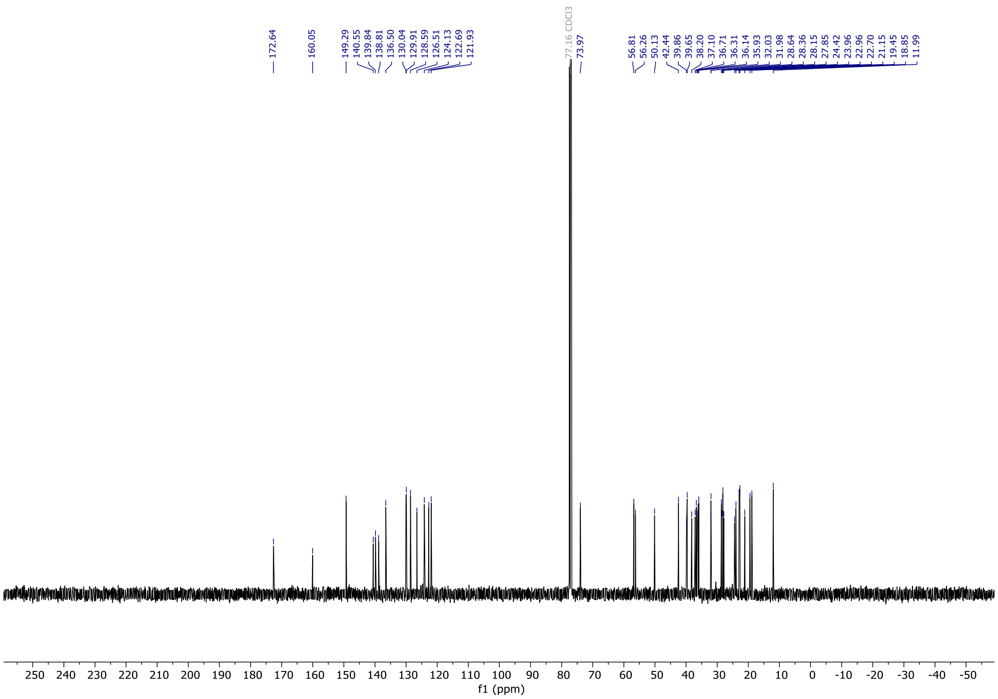

Methyl(*S*)-2-((tert-butoxycarbonyl)amino)-3-(4-((3-(2-(pyridin-2 yl)phenyl)propanoyl)oxy)phenyl)propanoate (3ai)

^{1}H NMR (400 MHz, $CDCl_3$)

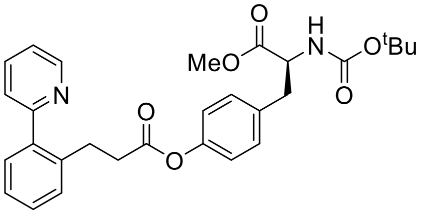

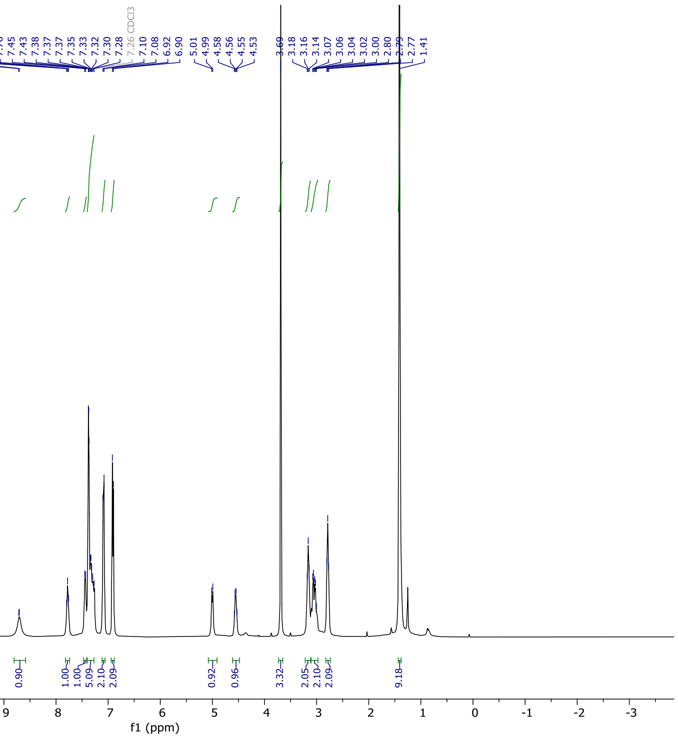

^{13}C NMR (101 MHz, $CDCl_3$)

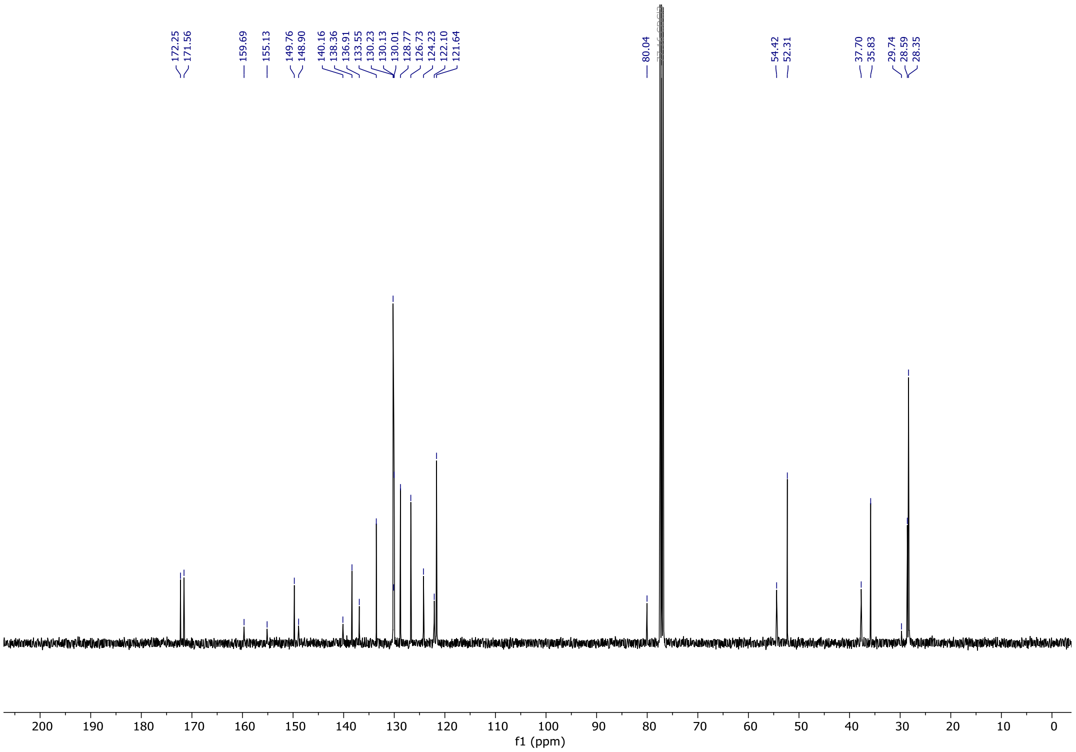

(*E*)-2-(2-(hex-1-en-1-yl)phenyl)pyridine (5aa)

^{1}H-NMR (400 MHz, $CDCl_3$)

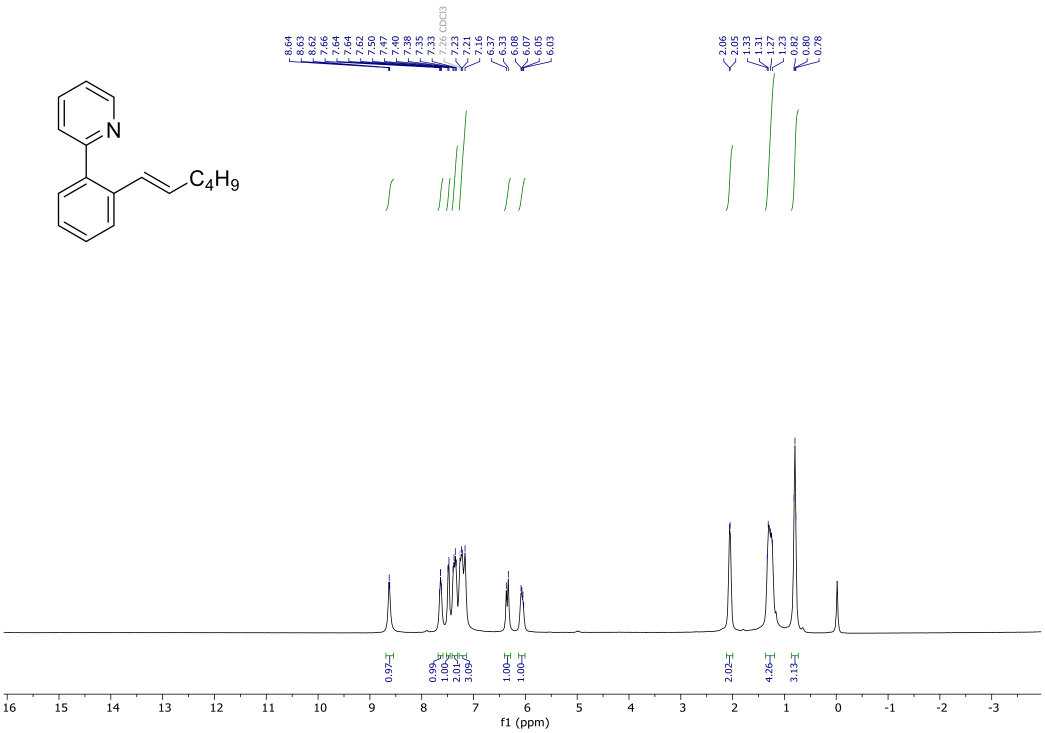

^{13}C-NMR (101 MHz, $CDCl_3$)

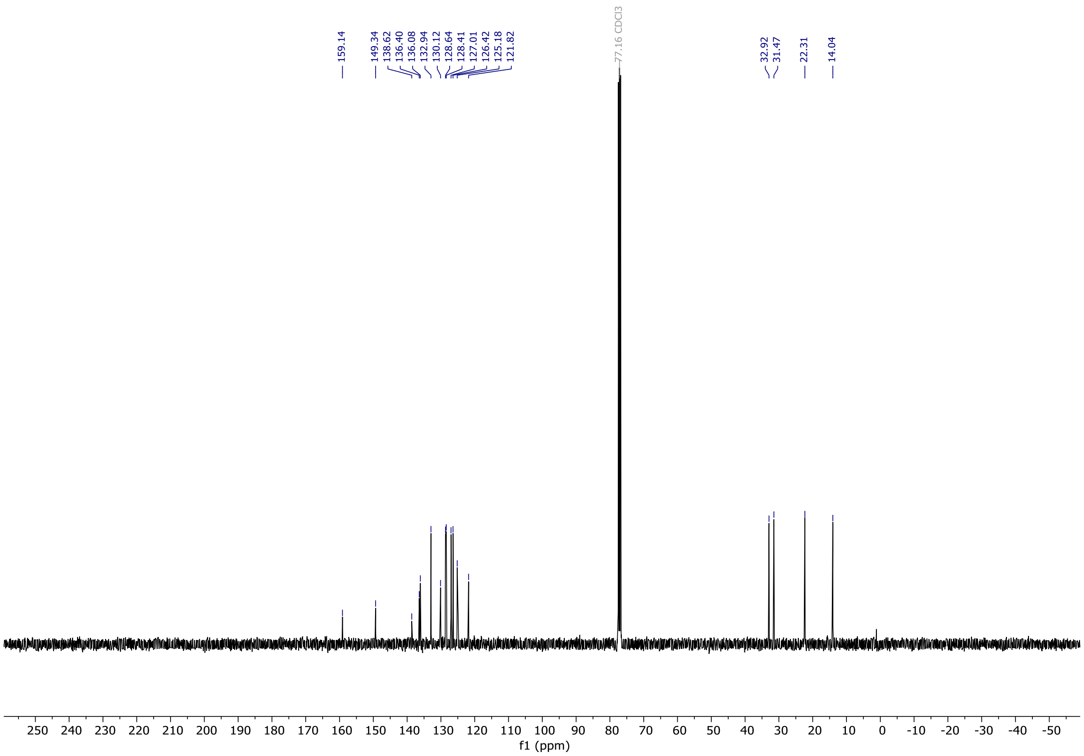

(*E*)-2-(2-(dec-1-en-1-yl)phenyl)pyridine (5ab)

^{1}H-NMR (400 MHz, $CDCl_3$)

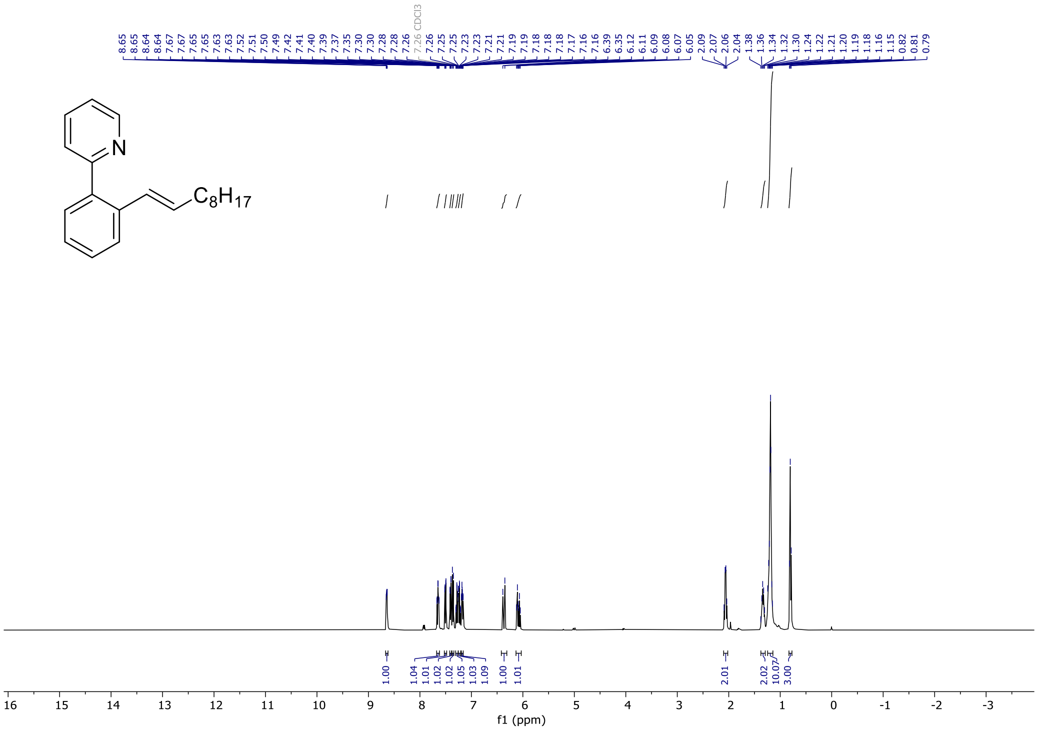

^{13}C-NMR (101 MHz, CDCl$_3$)

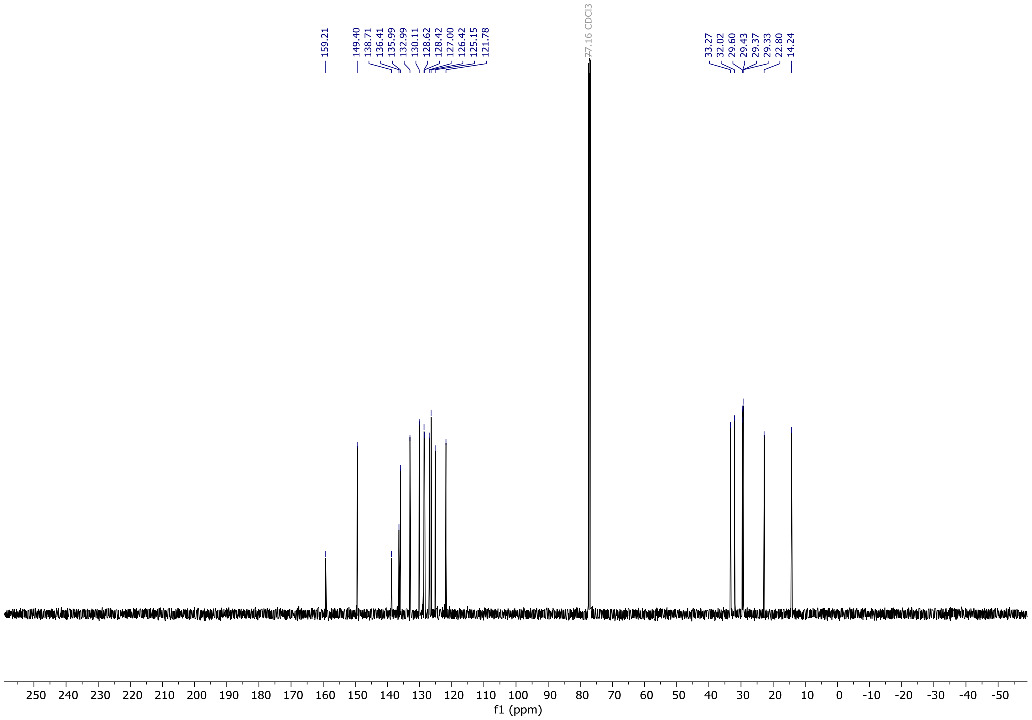

(*E*)-2-(2-(4-methoxystyryl)phenyl)pyridine (5ac)

^{1}H-NMR (400 MHz, CDCl$_3$)

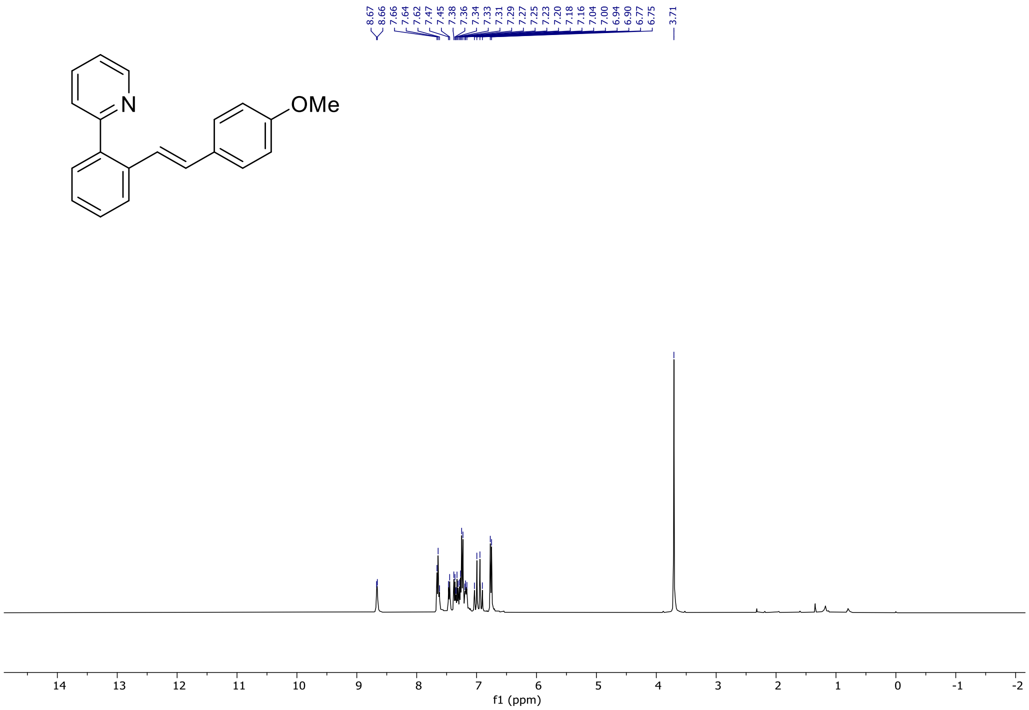

^{13}C-NMR (101 MHz, $CDCl_3$)

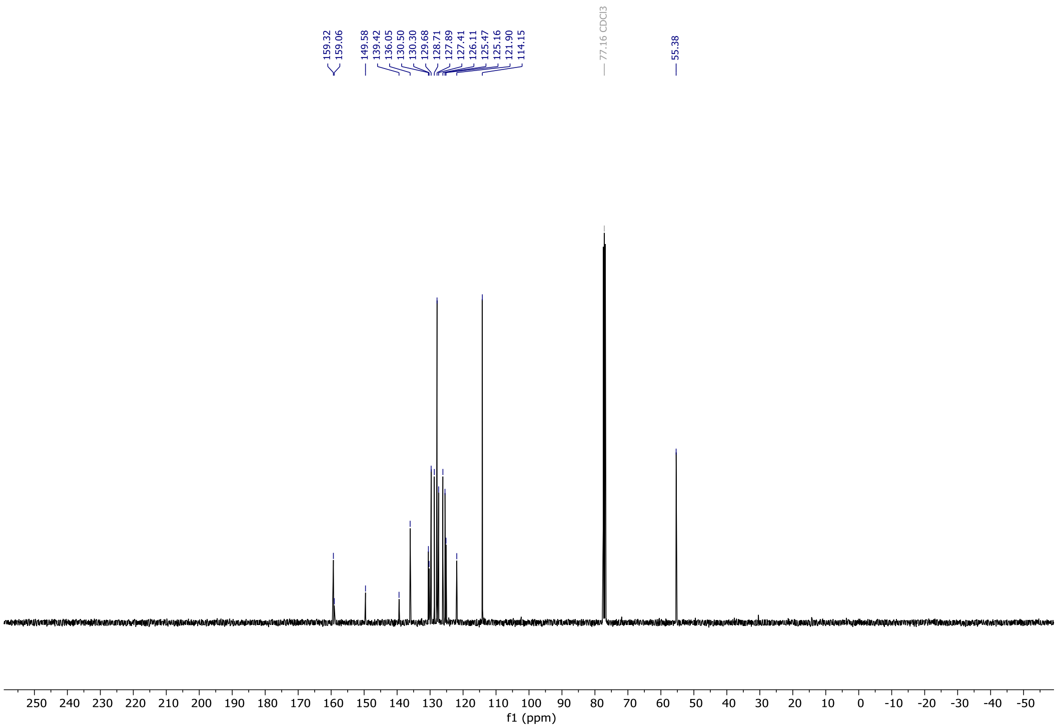

(*E*)-2-(2-styrylphenyl)pyridine (5ad)

^{1}H-NMR (500 MHz, $CDCl_3$)

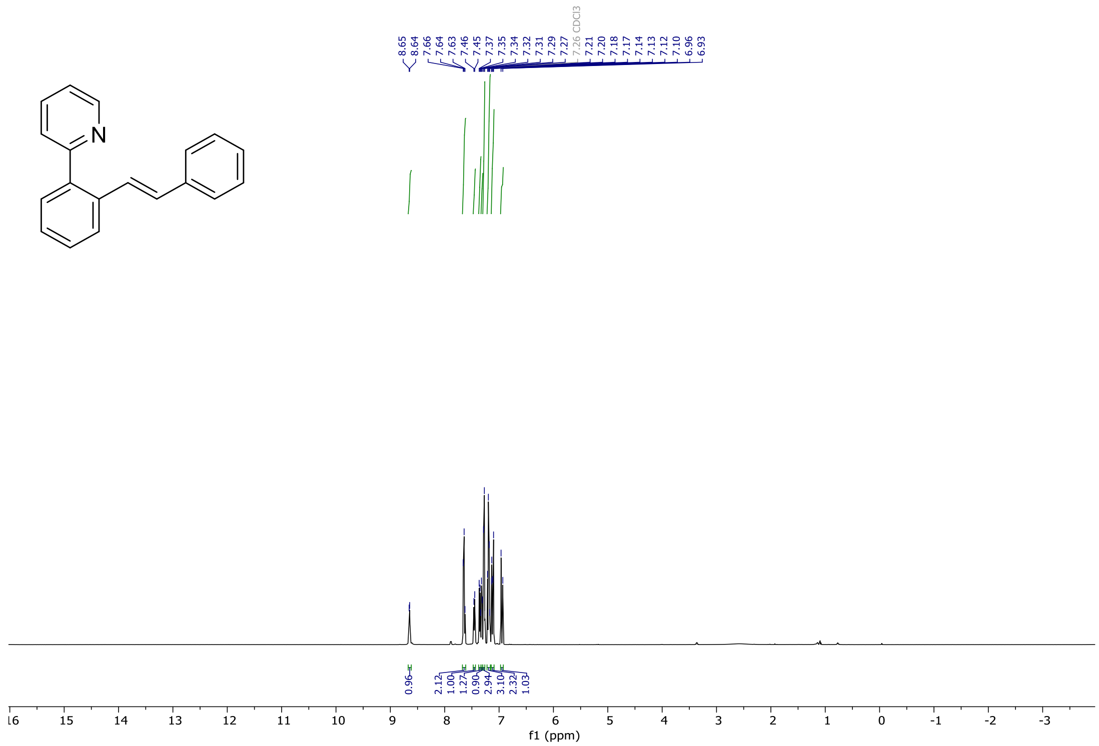

^{13}C-NMR (126 MHz, $CDCl_3$)

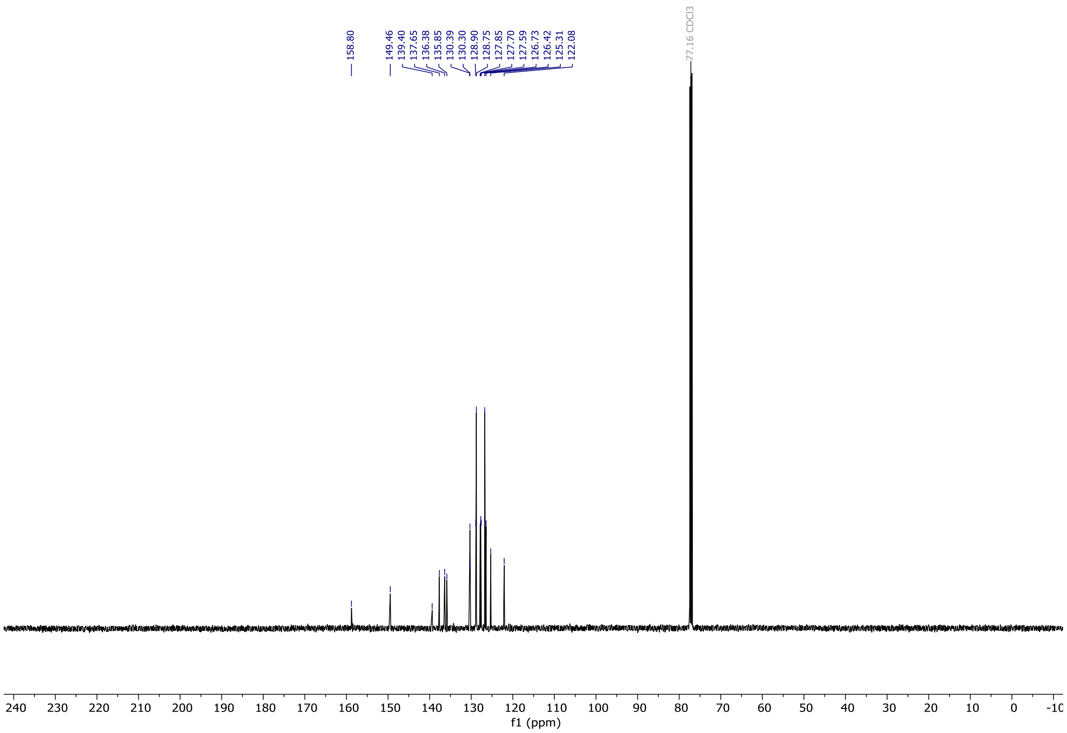

(*E*)-2-(2-(4-bromostyryl)phenyl)pyridine (5ae)

^{1}H-NMR (400 MHz, $CDCl_3$)

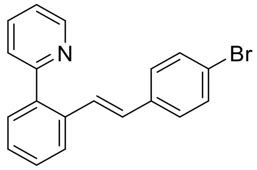

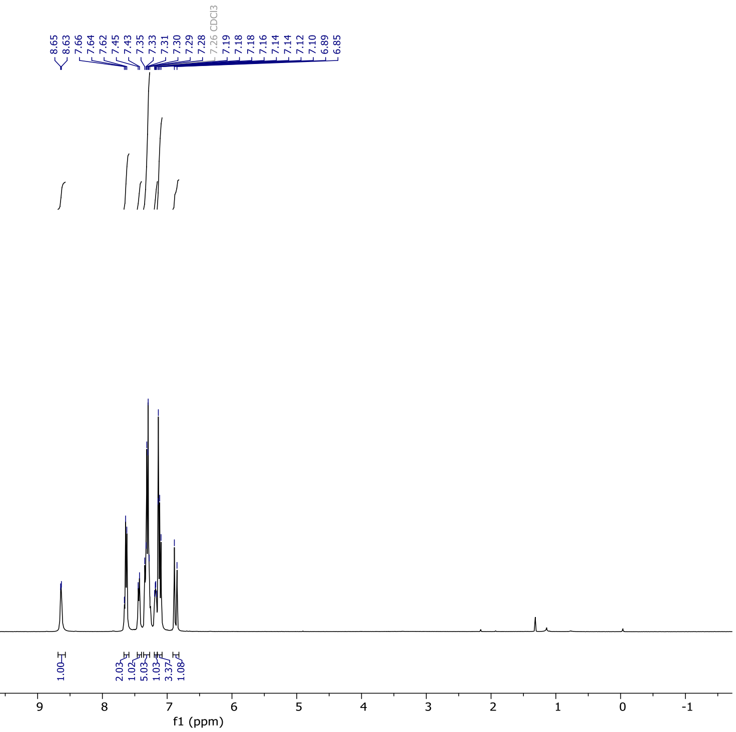

^{13}C-NMR (101 MHz, $CDCl_3$)

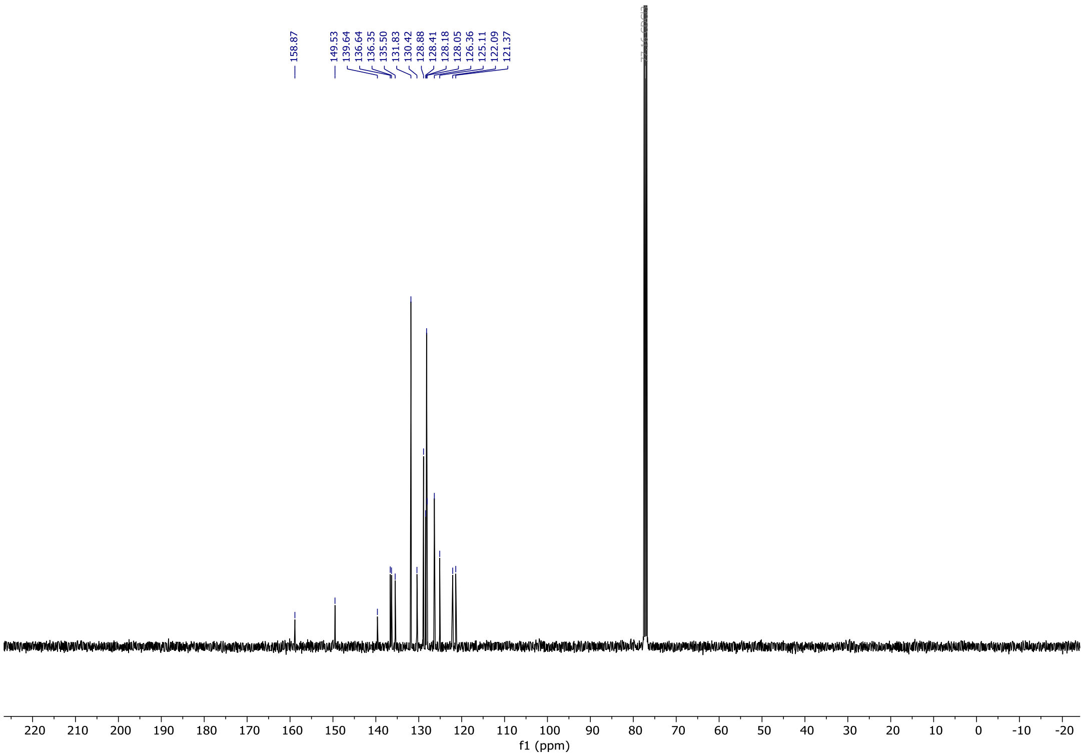

(*E*)-methyl 4-(2-(pyridin-2-yl)styryl)benzoate (5af)

^{1}H-NMR (400 MHz, $CDCl_3$)

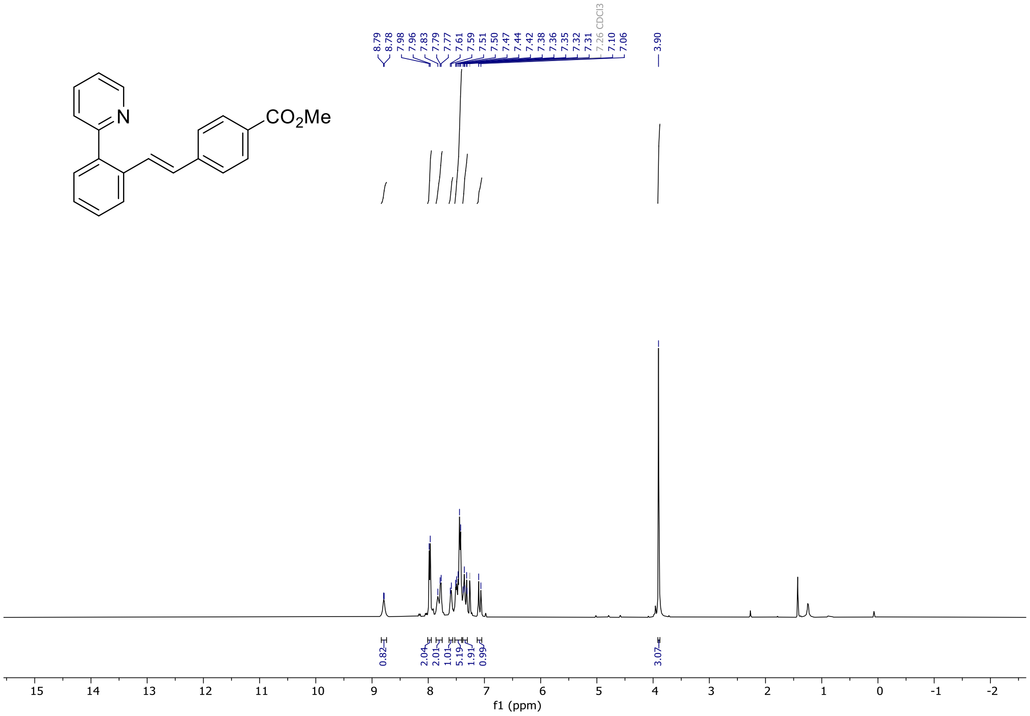

^{13}C-NMR (126 MHz, $CDCl_3$)

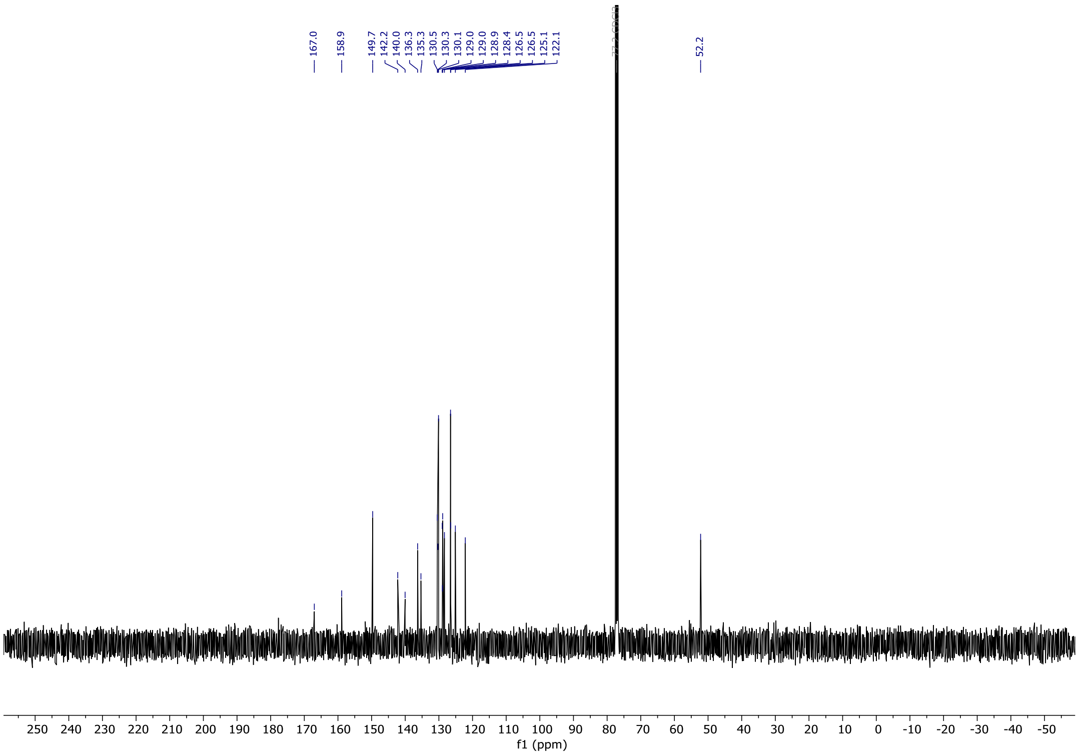

(*E*)-2-(2-(2-(naphthalen-2-yl)vinyl)phenyl)pyridine (5ag)

^{1}H-NMR (400 MHz, $CDCl_3$)

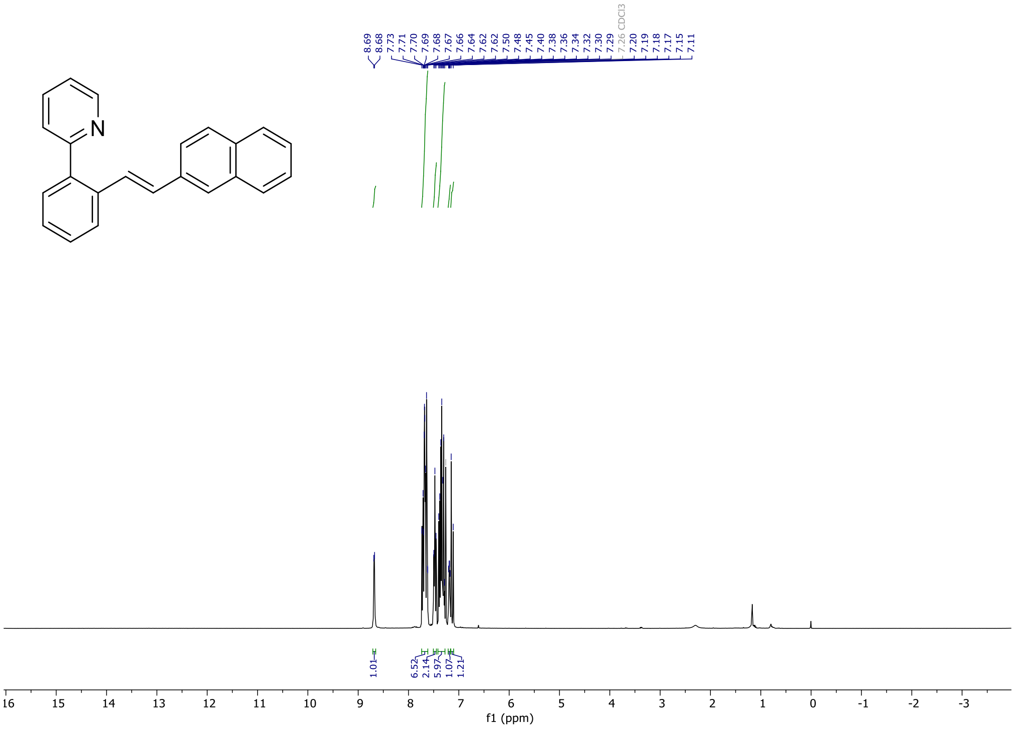

^{13}C-NMR (101 MHz, $CDCl_3$)

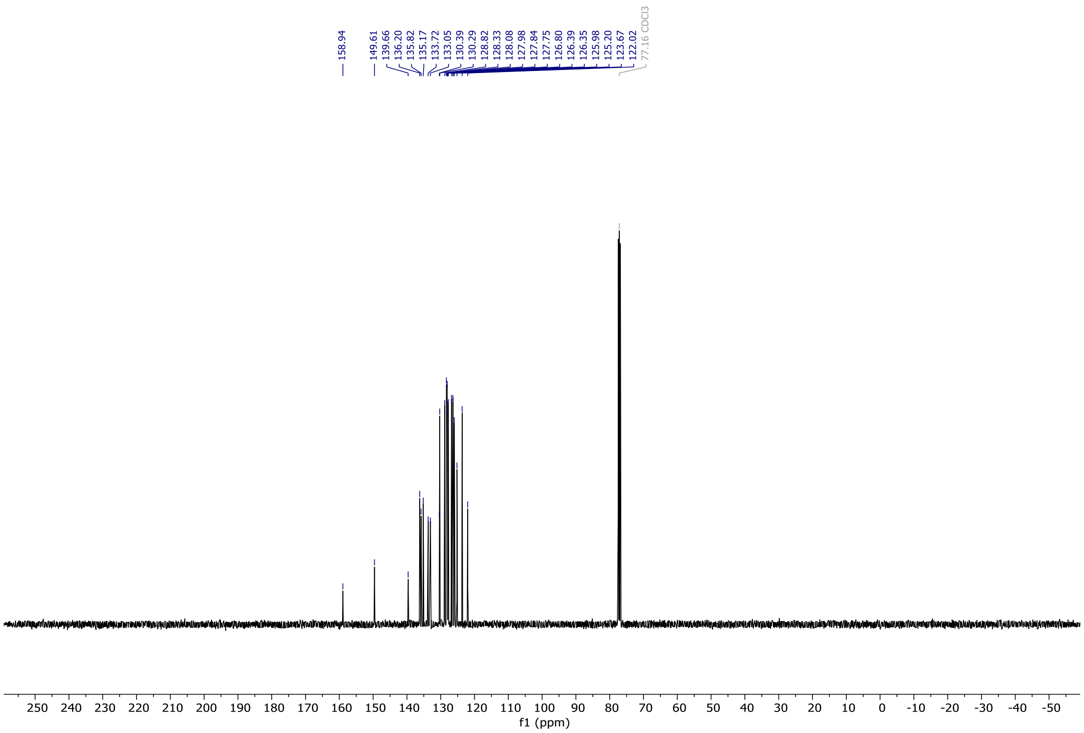

(*E*)-2-(2-(3-phenylprop-1-en-1-yl)phenyl)pyridine (5ah)

^{1}H-NMR (400 MHz, $CDCl_3$)

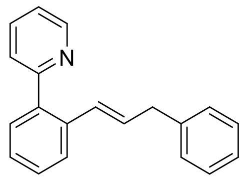

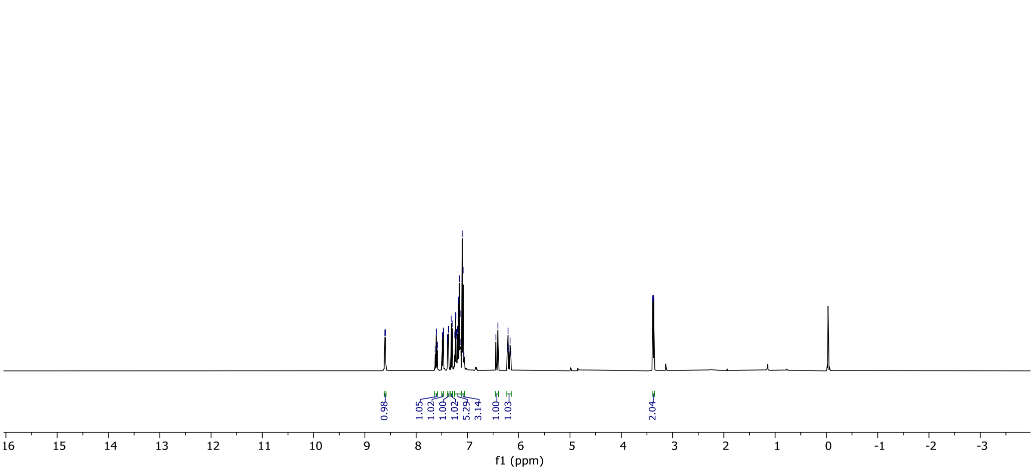

^{13}C-NMR (101 MHz, CDCl$_3$)

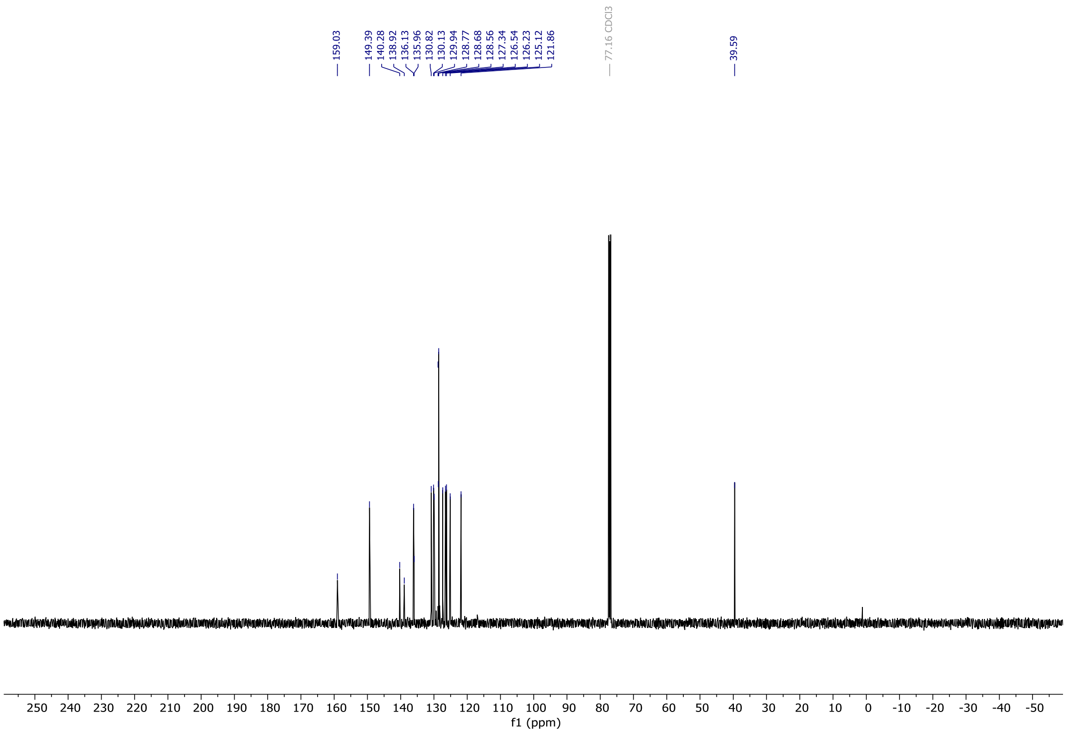

(*E*)-2-(2-(4-Phenylbut-1-en-1-yl)phenyl)pyridine (5ai)

^{1}H-NMR (500 MHz, CDCl$_3$)

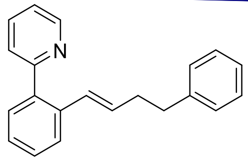

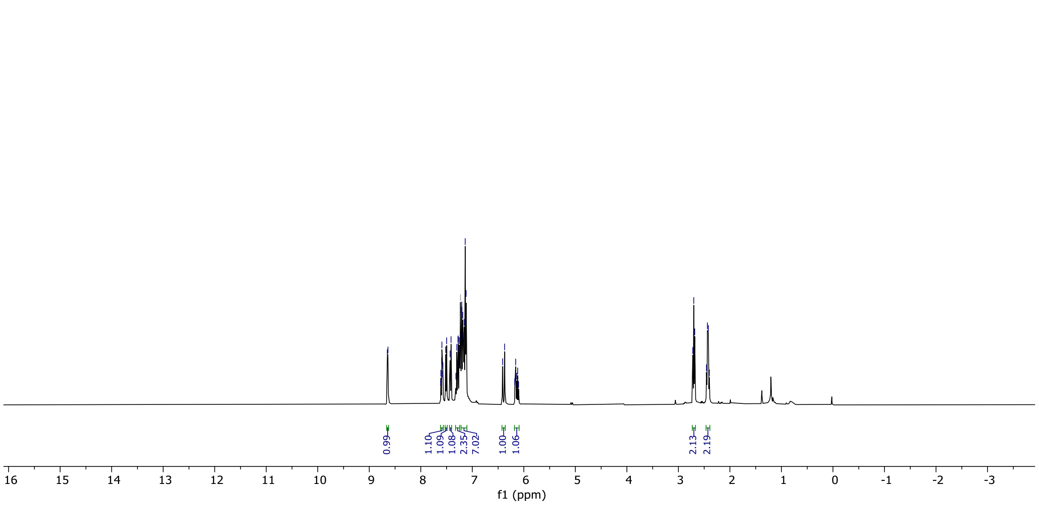

^{13}C-NMR (126 MHz, $CDCl_3$)

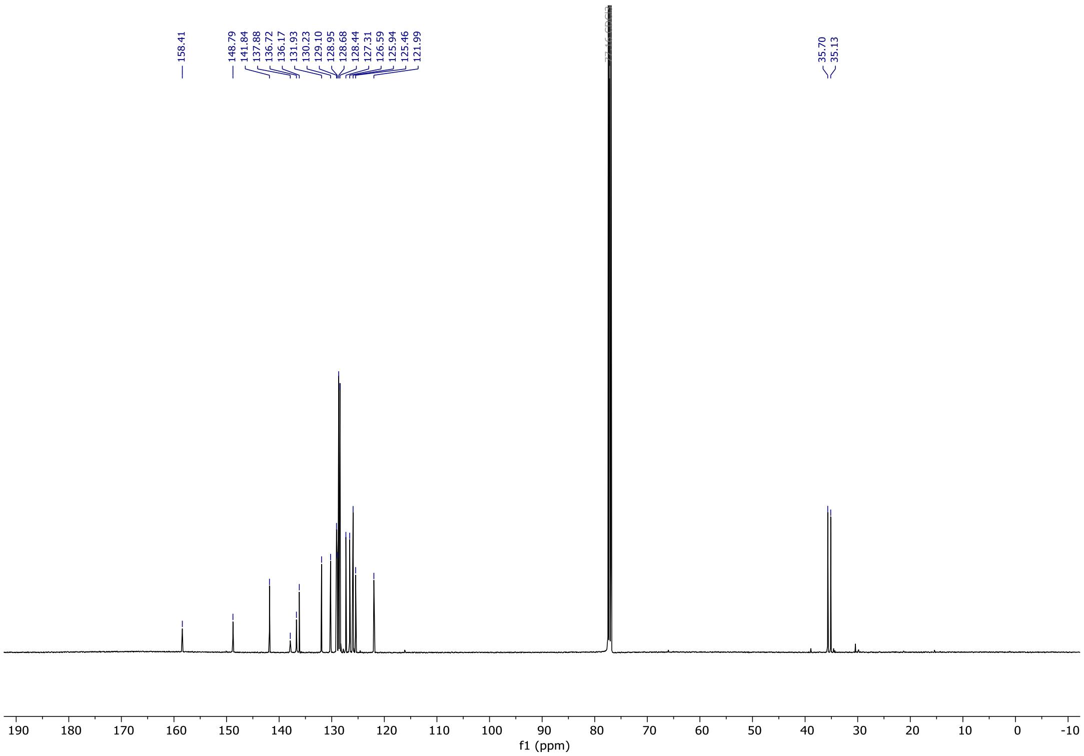

Methyl (*E*)-(4-(2-(pyridin-2-yl)styryl)benzoyl)-L-alaninate (5aj)

^{1}H-NMR (400 MHz, $CDCl_3$)

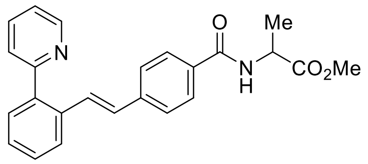

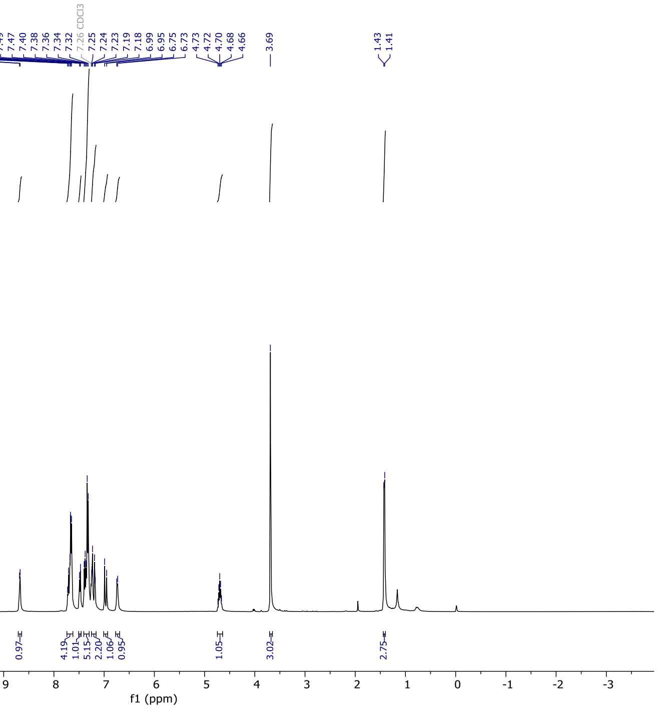

^{13}C-NMR (101 MHz, $CDCl_3$)

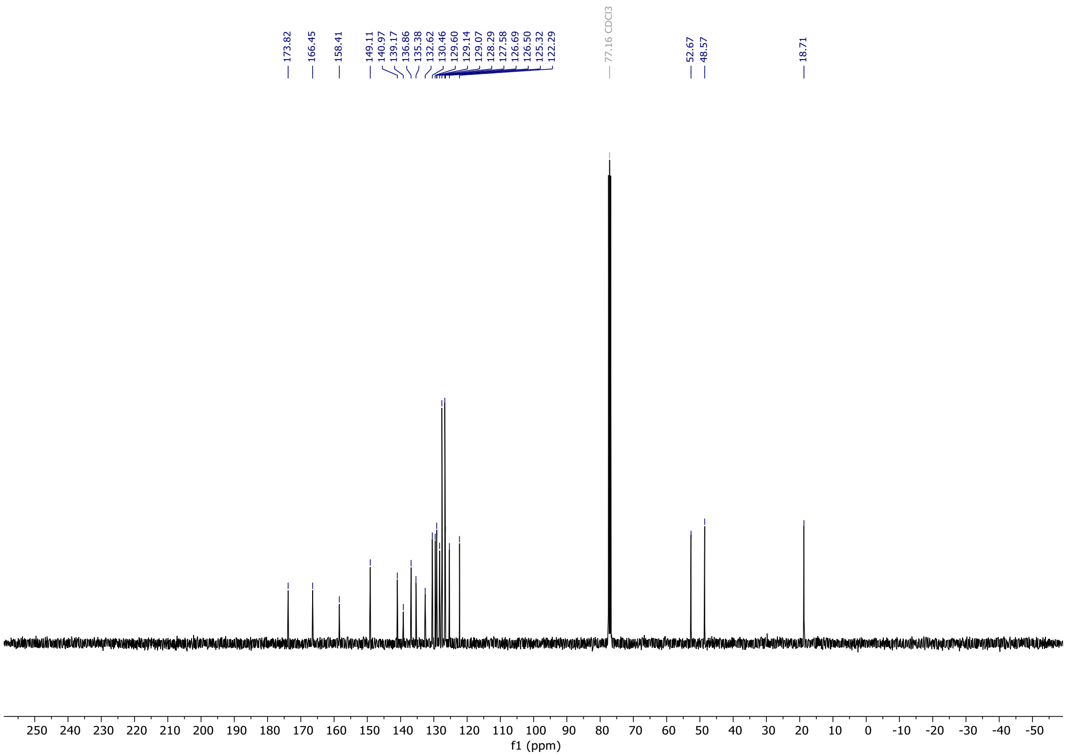

(*E*)-2-(2-(1-phenylprop-1-en-1-yl)phenyl)pyridine (5ak)

^{1}H-NMR (400 MHz, $CDCl_3$)

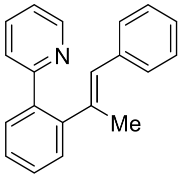

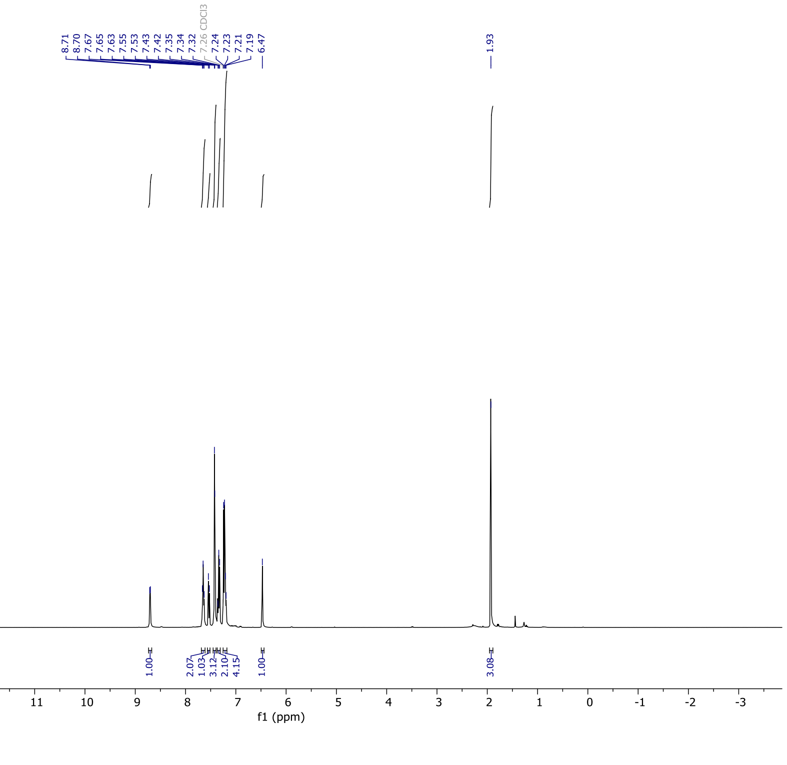

^{13}C-NMR (101 MHz, $CDCl_3$)

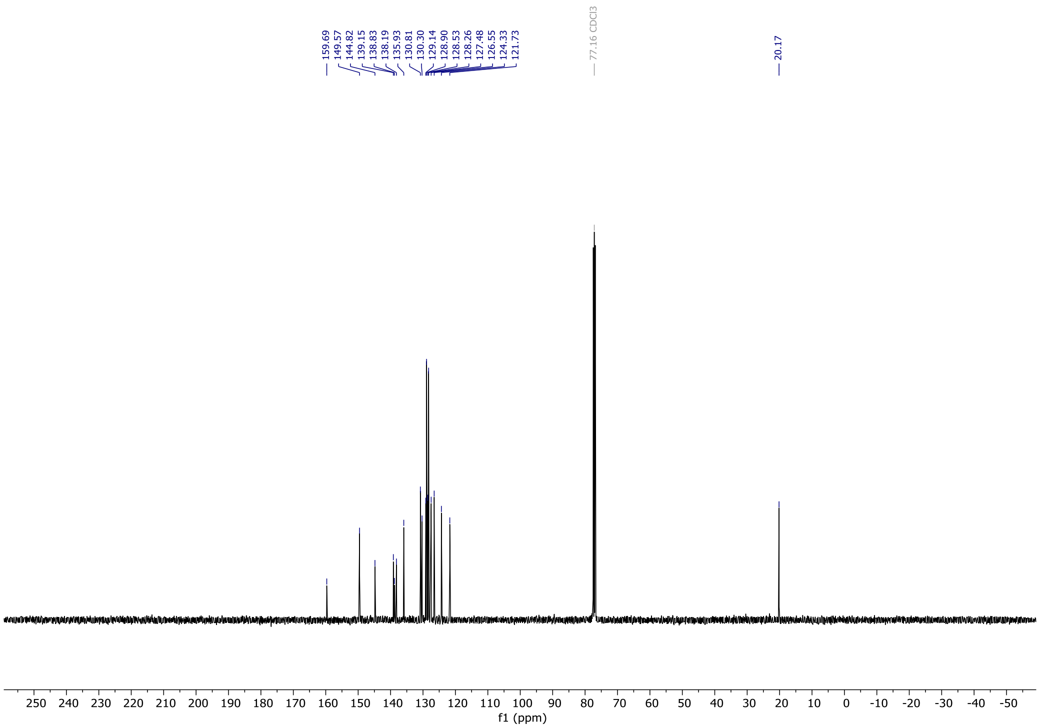

2-D NOESY-NMR (500 MHz, $CDCl_3$)

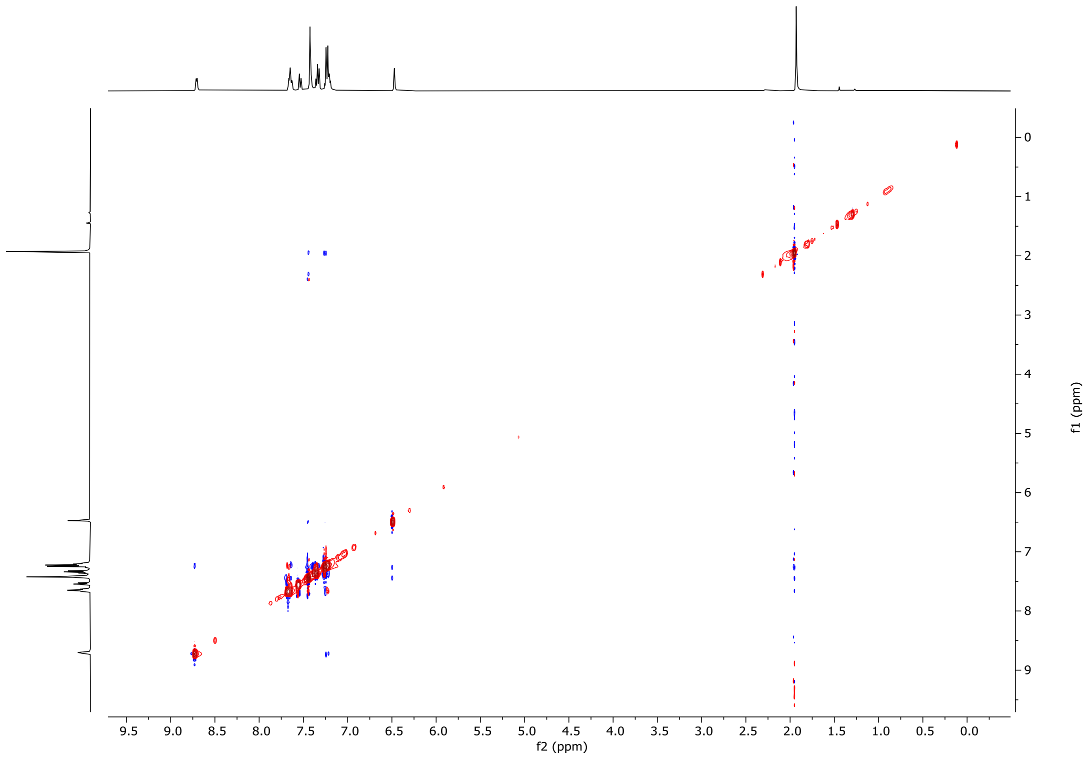

HMBC-NMR (400 MHz, $CDCl_3$)

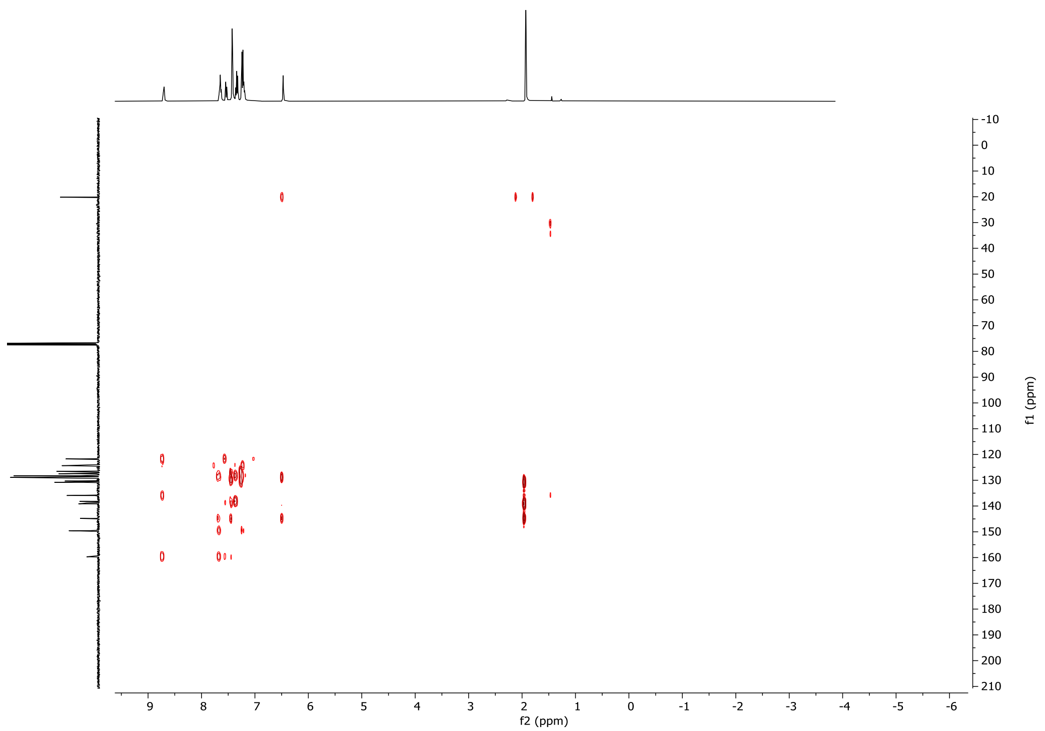

(*E*)-2-(2-(1-phenylpent-1-en-2-yl)phenyl)pyridine (5al)

^{1}H-NMR (500 MHz, $CDCl_3$)

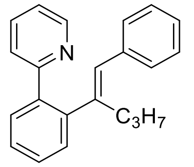

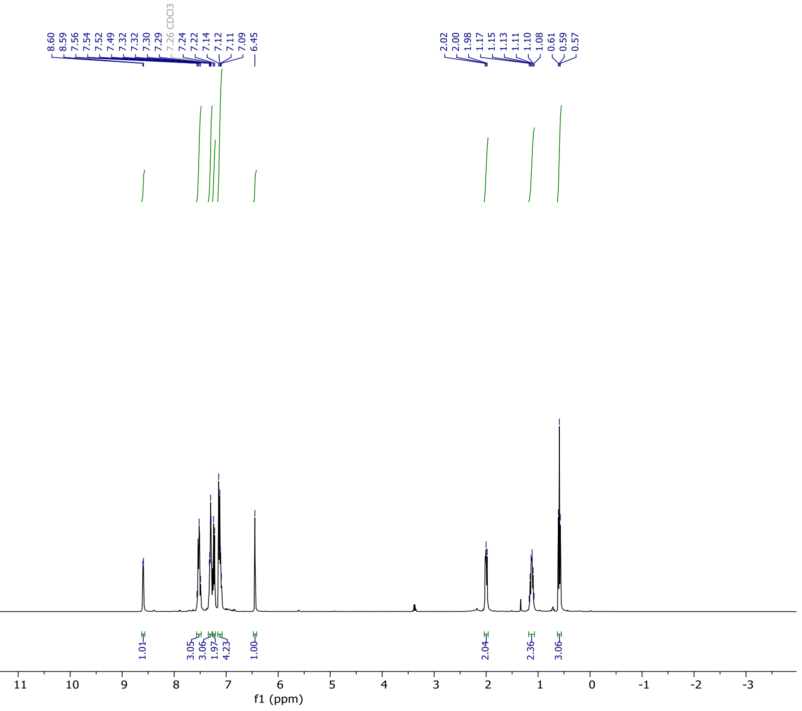

^{13}C-NMR (126 MHz, $CDCl_3$)

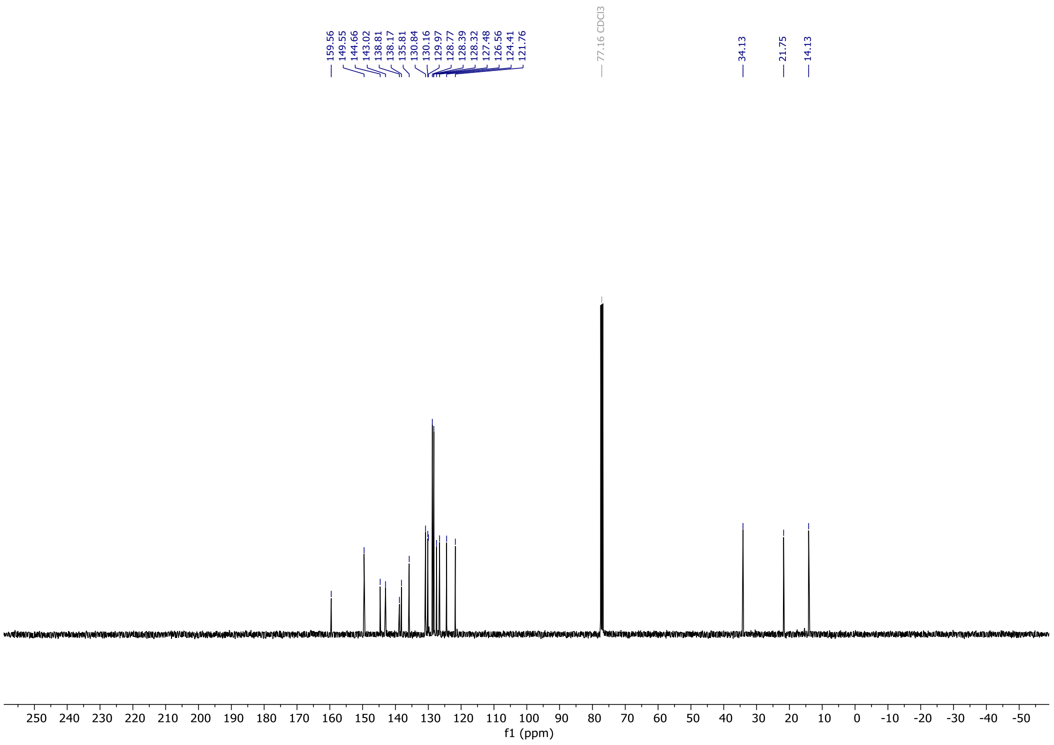

2-D NOESY-NMR (400 MHz, $CDCl_3$)

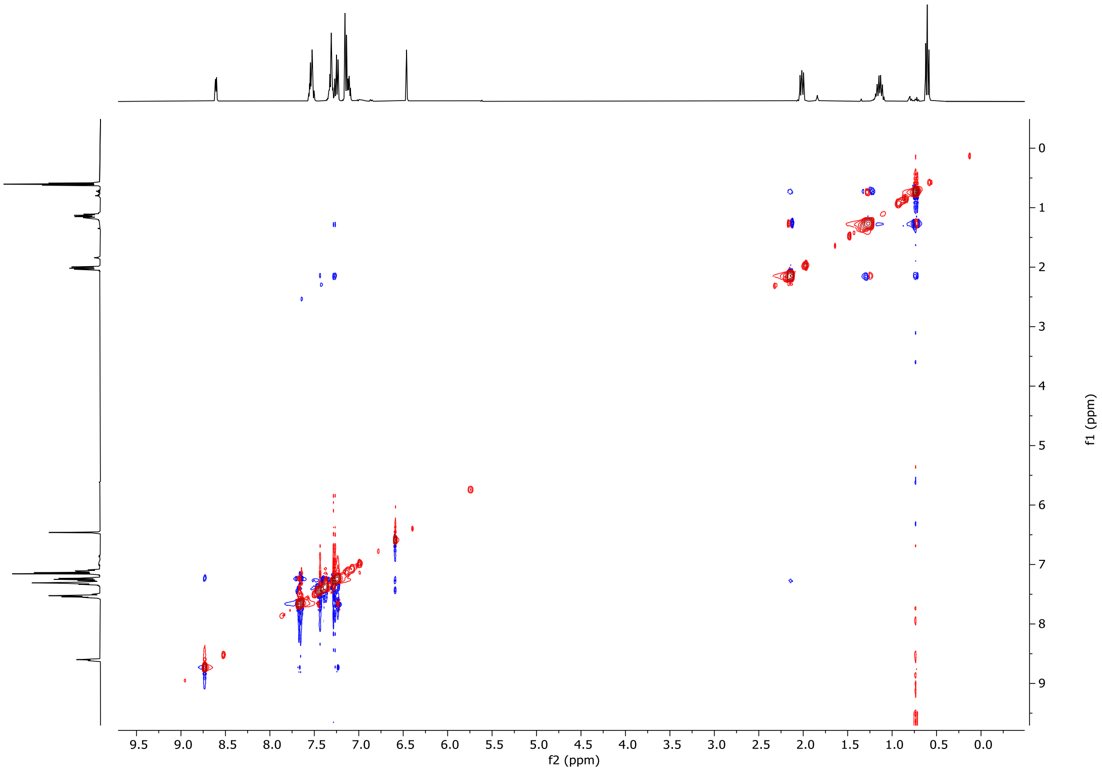

HMBC-NMR (400 MHz, $CDCl_3$)

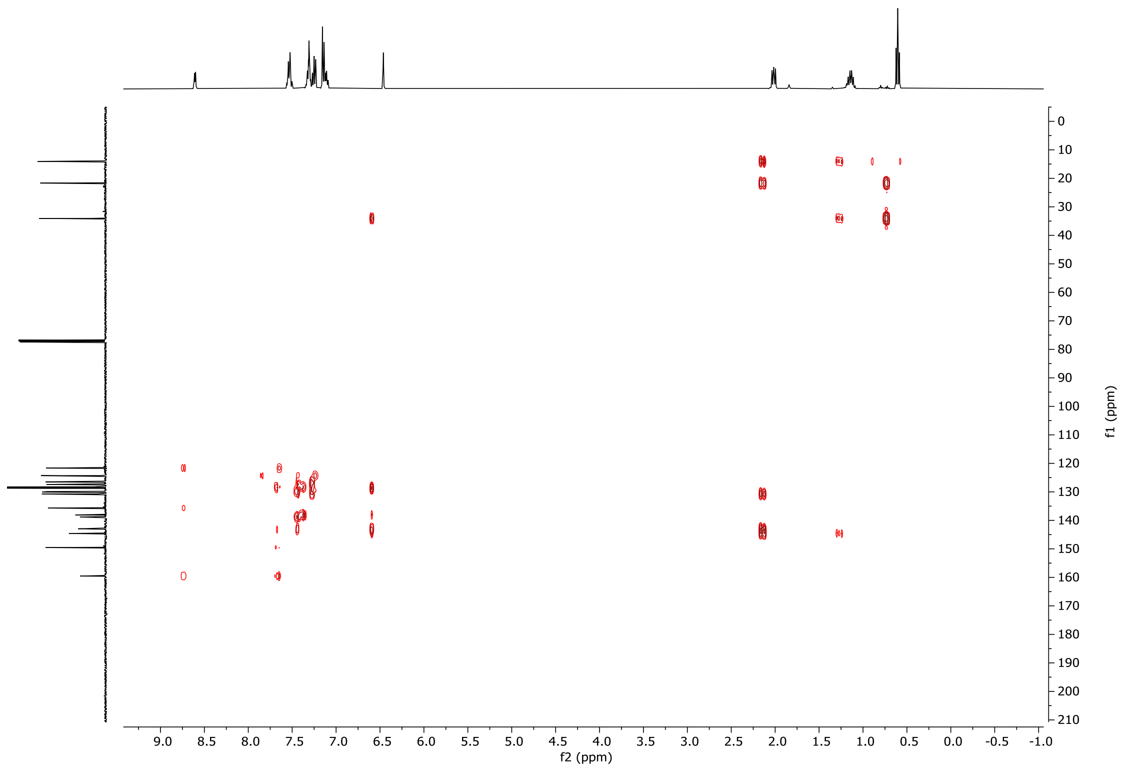

Methyl (*Z*)-3-phenyl-2-(2-(pyridin-2-yl)phenyl)acrylate (5am)

^{1}H-NMR (500 MHz, $CDCl_3$)

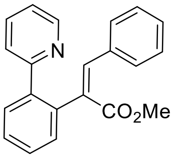

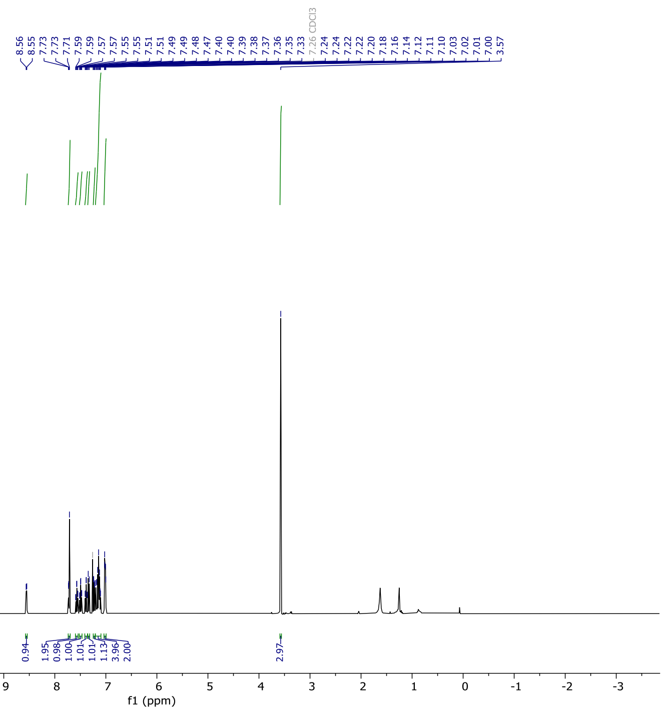

^{13}C-NMR (126 MHz, $CDCl_3$)

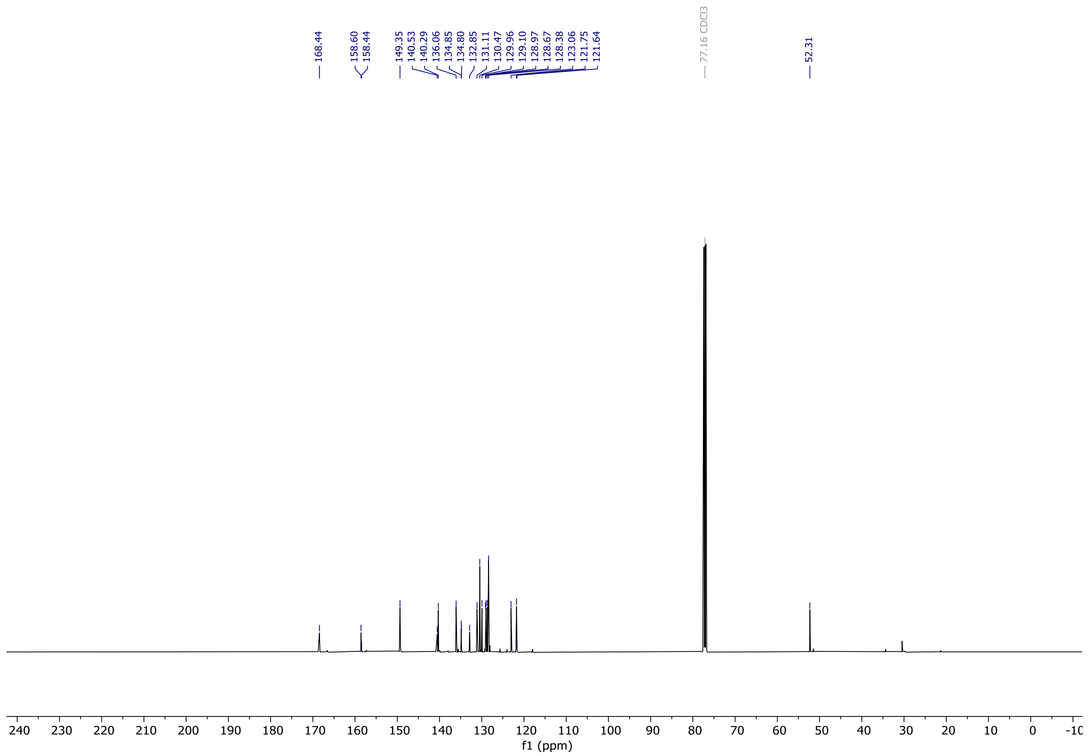

2-D NOESY-NMR (400 MHz, $CDCl_3$)

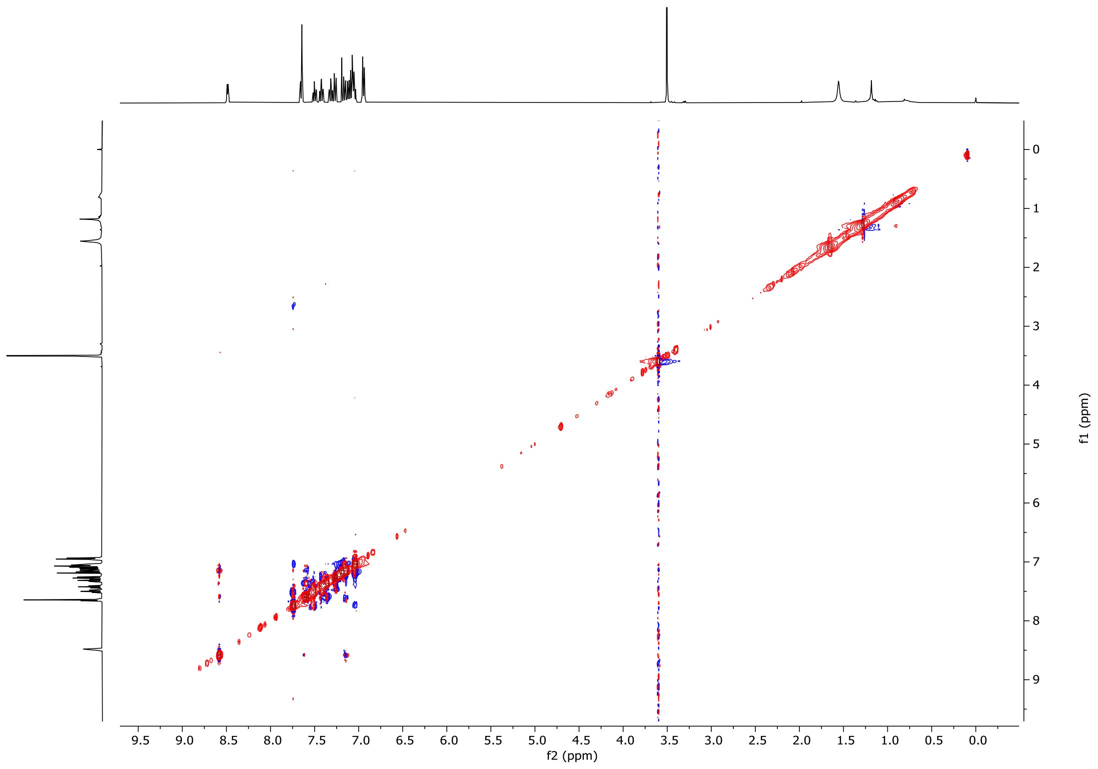

HMBC-NMR (400 MHz, $CDCl_3$)

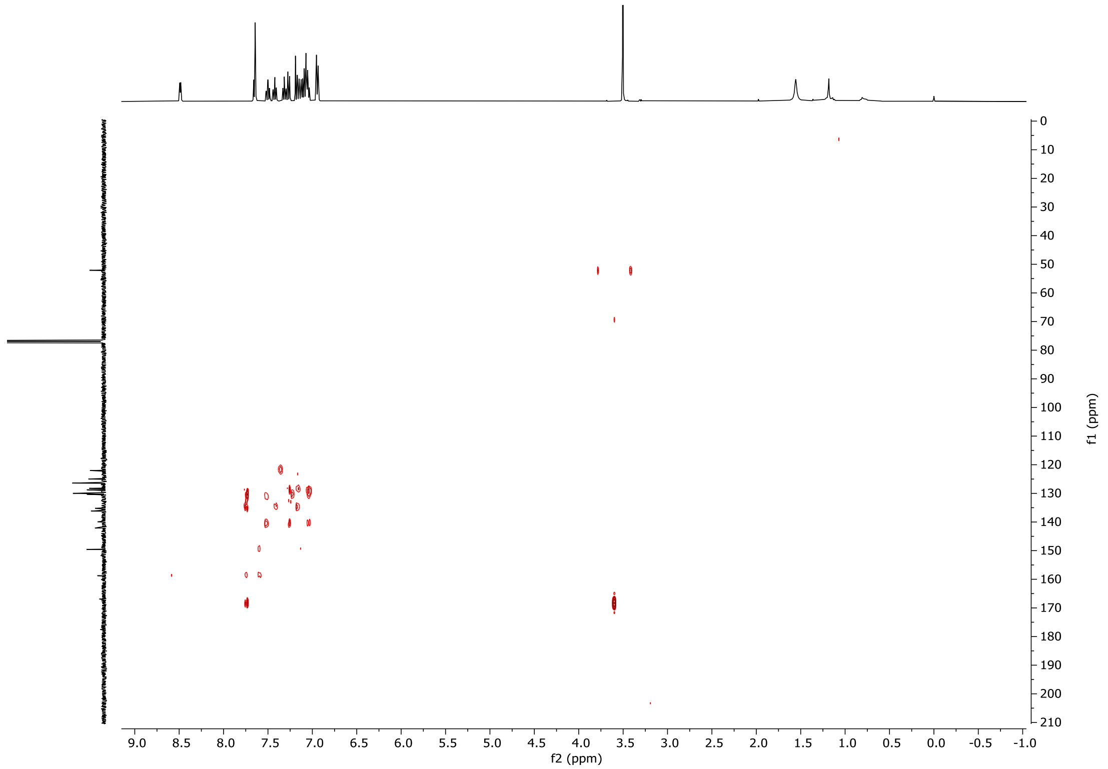

(*E*)-2-(2-(1,2-diphenylvinyl)phenyl)pyridine (5an)

^{1}H-NMR (500 MHz, $CDCl_3$)

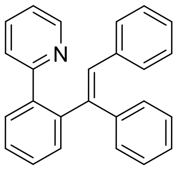

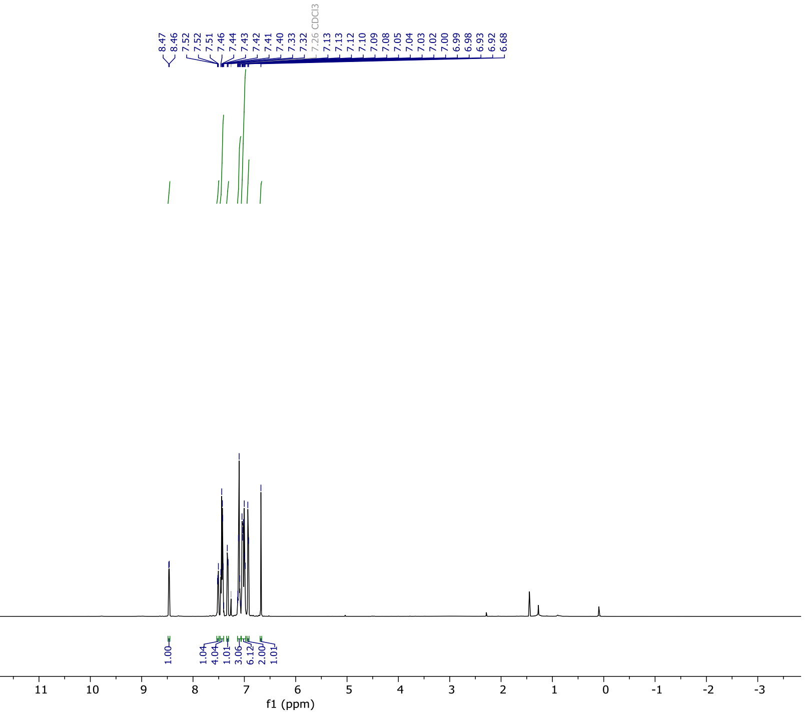

^{13}C-NMR (126 MHz, $CDCl_3$)

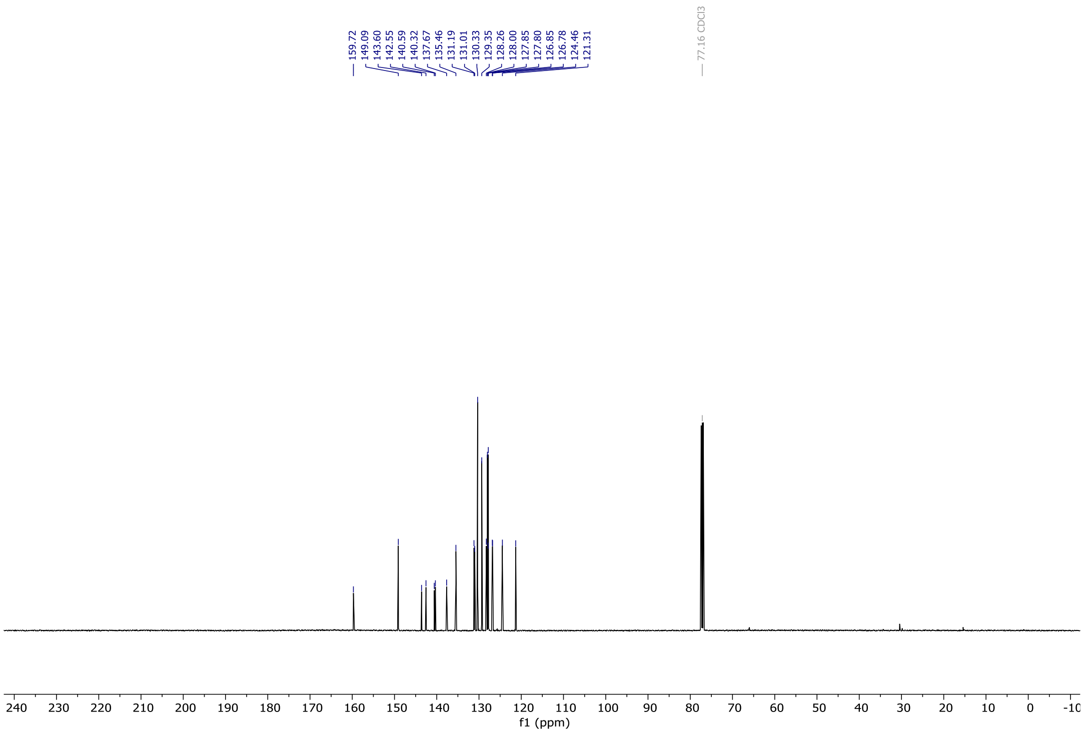

2-D NOESY-NMR (400 MHz, $CDCl_3$)

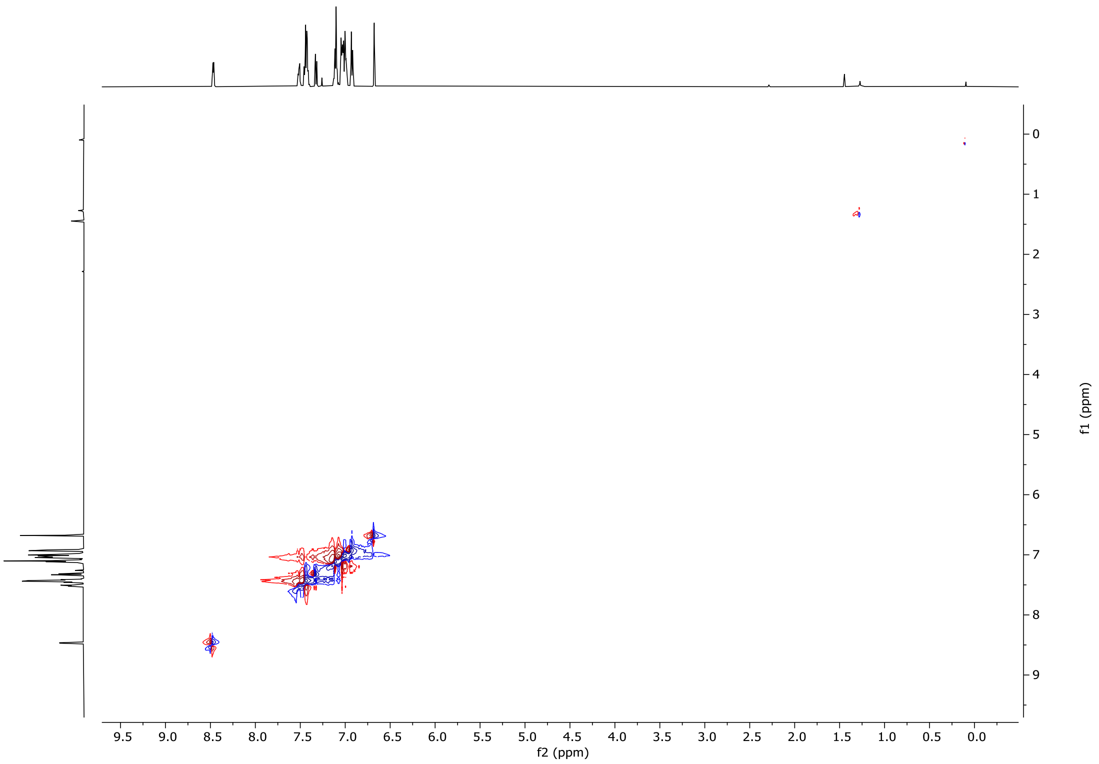

(*E*)-2-(2-(1,2-bis(4-bromophenyl)vinyl)phenyl)pyridine (5ao)

^{1}H-NMR (400 MHz, $CDCl_3$)

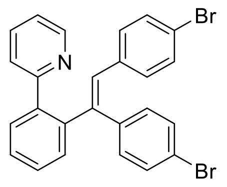

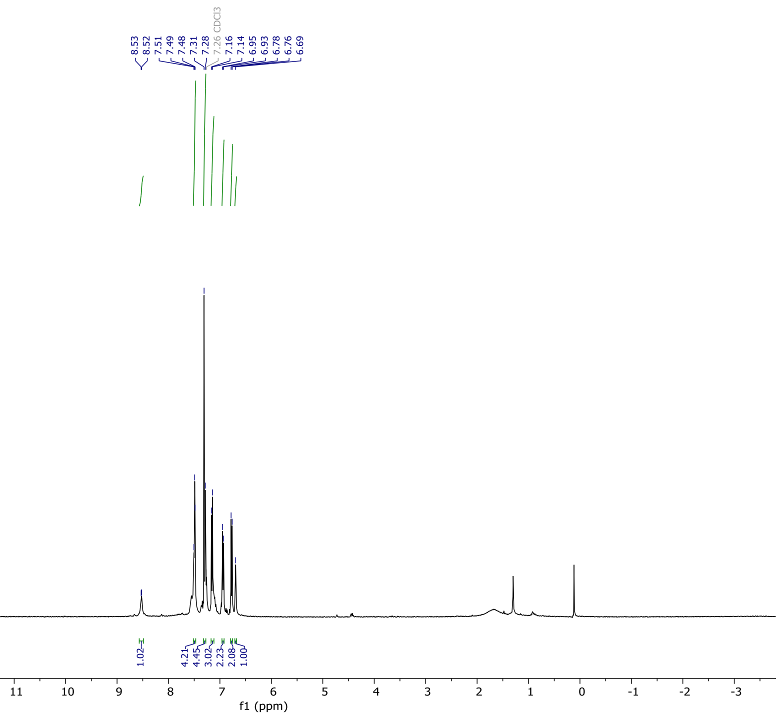

^{13}C-NMR (126 MHz, $CDCl_3$)

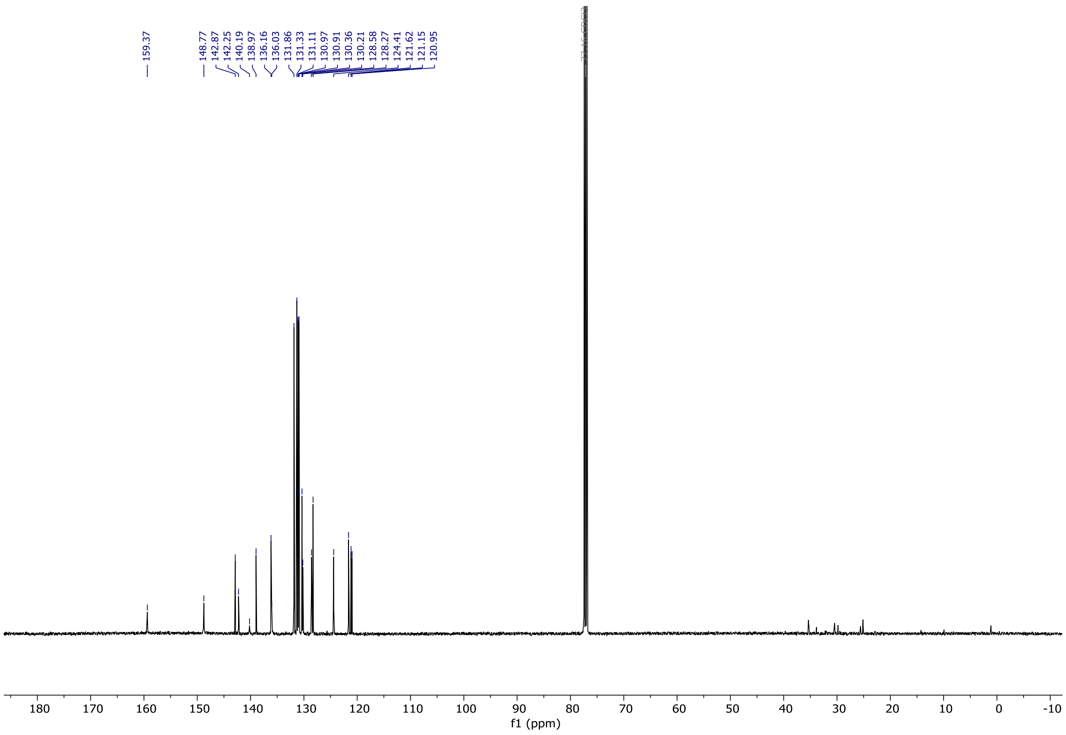

2-D NOESY-NMR (400 MHz, $CDCl_3$)

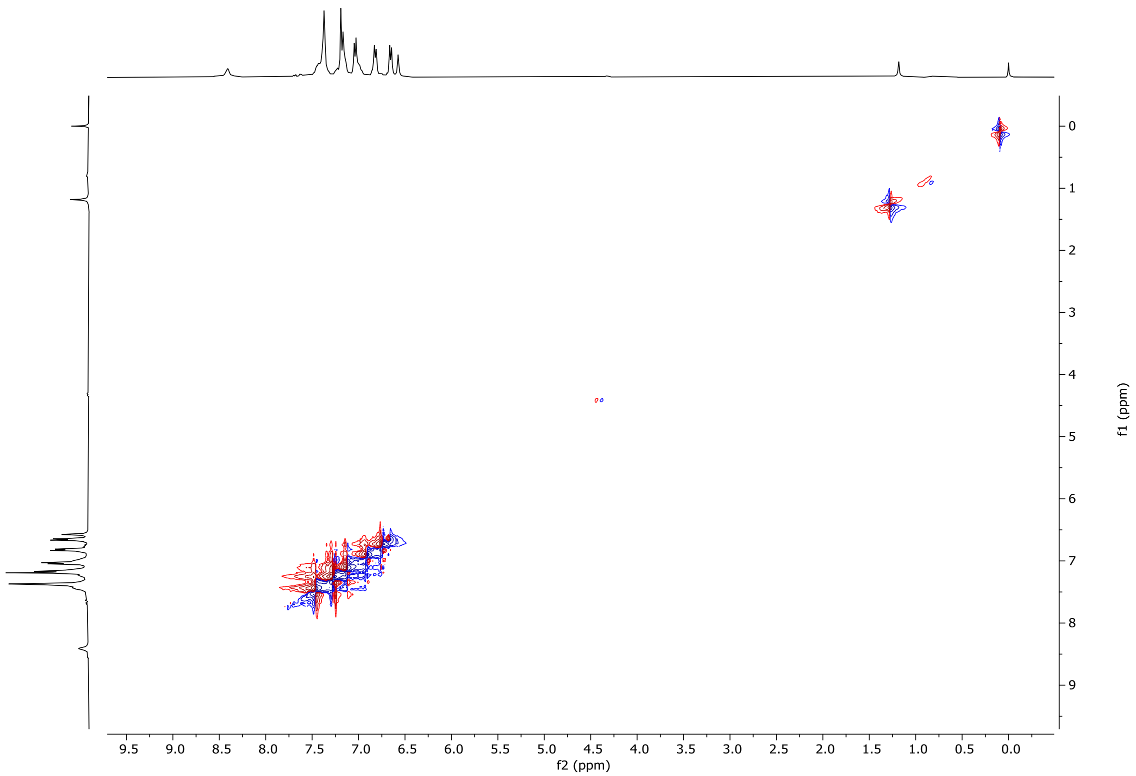

9. References

1. M. F. Farona and F. Kraus, *Inorg. Chem.*, 1970, **9**, 1700-1704
2. D. Drew, D. J. Darensbourg and M. Y. Darensbourg, *Inorg. Chem.*, 1975, **14**, 1579-1584
3. B. J. Brisdon, D. A. Edwards and J. A. White, *J. Organomet. Chem.*, 1978, **161**, 233-243
4. S. Shouheng, L. K. Teung, D. A. Sweigart, T. Y. Lee, S. S. Lee, Y. K. Chung, S. R. Switzer and R. D. Pike, *Organometallics*, 1995, **14**, 2613-2615
5. A. C. Sarapu and R.F. Fenske, *Inorg. Chem.*, 1972, **11**, 3021–3025
6. L. Zhong, Z. H. Zong and X. C. Wang, *Tetrahedron*, 2019, **75**, 2547–2552.
7. Q. L. Yang, C. Z. Li, L. W. Zhang, Y. Y. Li, X. Tong, X. Y. Wu and T. S. Mei, *Organometallics*, 2019, **38**, 1208–1212.
8. D. Yu, L. Lu and Q. Shen, *Org. Lett.*, 2013, **15**, 940–943.
9. C. Liu, N. Han, X. Song, X and J. Qiu, *Eur. J. Org. Chem.*, 2010, **2010**, 5515–5722.
10. J. Chengebroyen, M. Linke, M. Robitzer, C. Sirlin and M. Pfeffer, *J. Organomet. Chem.*, 2003, **687**, 313–321.
11. M. Alzubi, S. Arias, I. Louzao, E. Quiñoá, R. Riguera and F. Freire, *Chem. Commun.*, 2017, **53**, 8573–8576.
12. H. Samiul, N. N. Dass, K. G. Bhattacharyya and N. S. Sarma, *Mol. Cryst. Liq. Cryst.*, 2014, **592**, 149–162, 2014.
13. T. Yamamoto, T. Iwasaki, T. Morita, Yoshimi, *J. Org. Chem.,* 2018, **83**, 7, 3702–3709
14. B. Zhou, P. Ma, H. Chen and C. Wang, *Chem. Commun.*, 2014, **50**, 14558–14561.
15. N. Barsu, B. Emayavaramban and B. Sundararaju, *Eur. J. Org. Chem.*, 2017, **2017**, 4343–4510
16. J. Li, Z. Zhang, W. Ma, M. Tang, D. Wang and L. H. Zou, *Adv. Synth. Catal.*, 2017, **359**, 1717–1724.
17. T. Yoshino, H. Ikemoto, S. Matsunaga and M. Kanai, *Angew. Chem. Int. Ed.*, 2013, **52**, 2207–2211.
18. S. Wang, J. T. Hou, M. L. Feng, X. Z. Zhang, S. Y. Chen, S. Y and X. Q. Yu, *Chem. Commun.*, 2016, **52**, 2709–2712.
19. B. Zhou, H. Chen and C. J. Wang, *Am. Chem. Soc.*, 2013, **135**, 1264–1267.
20. H. Zhang, Z. Yang, J. Liu, X. Yu, Q. Wang and Y. Wu, *Org. Chem. Front.*, 2019, **6**, 967–971.
21. Y. C. Chang, S. Prakash and C. H. Cheng, *Org. Chem. Front.*, 2019, **6**, 432–436.
22. H. Wang, I. Choi, T. Rogge, N. Kaplaneris and L. Ackermann, *Nat. Catal.*, 2018, **1**, 993–1001.
23. L. A. Hammarback, A. Robinson, J. M. Lynam and I. J. S. Fairlamb, *Chem. Commun.*, 2019, **55**, 3211–3214.
